# Experiences and Lessons from a Multicountry NIDIAG Study on Persistent Digestive Disorders in the Tropics

**DOI:** 10.1371/journal.pntd.0004818

**Published:** 2016-11-03

**Authors:** Sören L. Becker, Peiling Yap, Ninon S. Horié, Emilie Alirol, Barbara Barbé, Nisha K. Bhatta, Narayan R. Bhattarai, Emmanuel Bottieau, Justin K. Chatigre, Jean T. Coulibaly, Hassan K. M. Fofana, Jan Jacobs, Prahlad Karki, Basudha Khanal, Stefanie Knopp, Kanika Koirala, Yodi Mahendradhata, Pascal Mertens, Fransiska Meyanti, Elsa H. Murhandarwati, Eliézer K. N’Goran, Rosanna W. Peeling, Bickram Pradhan, Raffaella Ravinetto, Suman Rijal, Moussa Sacko, Rénion Saye, Pierre H. H. Schneeberger, Céline Schurmans, Kigbafori D. Silué, Peter Steinmann, Harry van Loen, Kristien Verdonck, Lisette van Lieshout, Lutz von Müller, Joel A. Yao, Marleen Boelaert, François Chappuis, Katja Polman, Jürg Utzinger

**Affiliations:** 1 Swiss Tropical and Public Health Institute, Basel, Switzerland; 2 University of Basel, Basel, Switzerland; 3 Institute of Medical Microbiology and Hygiene, Saarland University, Homburg/Saar, Germany; 4 Division of Tropical and Humanitarian Medicine, Geneva University Hospitals, Geneva, Switzerland; 5 Clinical Research Centre, Geneva University Hospitals, Geneva, Switzerland; 6 Department of Clinical Sciences, Institute of Tropical Medicine, Antwerp, Belgium; 7 Department of Paediatrics and Adolescent Medicine, B P Koirala Institute of Health Sciences, Dharan, Nepal; 8 Department of Microbiology, B P Koirala Institute of Health Sciences, Dharan, Nepal; 9 Hôpital Mêthodiste de Dabou, Dabou, Côte d’Ivoire; 10 Unité de Formation et de Recherche Biosciences, Université Félix Houphouët-Boigny, Abidjan, Côte d’Ivoire; 11 Département Environnement et Santé, Centre Suisse de Recherches Scientifiques en Côte d’Ivoire, Abidjan, Côte d’Ivoire; 12 Institut National de Recherche en Santé Publique, Bamako, Mali; 13 Department of Microbiology and Immunology, KU Leuven, Leuven, Belgium; 14 Department of Internal Medicine, B P Koirala Institute of Health Sciences, Dharan, Nepal; 15 Wolfson Wellcome Biomedical Laboratories, Department of Life Sciences, Natural History Museum, London, United Kingdom; 16 Centre for Tropical Medicine, Faculty of Medicine, Gadjah Mada University, Yogyakarta, Indonesia; 17 Coris BioConcept, Gembloux, Belgium; 18 London School of Hygiene and Tropical Medicine, London, United Kingdom; 19 Department of Pharmaceutical and Pharmacological Sciences, KU Leuven, Leuven, Belgium; 20 Department of Epidemiology and Molecular Diagnostics, Agroscope Changins Wädenswil, Wädenswil, Switzerland; 21 Department of Virology, Spiez Laboratory, Federal Office for Civil Protection, Spiez, Switzerland; 22 Department of Public Health, Institute of Tropical Medicine, Antwerp, Belgium; 23 Department of Parasitology, Leiden University Medical Center, Leiden, The Netherlands; 24 Department of Biomedical Sciences, Institute of Tropical Medicine, Antwerp, Belgium; Yale School of Public Health, UNITED STATES

## Introduction

Persistent digestive disorders can be defined as any diarrhea (i.e., three or more loose stools per day) lasting for at least two weeks and/or abdominal pain that persists for two weeks or longer [1–3]. These disorders cause considerable morbidity and human suffering, and hence, are reasons why people might seek primary health care. However, in resource-constrained settings of the tropics and subtropics, accurate point-of-care diagnostics are often lacking and treatment is empiric, particularly in remote rural areas with no laboratory infrastructure. As a result, the relative contribution of selected pathogens to the syndrome of persistent digestive disorders is poorly understood, and evidence-based guidelines for patient management in different social-ecological settings are scarce [4–6].

In order to improve the clinical management of patients with disorders caused by neglected tropical diseases (NTDs), the European Commission (EC) funded a five-year study—the Neglected Infectious diseases DIAGnosis (NIDIAG) research consortium. The overarching goal of the NIDIAG consortium is to develop and validate patient-centered diagnosis–treatment guidelines for use at the primary health care level in low- and middle-income countries (http://www.nidiag.org) [3,7–9]. Emphasis is placed on three syndromes: (i) persistent digestive disorders described here; (ii) persistent fever; and (iii) neurological disorders, the latter two of which are detailed in companion pieces published in the same issue of *PLOS Neglected Tropical Diseases*.

With regard to the study on persistent digestive disorders, the main aims are (i) to identify the most important NTDs and other infectious agents that give rise to this clinical syndrome, including their relative frequency; (ii) to assess and compare the accuracy of different diagnostic methods; and (iii) to determine clinical responses to commonly employed empiric treatment options for persistent digestive disorders [9]. To this end, a case–control study has been implemented in four countries: Côte d’Ivoire and Mali in West Africa and Indonesia and Nepal in Asia. An integral part of the NIDIAG consortium is to ensure that good clinical practice (GCP) and good clinical laboratory practice (GCLP) are adhered to while conducting the studies [10,11]. A quality assurance system, which included the development and implementation of a set of standard operating procedures (SOPs), along with on-the-spot staff training and internal and external quality control activities, has been developed at the project level and introduced at each study site. The development of, and adherence to, SOPs within harmonized study protocols were considered crucial steps for maximizing the integrity of laboratory and clinical data across study settings. They also provided the basis on which quality control activities could be performed.

### For Which Procedures Have SOPs Been Developed?

For the study on persistent digestive disorders, 33 specific SOPs have been developed (Supporting Information). As summarized in Table 1, detailed steps on clinical and laboratory procedures, data management, and quality assurance were described. With regard to clinical investigations, SOPs on history taking and clinical examination, assessing inclusion and exclusion criteria, patient recruitment, and study flow were developed (S1-S6). Detailed instructions on how to perform a set of laboratory diagnostic techniques for the detection of helminth and intestinal protozoa infections were included in the laboratory SOPs. Different conventional stool microscopy techniques were combined with more recent rapid antigen detection tests to encompass a broad spectrum of potentially implicated pathogens with high diagnostic accuracy (S7-S20). An overview of the employed diagnostic methods is provided in Table 2. Pertaining to data management, SOPs on completion of case report forms (CRFs) and on various activities (such as data entry, data cleaning, querying, database locking, and backing up data) were also included. To ensure quality control, SOPs on internal quality control activities, external monitoring, and laboratory supervision visits were jointly developed for the three syndromes (S21-S33).

**Table 1:** Set of standard operating procedures (SOPs) used in the NIDIAG study on persistent digestive disorders.

Number	Type	Purpose of SOP	End user	Syndrome
1	Clinical	History taking	Site investigator	Digestive
2	Clinical	Clinical examination	Site investigator	Digestive
3	Clinical	Selection of controls without digestive syndrome	Site investigator	Digestive
4	Clinical	Specific treatment procedures (including dosage)	Site investigator	Digestive
5	Clinical	Assessing inclusion and exclusion criteria	Site investigator	Digestive
6	Clinical	Patient recruitment and patient flow	Site investigator	Digestive
7	Laboratory	Kinyoun staining technique	Laboratory technician	Digestive
8	Laboratory	Modified acid-fast staining technique	Laboratory technician	Digestive
9	Laboratory	Crypto/Giardia Duo-Strip rapid diagnostic test (RDT)	Laboratory technician	Digestive
10	Laboratory	Kato-Katz thick smear technique	Laboratory technician	Digestive
11	Laboratory	Baermann funnel concentration technique	Laboratory technician	Digestive
12	Laboratory	Mini-FLOTAC technique	Laboratory technician	Digestive
13	Laboratory	How to obtain a stool sample	Laboratory technician	Digestive
14	Laboratory	Formalin-ether concentration technique	Laboratory technician	Digestive
15	Laboratory	Koga agar plate culture	Laboratory technician	Digestive
16	Laboratory	Direct fecal smear technique	Laboratory technician	Digestive
17	Laboratory	Preparation of aliquots for molecular post-hoc testing	Laboratory technician	Digestive
18	Laboratory	Urine point-of-care circulating cathodic antigen (POC-CCA) RDT for the diagnosis of *Schistosoma mansoni*	Laboratory technician	Digestive
19	Laboratory	Diagnostic sample flow	Laboratory technician	Digestive
20	Laboratory	Urine sampling	Laboratory technician	Digestive
21	Quality	Obtaining informed consent	Site investigator	Common
22	Quality	Numbering system to be used in NIDIAG studies	Laboratory technician	Digestive mainly
23	Quality	Management of study documents	Principal investigator (PI)/site investigator/ nurse/laboratory technician	Common
24	Quality	SOP on SOPs	SOP author	Common
25	Quality	External monitoring	PI/site investigator	Common
26	Quality	Internal quality control activities	Quality manager	Common
27	Quality	Good clinical laboratory practice (GCLP) supervision visits	Quality manager	Common
28	Quality	Min/max thermometer	Laboratory technician	Common
29	Quality	Stock management	Laboratory technician	Common
30	Quality	Handling of expired and disqualified products	Laboratory technician	Common
31	Quality	Handling and storage of rapid diagnostic tests	Laboratory technician	Digestive
32	Data	Completing case report forms (CRFs)	Site investigator	Common
33	Data	Procedure for data management	Data manager	Digestive

**Table 2:** Laboratory diagnostic techniques used and internally compared in the NIDIAG study on persistent digestive disorders.

Diagnostic technique	Target pathogen(s)			
	Soil-transmitted helminths	*Schistosoma mansoni*	*Strongyloides stercoralis*	Intestinal protozoa
Direct fecal smear	(✓)	(✓)	(✓)	(✓)
Kato-Katz thick smear	✓	✓	–	–
Formalin-ether concentration	✓	✓	(✓)	✓
Mini-FLOTAC	✓	✓	–	(✓)^a^
Baermann funnel concentration	(✓)^b^	–	✓	–
Koga agar plate culture	(✓)^b^	–	✓	–
RDT for *Cryptosporidium* and *Giardia intestinalis*	–	–	–	✓^c^
POC-CCA urine cassette test	–	✓	–	–
Acid-fast staining procedure	–	–	–	✓^d^

The laboratory diagnostic techniques consisted of microscopic methods and rapid diagnostic tests (RDTs). They were used and compared within the NIDIAG study on persistent digestive disorders, placing particular emphasis on the suitability for the detection of helminths and intestinal protozoa that may give rise to persistent digestive disorders (≥2 weeks). The following grading system was employed to characterize the suitability of a certain laboratory technique for the detection of specific pathogens: ✓ suitable; (✓) partially suitable; –not suitable.

Of note, additional bacteriologic stool cultures were performed in all countries except Indonesia in case of diarrheic stool samples.

^a^Very limited published data, according to which FLOTAC techniques may detect some intestinal protozoa species (e.g., *G*. *intestinalis*), but further validation of the technique for this use is required.

^b^Hookworm larvae can be detected, in particular by culture. Additionally, hookworm larvae can be found using the Baermann technique, if the stool sample has been kept long enough for the eggs to hatch.

^c^This RDT detects only *Cryptosporidium* spp. and *G*. *intestinalis*.

^d^Acid-fast staining methods (e.g., Kinyoun stain) are particularly suitable for the detection of *Cryptosporidium* spp., *Cyclospora cayetanensis*, and *Cystoisospora belli* that are easily missed by most other microscopic diagnostic techniques.

**Fig 1:**
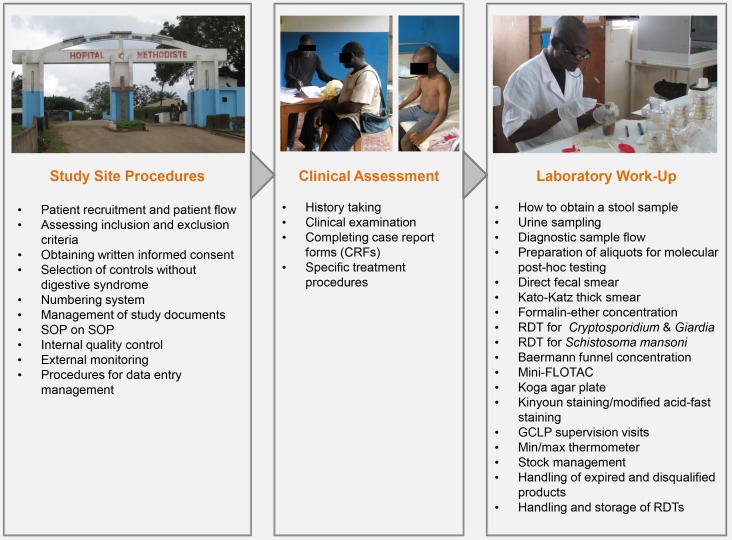
Principal elements of the NIDIAG digestive study and the respective standard operating procedures (SOPs) used.

Of note, all SOPs were developed in English (for use in Nepal) and subsequently translated into French (for use in Côte d’Ivoire and Mali) and Bahasa Indonesia (for use in Indonesia). This comprehensive set of closely interconnected SOPs—which provides guidance on all essential procedures from the first presentation of an individual at a health care center until the final processing of all patient and laboratory data—is displayed in Fig 1.

### How Was the Development of SOPs Coordinated, and Which Quality Control Measures Were Adopted?

The development and harmonization of the various SOPs was coordinated by the quality assurance group of the NIDIAG consortium and the trial management group (TMG) of the digestive syndrome study and followed a standard template and consortium-wide guidelines stipulated in the SOP entitled “SOP on SOP” (S24). This allowed different authors with varied background and writing styles to convey key messages and pass on their expert knowledge in a systematic, standardized manner for the benefit of the end user of all the SOPs. In addition, it provided clear instructions on how the SOPs should be numbered, reviewed, and approved to allow for strict version control. The authors of the SOPs were chosen from within the NIDIAG consortium, and allocation of topics was based on expertise and track record in the clinical, laboratory, data management, and quality assurance components of the study. Experts in the field, at the bench, and at the bedside carefully reviewed and revised the draft SOPs. Before the start of recruitment, local clinical and laboratory teams were trained on the set of SOPs through two hands-on workshops lasting three days each that were conducted on site by relevant experts of the NIDIAG consortium. During these workshops, feedback from the local partners was incorporated to refine the already developed SOPs, and additional SOPs were jointly developed to meet specific demands of local clinical, epidemiologic, and laboratory conditions. For example, in Indonesia, where Kinyoun staining was not available, an SOP pertaining to a slightly modified acid-fast staining technique was developed for the local team instead. Finally, once an SOP was finalized, a member of the TMG would approve it. A quality assurance member of the NIDIAG consortium was tasked to compile and keep updated the final set of SOPs and ensure that the latest versions were available on the NIDIAG intranet for distribution among the different country partners.

### Which Patient Recruitment Patterns Have Been Observed?

In clinical trials and case–control studies, it is one of the most difficult tasks to precisely define patient inclusion and exclusion criteria and to carry out the enrolment accordingly under “real life” conditions, thereby minimizing selection bias [12]. Apart from a specific study protocol and a given research question, several studies have shown that many internal and external factors may considerably influence the pace of patient recruitment in clinical trials. Many of these factors are not related to scientific issues, such as the workload for study staff who are responsible for the recruitment of patients, the distance between the study center and the place of residence of potential participants, insufficient engagement with the community, and the complexity of consent procedures prior to the start of patient enrollment [13,14]. In the NIDIAG study pertaining to persistent digestive disorders, several site assessments were carried out in the four countries before launching the larger multicountry study [15]. It was decided that a case–control design should be adopted [16–18] and that the ideal target sample size in each country would be in the range of 500 symptomatic patients and 500 asymptomatic controls. After obtaining all the necessary ethics approvals (August 2013–January 2014), extensive study document and site preparations, and GCP/GCLP training of the study site staff, the first patient was recruited in Nepal on July 30, 2014.

**Fig 2:**
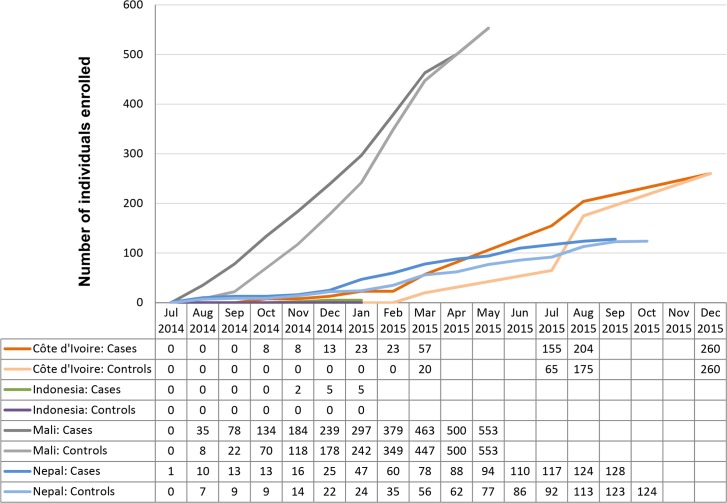
Country-specific enrolment characteristics of patients and controls in the NIDIAG study on persistent digestive disorders.

Interestingly, the recruitment of patients and controls showed considerable heterogeneity across countries. Fig 2 displays the country-specific enrolment over time. In Mali, where almost exclusively patients with persistent abdominal pain (and very few with persistent diarrhea) were recruited, the numbers of eligible patients and controls were high, and the recruitment went smoothly and without major obstacles. Indeed, a total of 553 patients and 553 matched controls were enrolled by May 2015, when the study was deemed successfully completed in Mali.

A different pattern was observed in Nepal. Patient recruitment started well in July and August 2014 but was temporarily interrupted for a period of almost three months because of protracted strikes and civil unrest that limited freedom of movement of the people. Later, patient enrolment resumed at a stable recruitment pace (with some seasonality seen) until August 2015, when nationwide strikes again affected the number of enrolled patients such that the initially targeted sample size of 500 symptomatic patients and 500 controls could not be reached.

A slow recruitment rate was observed in Côte d’Ivoire during the first months of the study, which was mainly explained by the fact that few eligible patients presented to the study hospital, whereas higher numbers of patients with either persistent diarrhea or persistent abdominal pain were reported from rural, remote health care posts in the surroundings of the study hospital. Hence, the recruitment was changed from a mainly passive approach—i.e., waiting for symptomatic patients to present to the study hospital—to a more proactive enrolment strategy, which included informing health care workers in the peripheral health care institutions and villages about the purpose and scope of the study so that they could actively refer potentially eligible patients to the study team.

A similar proactive approach was adopted in Indonesia, but the number of enrolled patients with persistent digestive disorders remained very low. Hence, after five months (August 2014–January 2015), it was decided by the study country team and the TMG to interrupt the study prematurely and to modify the design. Subsequently, a large community-based survey was carried out in the study area—which employed the same suite of diagnostic methods as foreseen in the original study protocol—to improve the diagnosis of parasitic infections and to increase the understanding of the causes and the clinical features that are related to these infections in rural Indonesia. The respective ethics committees were readily informed about the necessary modifications, and approval was obtained before the start of the survey.

### What Were Key Challenges During Patient Recruitment?

During the course of the NIDIAG digestive study, several challenges were encountered that underscore why it remains an ambitious goal to perform studies on neglected clinical syndromes in the tropics. First, most study investigators reported difficulties in recruiting patients with persistent diarrhea as defined by the World Health Organization (WHO)—i.e., any diarrhea lasting for at least 14 days [2]. Indeed, while long-lasting diarrheal diseases were perceived as a relevant clinical problem in all four study countries, many patients with persistent diarrhea had previously either taken empiric treatment (i.e., antibiotics, antiparasitics, or traditional medicines) or consulted a health care professional (e.g., village health center or local pharmacy). In this context, it is important to note that antidiarrheal medication and anti-infective drugs are regularly available as over-the-counter drugs without prescription in many low- and middle-income countries and are thus easily accessible [19,20]. Such medical and pharmaceutical interventions probably led to partial, transitory improvement in some patients, but the digestive symptoms returned after variable periods of intermittence in a considerable proportion of study participants. However, such patients frequently failed to meet the actual inclusion criteria because the onset of diarrheal symptoms after 2 days without diarrhea was defined as a new episode [9], and some of them could thus not be included.

Second, diarrhea and abdominal pain are relatively vague, subjective symptoms, and their reported severity depends on the perceived concepts of illness of the individual patient [21]. Previous studies have shown that the length of the recall period is inversely related to the accuracy and reliability of reported patient complaints and that less severe episodes of diarrhea can be up to 40% underreported, with a recall period of one week [22]. Furthermore, there is evidence from resource-constrained settings that diarrhea is less likely to be reported as a perceived health problem if it is common in a specific area [23,24]. For obvious reasons, “persistent abdominal pain” is even more difficult to define and to objectively retrace. The application of a standardized medical examination as part of the routine workup in the NIDIAG digestive study was thus considered an essential tool to complement data gathering and to counterbalance inaccuracies obtained by self-assessed morbidity reports.

Third, the NIDIAG digestive study was noninterventional, and study site–specific factors had thus to be considered. In Nepal, for instance, there are considerable government-run mass drug administration (MDA) campaigns that may have led to a decrease of helminthiasis-associated diarrheal diseases. Indeed, biannual deworming with albendazole for children aged 12–59 months has been carried out along with vitamin A supplementation since 1999, and albendazole is given twice a year to children attending grades one through ten of all public and private schools. Since 2005, albendazole has also been administered along with diethylcarbamazine citrate (DEC) as part of the efforts to eliminate lymphatic filariasis, and Nepal is expected to complete six rounds of MDA in 2018. Finally, pregnant women are also given albendazole during their second trimester. Hence, nearly all age groups in Nepal may potentially receive this anthelmintic drug through one of these MDA campaigns, and it will be interesting to see whether the NIDIAG study results confirm a beneficial effect on helminth-associated persistent digestive disorders.

Fourth, the etiology of persistent diarrhea and persistent abdominal pain is manifold and not limited to infectious causes. Autoimmune disorders, inflammatory diseases, neoplasms, and specific food intolerances may, among others, also lead to persistent intestinal disorders [25]. The researchers and clinicians on site experienced that this fact needs to be explained in detail to symptomatic patients without an identified pathogen, and further investigations or referral to additional diagnostics should be initiated promptly. Of note, the detection of a certain pathogen in a stool or urine sample does not necessarily mean that the patient’s symptoms are actually linked to this finding [26]. As the proportion and quantity of encountered pathogens may vary considerably from one setting to another, the NIDIAG study employed a case–control approach to elucidate pathogen-specific attributable fractions to the syndrome of persistent digestive disorders [27,28].

Controls were recruited from the same study centers as the symptomatic patients and were further matched by age group and sex to the symptomatic cases. It proved to be difficult to obtain consent of individuals to act as controls because they were frequently wondering why someone would be interested in analyzing stool and urine samples of healthy individuals. While careful explanation—being particularly sensitive to potential cultural barriers—helped to clarify this, some individuals still considered stool samples as very personal, intimate specimens and refused to participate. Indeed, the Nepalese study sites observed that it was very difficult to include asymptomatic patients who were unrelated to a symptomatic patient, while it was relatively straightforward to obtain consent from friends, relatives, or neighbors of symptomatic cases with persistent diarrhea, potentially because these individuals shared similar exposure characteristics (e.g., source of drinking water) with the symptomatic patient and wanted to know whether they were found to carry the same intestinal pathogens.

### Have Challenges of Specific Laboratory Techniques Been Observed, and Which Solutions Have Been Adopted?

Two main areas of laboratory-associated challenges were identified during the NIDIAG digestive study: (i) general infrastructure and equipment and (ii) the conduct of the diagnostic laboratory tests. First, it was noted during the pre-study field visits that the available laboratory infrastructure in the study countries was, at most sites, not sufficient to accommodate such a broad diagnostic test panel as indicated by the NIDIAG study protocol [3]. Indeed, there was no laboratory that had routinely employed all of the methods before the onset of the study. Hence, workshops were held to familiarize study staff with the techniques and to harmonize procedures across study countries. Additionally, many efforts were required to establish an internal and external quality assurance and monitoring system so that GCP/GCLP standards were met. It is important to note that such preparatory work is essential to ensure the proper conduct of scientific studies, but the significant human, logistic, and financial resources required are usually not considered sufficiently beforehand by both scientists and funding organizations [29]. In Indonesia, the complete laboratory infrastructure had to be set up in a remote area of an island where the study center was located, which required several weeks of construction work. A short video showing the different study procedures as well as the laboratory infrastructure before and after the onset of the NIDIAG study in Maluku Tengah can be found in the supplementary material of this manuscript (S34). Of note, even minor logistic issues may have a profound impact. In Côte d’Ivoire and Nepal, for example, the absence of a functioning ventilation and climate control system in the laboratories led to several short delays in recruitment and specimen processing, as stool samples are unpleasant to work with and the study was thus not initiated before the ventilation systems were fixed.

Study site investigators frequently reported that the volume of a single stool sample was smaller than the amount stipulated in the respective SOP and was thus not sufficient to perform all required laboratory tests. Indeed, an amount of approximate 80 g of stool was required to run all tests (Table 2), as several diagnostic techniques (such as the Baermann funnel concentration for the diagnosis of *Strongyloides stercoralis*) require a large amount of stool. In such cases, a second sample had to be obtained from participants, but some, especially children, were not always able to provide the required amount and sometimes did not come back to provide the requested second sample.

Because of the host of different laboratory tests, the processing of a sample took approximately two to three hours, and it would have been ideal to collect stool specimens in the early morning and analyze them immediately in the laboratory. However, this was not always possible as the sites of patient enrolment were sometimes quite distant from the actual laboratory (e.g., the Dhankuta study site in Nepal) or the study laboratories had to send parts of the sample to reference laboratories (e.g., in Côte d’Ivoire and Mali, for stool bacteriology in case of persistent diarrhea). Such sample transfer had to be organized and carried out by couriers, such that laboratory staff sometimes worked until late in the evening when a sample arrived only in the afternoon.

With regard to difficulties associated with specific laboratory techniques, few deviations were observed, and the study investigators complied very well with the various SOPs. This observation was largely attributed to the two in-depth preparatory workshops that had taken place prior to the start of the study. Some of the remaining challenges experienced and potential solutions to overcome these are presented in Table 3.

**Table 3:** Diagnostic challenges encountered during the NIDIAG study on persistent digestive disorders and proposed solutions.

Diagnostic test	Problem	Possible reason	Solution
RDT for *Cryptosporidium* and *Giardia*	Faintly positive test line that is hard to interpret as either a positive or negative test result	An inaccurate volume of stool sample may have been used	While an exact amount of liquid stool could easily be taken via pipettes, this was less standardized for solid samples. All positive or faintly positive RDT results were documented by photography, and results should be compared to subsequent microscopic and molecular diagnostics
RDT for *Cryptosporidium* and *Giardia*	During internal quality control, a *Cryptosporidium*-positive stool sample led to inconsistent results upon RDT application	False-negative results were exclusively observed on expired or nearly expired RDTs	Strict adherence to the indicated expiration dates of RDTs in clinical studies and routine diagnostics
Formalin-ether concentration	Difficult microscopic reading of stool samples following formalin-ether concentration	Questionable quality of the locally obtained ether	Identify alternative provider for ether and other chemical products required for analysis (proved to be difficult in some study countries)
Mini-FLOTAC	Leakage of one flotation chamber	The utilized Mini-FLOTAC apparatus can be reused after disinfection. However, the washing procedure may influence the stability of the flotation chambers	Apply vaseline on the septum or partition of the Mini-FLOTAC to prevent leakage

## Conclusions

As the NIDIAG project draws to an end, we feel that experiences and lessons learnt must be shared with the broader research community, clinicians, and disease control managers in countries where digestive disorders due to NTDs remain an important public health issue (Box 1). The current set of field-tested and ready-to-use SOPs has been implemented successfully in various sites, including those located in areas with restricted resources. We hope that providing open access to this large compilation of SOPs and highlighting key challenges met during the implementation and the conduct of the NIDIAG study on persistent digestive disorders might assist in further training and capacity building on all aspects of patient recruitment, clinical and diagnostic workup within GCP/GCLP standards, data management, and quality control, and thus improve the clinical diagnostic algorithms for patients suffering from NTDs. We look forward to experiences and lessons by other groups pursuing syndromic approaches to advance the diagnosis and clinical management of NTDs among neglected populations.

**Box 1. Key Learning Points from the Multicountry NIDIAG Study on Persistent Digestive Disorders in the Tropics**

- There is a need for studies investigating the etiology, diagnosis, and management of common clinical syndromes such as persistent digestive disorders in the tropics. Easily applicable, evidence-based diagnosis–treatment algorithms could substantially improve the clinical management of such syndromes at the primary health care level.
- The implementation of a quality assurance system is crucial for conducting multicenter clinical studies in resource-restricted settings, and it remains an ambitious goal to perform studies that fully comply with GCP and GCLP.
- Study documents such as SOPs and CRFs should be jointly developed and validated to harmonize procedures across study sites. Such field-tested tools are an important resource for researchers and health care staff and should thus be made publicly available.
- In a study on persistent digestive disorders, which was carried out by the NIDIAG consortium, perceived challenges were the prolonged recall period (≥14 days) for the identification of individuals with persistent abdominal pain and persistent diarrhea and the considerable heterogeneity seen regarding the recruitment pace of patients and controls in the four study countries.

## Supporting Information

S1 Clinical SOP History taking. (PDF)

S2 Clinical SOP Clinical examination. (PDF)

S3 Clinical SOP Selection of controls without digestive syndrome. (PDF)

S4 Clinical SOP Specific treatment procedures (including dosage). (PDF)

S5 Clinical SOP Assessing inclusion and exclusion criteria. (PDF)

S6 Clinical SOP Patient recruitment and patient flow. (PDF)

S1 Laboratory SOP Kinyoun staining technique (used in Côte d’Ivoire, Mali, and Nepal). (PDF)

S2 Laboratory SOP Modified acid-fast staining technique (used in Indonesia). (PDF)

S3 Laboratory SOP Crypto/Giardia Duo-Strip rapid diagnostic test (RDT). (PDF)

S4 Laboratory SOP Kato-Katz thick smear technique. (PDF)

S5 Laboratory SOP Baermann funnel concentration technique. (PDF)

S6 Laboratory SOP Mini-FLOTAC technique. (PDF)

S7 Laboratory SOP How to obtain a stool sample. (PDF)

S8 Laboratory SOP Formalin-ether concentration technique. (PDF)

S9 Laboratory SOP Koga agar plate culture. (PDF)

S10 Laboratory SOP Direct fecal smear technique. (PDF)

S11 Laboratory SOP Preparation of aliquots for molecular post-hoc testing. (PDF)

S12 Laboratory SOP Urine point-of-care circulating cathodic antigen (POC-CCA) RDT for the diagnosis of *Schistosoma mansoni*. (PDF)

S13 Laboratory SOP Diagnostic sample flow. (PDF)

S14 Laboratory SOP Urine sampling. (PDF)

S1 Quality SOP Obtaining informed consent. (PDF)

S2 Quality SOP Numbering system to be used in NIDIAG studies. (PDF)

S3 Quality SOP Management of study documents. (PDF)

S4 Quality SOP SOP on SOP. (PDF)

S5 Quality SOP External monitoring. (PDF)

S6 Quality SOP Internal quality control activities. (PDF)

S7 Quality SOP Good clinical laboratory practice (GCLP) supervision visits. (PDF)

S8 Quality SOP Min/max thermometer. (PDF)

S9 Quality SOP Stock management. (PDF)

S10 Quality SOP Handling of expired and disqualified products. (PDF)

S11 Quality SOP Handling and storage of rapid diagnostic tests (RDTs). (PDF)

S1 Data management SOP Completing case report forms (CRFs). (PDF)

S2 Data management SOP Procedure for data management. (PDF)

S1 Video The NIDIAG study site in Maluku Tengah, Indonesia. (7Z)
