# Supplementary material for: Experiences and Lessons from a Multicountry NIDIAG Study on Persistent Digestive Disorders in the Tropics
Source: PLoS Negl Trop Dis. 2016 Nov 3;10(11):e0004818. doi: 10.1371/journal.pntd.0004818 (PMC5094778; doi:10.1371/journal.pntd.0004818)
Supplement: S1 Clinical SOP — (PDF) [file pntd.0004818.s001.pdf]

	**Title : Standard Operating Procedure (SOP) for History Taking**
	**Diagnosis of neglected tropical diseases (NTDs) in patients with persisting digestive disorders (≥2 weeks) in Côte d'Ivoire, Indonesia, Mali and Nepal.**

1. Scope and application

This SOP applies to all patients included in the digestive syndrome study. It describes the procedure of history taking on demographic data and disease course.

2. Responsibilities

Function	Activities
Country Principal Investigator	- Organization of the training of the site investigators regarding the history taking<br>- Control of the adequate CRF filling according to this SOP
Site Principal Investigator	- Performance of history taking of all enrolled patients according to this SOP<br>- Data registration according to this SOP<br>- Supervision of the adequate history taking
Site Investigator	- Performance of comprehensive and standardized history taking of all enrolled patients according to this SOP<br>- Registration of initial clinical data and follow-up data according to this SOP
Monitor	- Verification that history taking and follow-up of enrolled patients are performed according to this SOP<br>- Verification that Good Clinical Practices are respected<br>- Verification that the CRF is adequately filled

3. Material

Table / desk, chairs, blouse, towels, trash can, calendar, black pen, paper CRF.

4. Procedure

1. Check identity:
   - Gender
   - Birth date or Age in years
   - Place of residence (village / district)
2. Ask to specify:
   - Medical history (resolved health problems relevant to mention such as appendectomy): briefly describe and notify end date.
   - Ongoing medical problems (active disease with ongoing treatment such as diabetes) other than digestive disorders: briefly describe and notify start date and current treatment.
   - Antecedent of allergy to drug or other product.
3. Clinical evaluation of current symptoms:
   - Verify systematically the presence, absence (or « unknown »), during the clinical examination, of all symptoms mentioned in the CRF and specify the characteristics whenever requested (type, frequency, intensity, localization, diffusion, duration, …).

N.B. If the question « week » or « day » is not applicable, fill "0" (zero) in the specific item.

4. Evaluation of concomitant treatment:
   - Ask whether the participant has received drugs for the current illness (persistent digestive disorder) and if yes whether the treatment is still ongoing or not. Fill the treatment form with drugs already taken or ongoing for the PERSISTENT DIGESTIVE DISORDERS.
   - Verify systematically the presence, absence (or « unknown »), during the clinical assessment, of the recent exposure (< 4 weeks before the onset of illness) to specific events mentioned in the CRF.

5. Document History

**Revision**	
SOP-WP2-CLIN-11 V1.1-03Dec2013	Initial version by Moussa Sacko and colleagues in Mali
SOP-WP2-CLIN-11 V2.1-09Dec2013	Review by Emmanuel Bottieau
SOP-WP2-CLIN-11 V3.1-28Feb2014	Approval by François Chappuis
SOP-WP2-CLIN-11 V3.0-16May2014	Translation into English by Emmanuel Bottieau

**Name and function**	**Date**	**Signature**
Auteur		
Moussa Sacko and colleagues	03.12.2013	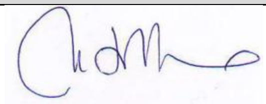
Revu par		
Emmanuel Bottieau	09.12.2013	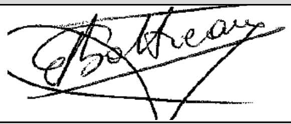
Approuvé par		
Ninon Horié	19.05.2014	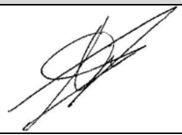
