# Supplementary material for: Experiences and Lessons from a Multicountry NIDIAG Study on Persistent Digestive Disorders in the Tropics
Source: PLoS Negl Trop Dis. 2016 Nov 3;10(11):e0004818. doi: 10.1371/journal.pntd.0004818 (PMC5094778; doi:10.1371/journal.pntd.0004818)
Supplement: S2 Clinical SOP — (PDF) [file pntd.0004818.s002.pdf]

<u>Title :</u> Standard Operating procedure (SOP) for Clinical Examination

Diagnosis of neglected tropical diseases (NTDs) in patients with persisting digestive disorders (≥2 weeks) in Côte d'Ivoire, Indonesia, Mali and Nepal.

1. Scope and application

This SOP applies to all patients included in the NIDIAG study on digestive syndrome who will have to undergo a thorough clinical examination and a targeted digestive evaluation. The purpose of the study is to identify several clinical and laboratory predictors discriminating the NTDs and priority infectious diseases that contribute to the digestive syndrome. Consequently, a comprehensive and standardized clinical examination, in particular digestive, is crucial for this research. Since most physicians do not always explore precisely and systematically the patients in a standardized way, the section « Physical Examination » has been developed in this SOP with detailed definitions and explanations. The purpose is to decrease as much as possible the subjectivity and approximations in the data collection and interpretation by different investigators. The CRF has been elaborated according to the logical framework of the clinical approach and as a whole has been designed in the best « self-explanatory" way. **It is however obvious that patient care has the highest priority! If a patient presents with severe or life-threatening manifestations, all clinical efforts must be done to stabilize him first before any administrative or research documentation is carried out**.

2. Responsibilities

**Function**	**Activities**
Country Principal Investigator	- Organization of the training of the site investigators regarding the clinical examination<br>- Control of the adequate CRF filling according to this SOP

Site Principal Investigator	- Performance of comprehensive and standardized clinical/digestive examination of all enrolled patients according to this SOP<br>- Data registration according to this SOP<br>- Supervision of the adequate performance of clinical/digestive examination by the co-investigators
Site Investigator	- Performance of comprehensive and standardized clinical/digestive examination of all enrolled patients according to this SOP<br>- Registration of initial clinical data and follow-up data according to this SOP
Monitor	- Verification that the clinical/digestive examination of included patients is performed according to this SOP<br>- Verification that good clinical practices are respected<br>- Verification that the CRF is adequately filled

3. Material

Examination table, chairs, disposable paper, digital thermometer, stethoscope, flashlight, tongue depressor, white coat, clock, weight/height scale, tape measure, reflex hammer, antiseptic, towels, disinfectant, cotton, gloves, alcohol, trash can, paper CRF, black pen and CRF copy.

4. Procedure

A. Day of admission or Day of first outpatient consultation (Day 0)

- Check that inclusion criteria are all fulfilled (see SOP-WP2-CLIN-03) and that the inform consent has been obtained (see SOP-WP6-DOC-01).

- Assign a unique Identification Number "ID" to the patient and write it on each CRF page.

  Patient number : | | | | | |-| | |

- Perform a detailed history taking (according to the CRF),

- Continue with the physical examination and enter the information directly in the CRF, in the section "Physical Examination J0".

1. Measure the weight in kg,
2. Measure the height in cm,
3. Cover the table with disposable paper or clean sheet,
4. Undress the participant and install him on the table or on parent's knees (if child),
5. Take the axillar temperature (notify in º C) with the digital thermometer,
6. Count the breath rate (number of respiratory cycles during one minute),
7. Take the pulse rate (per minute) in the external 1/3 of right or left radius at the level of the wrist,
8. Measure the blood pressure and notify in mmHg,
9. Inspect to look for:
    a. Cachexie
    b. Pallor
    c. Localized skin rash : if present, specify localization (face, arms, legs, trunk, …) and characteristics: macular (flat rash); papular (slight elevation); vesicular(with serous fluid); purpura / petechiae (not disappearing with glass pressure)
    d. Generalized skin rash : if present, specifiy characteristics (see here above)
    e. Localized edema; if present specify localization
    f. Generalized edema (hydrops)
    g. Abnormality of ears, throat, nose, eyes, genitalia (comment if necessary)
10. Palpate (or percuss) in search of :
    a. Dehydration : if present describe (moderate or severe)
    b. Localized lymphadenopathy : if present localize (cervical, axillar, inguinal, submaxillar) and measure in cm
    c. Generalized lympadenopathies
    d. Hepatomegaly : if present, measure in cm under the ribs
    e. Splenomegaly : if present, measure in cm under the ribs
    f. Abdominal pain : if present, describe (localization, intensity, defense or rebound)
    g. Lumbar pain : if present, specify localization (left/right)
    h. Other signs: if present, describe

11. Listen in search of :
    a. Abdominal sound; specify/describe if abnormal sounds or not
    b. Crackles or wheezes, or thoracic dullness
    c. Heart murmur (to describe)

- Obtain all biological samples and send them to the laboratory for processing and preservation (see SOP-WP2-LAB-59).

B. Visits 2 and 3

- Register the vital signs, the **new** symptoms or signs and evaluate the clinical evolution compared to the initial assessment (improvement, stabilization, deterioration) in the section "Clinical Evaluation" of the CRF.
- At discharge (or at the last outpatient visit), register the vital signs, the residual symptoms and signs with the initial assessment (resolved, resolved with sequel, non-resolved/improved, non-resolved/stabilized, non-resolved/deteriorated, death, left without authorization) in the section "Clinical Evaluation at Discharge / Last Outpatient Visit" of the CRF.

Additional notes

- Maintain a registry for the appointments and give written appointment dates to all patients, at Day 0 and at each follow-up visit and at discharge for hospitalized patients.
- During the initial/discharge/follow-up assessments, register all administered treatments in the « Medication Form » at the end of the CRF.

5. Records and Archives

**Appendices & Forms to complete**	
**Document**	**Sections**
CRF	History Taking D0<br>Physical Examination D0

	Clinical Evolution (patient evaluation)<br>Final Evaluation/Last Outpatient Visit<br>Medication Form

6. Document History

**Revision**	
SOP-WP2-CLIN-12 V1.1-03Dec2013	Initial version by Moussa Sacko and colleagues in Mali
SOP-WP2-CLIN-12-V2.1-09Dec2013	Review by Emmanuel Bottieau
SOP-WP2-CLIN-12-V3.1-28Feb2014	Approval by François Chappuis
SOP-WP2-CLIN-12-V3.0-16May2014	Translation into English by Emmanuel Bottieau

**Nom et fonction**	**Date**	**Signature**
Auteur		
Moussa Sacko et collègues	03.12.2013	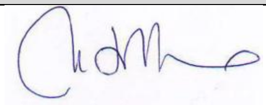
Revu par		
Emmanuel Bottieau	09.12.2013	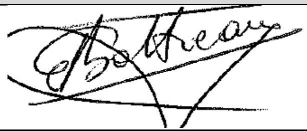
Approuvé par		
Ninon Horié	19.05.2014	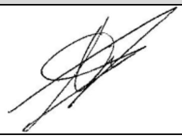
