# Supplementary material for: Experiences and Lessons from a Multicountry NIDIAG Study on Persistent Digestive Disorders in the Tropics
Source: PLoS Negl Trop Dis. 2016 Nov 3;10(11):e0004818. doi: 10.1371/journal.pntd.0004818 (PMC5094778; doi:10.1371/journal.pntd.0004818)
Supplement: S3 Clinical SOP — (PDF) [file pntd.0004818.s003.pdf]

	**SOP: Procedure for selection of controls without digestive disorders**
	Study: Diagnosis of neglected tropical diseases (NTDs) in patients presenting with persistent digestive disorders (≥2 weeks) in Côte d'Ivoire, Indonesia, Mali and Nepal.

1. Scope and application

In case of persistent digestive disorders, there is a considerable chance of finding a target 'digestive pathogen' that is just an 'innocent bystander' and that does not play a causal role in the aetiology of the symptomatology. For this reason, a control group is included in the persistent digestive disorders protocol, to better interpret causality. This SOP describes the procedure for selection of controls within the NIDIAG 'digestive' study.

2. Responsibilities

Function	Activities
Country PI	- Organizes/ensures proper training of site investigators for recruitment of controls<br>- Monitors the proper filling-in of CRFs in accordance with the present SOP
Site investigator	- Recruitment of a control to match a case enrolled in the study, in accordance with the present SOP<br>- Record keeping, as provided in this SOP<br>- Supervision of the proper execution of recruiting a control
Monitor	- Check that the recruitment of a control is made in accordance with this SOP<br>- Verify that GCP guidelines are followed<br>- Verify that CRF is correctly filled in

3. Procedure

- For each patient included in the study, enrol one asymptomatic control without persistent digestive disorders or any other gastrointestinal complaint (e.g. acute diarrhoea), presenting

at the same hospital or outpatient facility as the case (e.g. consulting for vaccination, see list below). If the patient was (actively) referred to the hospital by one of the peripheral health facilities in the catchment area, the control will be (actively) selected through the same or a similar peripheral health facility.

The controls can be enrolled if presenting without any digestive symptoms, e.g. for the following reasons or in the following hospital departments:

- Routine check-up
- Vaccination
- Ophthalmology
- Cardiology
- Neurology
- Ear-nose-throat
- Dentistry
- Paediatrics
- Routine consultations during pregnancy
- Outpatient clinic visits for patients with chronic diseases on regular treatment (Diabetes mellitus, HIV infection, asthma, pulmonary tuberculosis ...)
- Post-surgery (e.g. fracture surgery, NOT visceral surgery)

- To each enrolled patient, match one control by age stratum (1-5, 6-18, >19 years), sex, and preferably geographical location of residence (see 6.4 for further details).
- Record (in CRF) the place of recruitment (hospital/ health centre/ village).
    - ✓ If hospital/health centre, record reason for consultation (in CRF).
    - ✓ Explain to the control in detail the study purpose and procedures, and request for informed consent.
    - ✓ After informed consent has been obtained, assess inclusion/exclusion criteria (see SOP).
    - ✓ If the patient meets one of the inclusion criteria and none of the exclusion criteria, assign a patient identification number to the control.
- The controls will be submitted to the same diagnostic procedures and evaluation methods as the cases.
- Find a matched control for each case within a maximum of two months.

4. Document History

**Revision**	
SOP-WP2-CLIN-13- V1.0-03Dec2013	First final version by Moussa Sacko & Renion Sayé
SOP-WP2-CLIN-13-V1.0-02.04.2014	Revision by Katja Polman and others
SOP-WP2-CLIN-13-V1.0-18.05.2014	Approval by Jürg Utzinger

Name and function	Date	Signature
Author		
Moussa Sacko & Renion Sayé	03.12.2013	
Revised by		
Katja Polman (together with Asian workshop members)	02.04.2014	
Approved by		
Jürg Utzinger	24.05.2014	
