# Supplementary material for: Experiences and Lessons from a Multicountry NIDIAG Study on Persistent Digestive Disorders in the Tropics
Source: PLoS Negl Trop Dis. 2016 Nov 3;10(11):e0004818. doi: 10.1371/journal.pntd.0004818 (PMC5094778; doi:10.1371/journal.pntd.0004818)
Supplement: S4 Clinical SOP — (PDF) [file pntd.0004818.s004.pdf]

	**SOP Title:** Specific treatment procedures (including dosage)
	**Study title**: Diagnosis of neglected tropical diseases (NTDs) in patients presenting with persistent digestive disorders (≥2 weeks) in Côte d'Ivoire, Indonesia, Mali and Nepal.

1. Scope and application

This procedure provides a guideline on the treatment of specific diagnosed pathogens in the work-up of patients and controls. General recommendations are summarized and pathogen-specific guidelines are displayed.

2. Responsibilities

Function	Activities
Study physician	• Provide adequate treatment to infected patients<br>• Adapt your treatment plan to the individual characteristics of the patient, if need be (e.g. in case of pregnant women)

3. General considerations

- NIDIAG is a non-interventional study. Hence, the type of prescribed treatment (e.g. empiric medication in case of persistent diarrhoea) is not influenced by the study team, although it should be limited to antibiotics (ofloxacine, erythromycin, etc.) or antiparasitic agents (metronidazole) depending on the treatment history. All prescribed medication needs to be well documented in the "Medication Form" of the CRF.
- When a certain pathogen is diagnosed in a symptomatic patient, specific treatment will be offered free of charge to the patient. The choice of the specific medication will be guided by national guidelines, common clinical experience and existing evidence on the most effective essential drugs that are available in the study countries.
- Symptomatic patients and asymptomatic controls should be treated when diagnosed with a helminth infection (e.g. *Strongyloides stercoralis*) to avoid later complications arising from such an infection. In case of intestinal protozoa infection (e.g. *Giardia intestinalis*) asymptomatic carriage rates are high and treatment should only be initiated in case of a clear clinical symptomatology that might be caused by this pathogen. Likewise, bacterial enteric pathogens should not be treated if found in healthy individuals.

4. Treatment recommendations

Bacteria

Campylobacter spp.	Macrolides<br>(e.g. erythromycin 4x500 mg/day for 3-5 days in adults and 12.5 mg/kg 4x per day for 3-5 days in children,<br>Or azithromycin 500mg/day for 3 days in adults or 10 mg/kg/day for 3 days in children)
Salmonella spp	Fluoroquinolones (e.g. ofloxacin 2x400 mg/day for 3-5 days or ciprofloxacin 2x500 mg/day for 3-5 days) or azithromycin 10 mg/kg/day for 3 days (children and pregnant women)
Shigella spp.	Fluoroquinolones (e.g. ofloxacin 2x400 mg/day for 3-5 days or ciprofloxacin 2x500 mg/day for 3-5 days) or azithromycin 10 mg/kg/day for 3 days (children and pregnant women)

Intestinal protozoa

Cryptosporidium parvum	No recommendation for any specific antiparasitic treatment
Cyclospora cayetanensis	Cotrimoxazole forte 160/800 2x1 tab daily for 7 days<br>(5 mg TMP + 25 mg SMZ/kg/day for 7 days in children)
Cystoisospora belli	Cotrimoxazole forte 160/800 2x1 tab daily for 7 days<br>(5 mg TMP + 25 mg SMZ/kg/day for 7 days in children)
Entamoeba histolytica	Metronidazole 500-750 mg (10-15 mg/kg) thrice a day for 7 days followed by paromomycin (10 mg/kg/8h for 7 days, if available)
Giardia intestinalis	Metronidazole (e.g. 250-500mg thrice a day for 5 days; pediatric dose: 5mg/kg 3x/day for 5 days)

Helminths

Ascaris lumbricoides	Albendazole (400 mg) or mebendazole (500 mg) as single dose
Trichuris trichiura	Albendazole (400 mg) or mebendazole (500 mg) over three consecutive days
Hookworm	Albendazole (400 mg) as single dose
Strongyloides stercoralis	Ivermectin (200 µg/kg) as single dose
Schistosoma mansoni	Praziquantel (40 mg/kg) as single dose

5. Records and archives

**Appendices & Forms for completion**	
**Number**	**Title**
NA	*NA*

6. Document History

**Revision**	
SOP WP2-CLIN-14-V1.0-02Dec2013	Initial version by Sören Becker
SOP WP2-CLIN-14-V2.0-09Dec2013	Revision by Emmanuel Bottieau
SOP WP2-CLIN-14-V3.0-23Feb2014	Revision by Sören Becker
SOP WP2-CLIN-14-V4.0-28Feb2014	Approval by François Chappuis

**Name and function**	**Date**	**Signature**
Author		
Sören L. Becker	02.12.2013	[signature]
Reviewed by		
Emmanuel Bottieau<br>Sören L. Becker	09.12.2013<br>23.02.2014	[signature]
Approved by		

François Chappuis	28.02.2014	
