# Supplementary material for: Experiences and Lessons from a Multicountry NIDIAG Study on Persistent Digestive Disorders in the Tropics
Source: PLoS Negl Trop Dis. 2016 Nov 3;10(11):e0004818. doi: 10.1371/journal.pntd.0004818 (PMC5094778; doi:10.1371/journal.pntd.0004818)
Supplement: S5 Clinical SOP — (PDF) [file pntd.0004818.s005.pdf]

| nidiag | **SOP title:** Assessing inclusion and exclusion criteria<br><br>Study: Diagnosis of neglected tropical diseases (NTDs) in patients presenting with persistent digestive disorders (≥2 weeks) in Côte d'Ivoire, Indonesia, Mali and Nepal. |
|---|---|

## 1. Scope and application

This SOP applies to all the patients referred to the study physician in all study sites involved in the study of persistent digestive disorders in the NIDIAG study. It describes the procedure for checking inclusion and exclusion criteria.

## 2. Responsibilities

| Function | Activities |
|---|---|
| Study physician | Perform medical history taking, review existing medical documents (if any), and perform physical examination of the patient. |

## 3. Procedures

1) When a patient is proposed by the care provider for consideration for enrollment, the investigator will visit the patient taking along the CRF and consent forms.

   Age of the patient and duration of the digestive disorders (see definitions below) will be assessed. Children aged below 1 year, or children and adults (aged ≥1 year) without persistent diarrhoea and without persistent abdominal pain, or adults (≥18 years) with persistent abdominal pain only (but *without* persistent diarrhoea) will not be enrolled.

2) A quick clinical assessment of the patient will be performed. Those in need of immediate intensive or surgical care will not be included in the study and the investigator will communicate this to the care provider.

3) The clinical assessment will include direct observation of the conjunctivae to exclude any case of clinical jaundice (to avoid including patients with viral hepatitis).

4) Any patient already participating in other ongoing diagnostic studies and/or clinical trials will not be enrolled in the study.

5) A written informed consent will be obtained from the patient before enrollment in the study (see procedure in SOP-WP6-DOC-01-V1-01Feb2012); patients who are not willing or physically or mentally not able to give consent will not be included in the study.

   - If the patient is an adult (≥18 years), he/she can give consent directly.
   - If the patient is an adolescent between 12 and 18 years, take the consent from his/her parents/guardians. Also take the assent from the adolescent.
   - If the patient is < 12 years, take the consent from parent/guardian.

6) Complete the *checklist for patient inclusion* on page 4 in the CRF:

| INCLUSION / EXCLUSION CRITERIA (to be filled by study physicians) | | | |
|---|---|---|---|
| **INCLUSION CRITERIA:** | | YES | NO |
| 1 | Adult (aged ≥18 years) presenting with persistent diarrhoea (≥2 weeks) **AND/OR** | ☐ | ☐ |
| 2 | Child (aged 1-18 years) presenting with persistent diarrhoea (≥2 weeks) **AND/OR** | ☐ | ☐ |
| 3 | Child (aged 1-18 years) with persistent abdominal pain (≥2 weeks) | ☐ | ☐ |
| **EXCLUSION CRITERIA** | | YES | NO |
| 1 | Child <1 year | ☐ | ☐ |
| 2 | Patient in need of immediate intensive or surgical care | ☐ | ☐ |
| 3 | Patient unwilling or unable to comply with study requirements | ☐ | ☐ |
| 4 | Patient unwilling or unable to provide the informed consent | ☐ | ☐ |
| 5 | Patient presenting with clinical jaundice | ☐ | ☐ |
| 6 | Patient participating in other ongoing diagnostic studies and/or clinical trials | ☐ | ☐ |
| **SELECTION FOR THIS STUDY** | | YES* | NO** |
| The patient meets one of the inclusion criteria and none of the exclusion criteria | | ☐ | ☐ |
| *** If YES, the study physician assigns the following PATIENT No:** ☐☐☐☐☐- ☐☐ | | | |
| **** If NO, the patient cannot be included in the study!** | | | |

## 4. Definitions

CRF = Case Report Form

Persistent diarrhoea: *WHO definitions are used. The WHO defines <u>diarrhoea</u> as the passing of three or more loose stools (which take the shape of the container) within a 24 hour period. A new episode of diarrhoea can occur after two full days without diarrhoea. Episodes of diarrhoea lasting for less than 14 days are defined as acute, episodes lasting for more than 14 days are defined as <u>persistent</u>. (<u>http://www.who.int/mediacentre/factsheets/fs330/en/index.html</u>)*

Persistent abdomial pain: *No official WHO definition exists. Here, we define <u>persistent abdominal pain</u> as localized or diffuse abdominal pain lasting for more than 14 days (possibly with intermittence/ recurrence)*

## 5. Records and archives

| **Appendices & Forms for completion** | |
|---|---|
| **Number** | **Title** |
| | Case Report Form (CRF) |

## 6. Document History

| Revision | |
|---|---|
| SOP-WP2-CLIN-15- V1.0-12Dec2013 | First final version by Moussa Sacko & Renion Sayé |
| SOP-WP2-CLIN-15-V1.0-02.04.2014 | Revision by Katja Polman and other participants of the Asian training workshop |
| SOP-WP2-CLIN-15-V1.0-01.05.2014 | Revision by Sören L. Becker |
| SOP-WP2-CLIN-15-V1.0-19.05.2014 | Approval by Céline Schurmans |

| Name and function | Date | Signature |
|---|---|---|
| *Author* | | |
| Ninon Horié | 12.12.2013 | |
| *Reviewed by* | | |
| Katja Polman (together with Fransiska Meyanti (Indonesia), Basudha Khanal (Nepal) and further participants of the regional Asian training workshop) | 01.04.2014 | |
| Sören L. Becker | 01.05.2014 | |
| *Approved by* | | |
| Céline Schurmans | 21.05.2014 | |
