# Supplementary material for: Experiences and Lessons from a Multicountry NIDIAG Study on Persistent Digestive Disorders in the Tropics
Source: PLoS Negl Trop Dis. 2016 Nov 3;10(11):e0004818. doi: 10.1371/journal.pntd.0004818 (PMC5094778; doi:10.1371/journal.pntd.0004818)
Supplement: S6 Clinical SOP — (PDF) [file pntd.0004818.s006.pdf]

	**SOP title:** Patient recruitment and patient flow
	**Study title**: Diagnosis of neglected tropical diseases (NTDs) in patients presenting with persistent digestive disorders (≥2 weeks) in Côte d'Ivoire, Indonesia, Mali and Nepal.

1. Scope and application

This SOP describes how patients are recruited in the NIDIAG study on persistent digestive disorders. Additionally, the patient flow and patient work-up are described in a flow chart.

2. Responsibilities

**Function**	**Activities**
Study physician	• Obtain the informed consent from eligible patients.<br>• Perform clinical history taking and physical examination.<br>• Provide clinical diagnosis.
Laboratory technician	• Document incoming samples in the "Study Specimen Log".<br>• Perform laboratory tests on stool and urine samples.<br>• Document the results of laboratory tests in the lab result form.
Study site investigator/Study nurse	• Check eligible patients for potential inclusion in the study.<br>• Document eligible patients in the "Patient Identification List".<br>• Help with CRF filling/Transcribe results from lab result form to the CRF.<br>• Check all study documents for completeness, accuracy and consistency.<br>• Provide sampling containers to study participants and explain how to obtain stool and urine samples.

3. Procedures

- Patients are recruited in the respective study centers in each study country (Côte d'Ivoire, Indonesia, Mali and Nepal). If the amount of patients recruited in the hospital setting is too low, the study team may decide to collaborate with Peripheral Health Centers (PHCs) in the surroundings of the study center to increase the number of patients with persistent digestive disorders.
- For the recruitment of asymptomatic controls, please refer to the respective SOP (SOP-WP2-CLIN-13).
- Once an eligible patient presents to one of the study centers, follow this study flowchart for patient work-up:

Patient with persistent digestive disorders (as defined in the SOP on assessment of inclusion and exclusion criteria, SOP-WP2-CLIN-15) presents to the study site

The study investigator registers the patient in the **Patient Identification List**

The study site investigator checks the inclusion/exclusion criteria

Site investigator informs patient about the study and provides the informed consent form

Patient will not be included in the study

Patient will be followed according to the local standard of care

Patient consents to study participation

Patient refuses to participate in the NIDIAG study

1) Assign a unique study number to the patient and document it in the Patient Identification List (according to SOP on numbering system, SOP-WP6-DOC-02)
2) Obtain patient signature on the informed consent sheet
3) Study physician performs clinical examination and study physician/site investigator fills in the CRF
4) Document "syndromic diagnosis" in the CRF

- Provide a pre-labelled stool sampling container to the patient and instruct the patient according to the SOP entitled "How to obtain a stool sample" (SOP-WP2-LAB-56)
- In Côte d'Ivoire and Mali, also provide a urine sampling container to the patient (SOP-WP2-LAB-36)
- Ask the patient to provide fresh samples as soon as possible (ideally on the same day or the next day in the morning)

Laboratory technician to fill in the **Study Specimen Log**

Processing of stool (and urine, if applicable) samples according to SOP on diagnostic sample flow (SOP-WP2-LAB-62)

- Document the results of laboratory tests in the local lab result form
- Study investigator transcribes the results from the lab result form into the CRF
- On the day of sampling, document "laboratory diagnosis", "provisional clinical diagnosis" and "clinical treatment decision" in the CRF

- Perform follow-up assessment after 3-5 days according to instructions in the CRF
- Document complete laboratory results from the first stool sample in the CRF
- If necessary, obtain second stool sample
- Re-evaluate treatment and modify if necessary (according to national guidelines or SOP on specific treatment procedures, see SOP-WP2-CLIN-14)

Persistent digestive disorders resolved?

YES

Document final clinical diagnosis and check all study documents for completeness

NO

Perform further follow-up visit after 1-2 weeks according to instructions in the CRF

4. Records and archives

**Appendices & Forms for completion**	
**Number**	**Title**
	Case Report Form (CRF)
	Patient Identification List
	Study Specimens Log
	Informed Consent Form
	Laboratory Result Form
SOP-WP2-LAB-56	SOP “How to obtain a stool sample”
SOP-WP2-LAB-36	SOP “How to obtain a urine sample”
SOP-WP2-LAB-62	SOP “Diagnostic sample flow”
SOP-WP2-CLIN-15	SOP “Assessment of inclusion and exclusion criteria”
SOP-WP2-CLIN-13	SOP “Selection of asymptomatic controls”
SOP-WP6-DOC-02	SOP “Numbering system to be used in NIDIAG WP2 studies”

5. Document history

**Version**	
SOP-WP2-CLIN-16-V1.0-9Jun2014	Initial version by Soeren Becker
SOP-WP2-CLIN-16-V1.0-10Jun2014	Revision by Peiling Yap and Harry van Loen
SOP-WP2-CLIN-16-V1.0-13Jun2014	Approval by Saye Renion and Silué D. Kigbafori

**Name and function**	**Date**	**Signature**
Author		
Sören Becker	09.06.2014	
Revision		
Peiling Yap<br>Harry van Loen	10.06.2014	
Approval		
Joel Azragnon	13.06.2014	
