# Supplementary material for: Experiences and Lessons from a Multicountry NIDIAG Study on Persistent Digestive Disorders in the Tropics
Source: PLoS Negl Trop Dis. 2016 Nov 3;10(11):e0004818. doi: 10.1371/journal.pntd.0004818 (PMC5094778; doi:10.1371/journal.pntd.0004818)
Supplement: S2 Laboratory SOP — (PDF) [file pntd.0004818.s008.pdf]

nidiag – Better Diagnosis of Neglected Infectious Diseases	**SOP title: Modified acid-fast (Ziehl-Neelsen) staining technique**
	**Study title**: Diagnosis of neglected tropical diseases (NTDs) in patients presenting with persistent digestive disorders (≥2 weeks) in Côte d'Ivoire, Indonesia, Mali and Nepal

1. Scope and Application

The Ziehl-Neelsen staining technique enables detection of pathogenic coccidian intestinal protozoa in human stool specimens. The three important coccidians that may cause persistent digestive disorders are *Cryptosporidium* spp., *Cyclospora cayetanensis* and *Cystoisospora belli*. The principle behind the ZN technique is that it allows for detection of so-called "acid-fast organisms" such as coccidian intestinal protozoa and mycobacteria. Once these organisms are stained with a specific dye, they are difficult to decolorize, and retain a red color even when treated with a mixture of acid and alcohol (while all other structures present in the sample will decolorize). Methylene blue is used as counter stain, hence only the acid-fast organisms will appear as red-colored and can thus easily be detected in a stool sample.

2. Responsibilities

Function	Activities
Laboratory Technician	▪ Perform the Kinyoun technique blinded to the results of other relevant diagnostic tests (e.g. Cryptosporidium rapid diagnostic test).<br>▪ Report the results in the Hospital Lab Register.
Study Nurse/Study Assistant	▪ Transcribe the results from the Hospital Lab Register to the Case Report Form (CRF).

3. Procedures

3.1 Safety and storage of reagents

- All faecal samples are potentially infectious. Wear gloves during the procedure.
- At each study site, safety precautions for handling and disposal of infectious materials should be practiced according to the laboratory safety rules of the participating hospital.
- ***Fuchsin base (ZN A):*** Harmful in contact with skin and if swallowed. Irritating to eyes and skin. Possible risks of irreversible effects. Keep container tightly closed. Keep away from sources of ignition - No Smoking. In case of contact with eyes, rinse immediately with plenty of water and seek medical advice. Wear suitable protective clothing and gloves.
- ***Decolorizer (ZN B):*** Harmful by inhalation and if swallowed. Keep container tightly closed and in a well ventilated place. Keep away from sources of ignition - No smoking. Wear suitable protective clothing.
- ***Methylene Blue (ZN C):*** Harmful by inhalation and if swallowed. Irritating to eyes, respiratory system and skin. Possible risks of irreversible effects. Keep container tightly closed. Keep away from sources of ignition - No Smoking. Wear suitable protective clothing and gloves.
- **Storage of the reagents**: Store TB Stain Kits and Reagents at 15 - 30ºC. Reagents that have been removed from the packing carton should be stored in the dark. The expiration date applies to the product in its intact container when stored as directed.

3.2.1 Materials required

- ZN A (Fuchsin base)
- ZN B (Decolorizer)
- ZN C (Methylene Blue)
- Staining rack
- Tap water
- Bunsen burner (or other source of flame)
- Microscope slide
- Light microscope with oil immersion
- Stool containing cysts of one coccidian protozoan species (for quality control)

3.2.2 Specimen collection and preparation

- Apply a thin smear of the fresh stool specimen directly on a clean microscope slide.
- Allow smear to dry.
- Fix the smear to the slide by passing the slide through a low flame 2 - 3 times, avoiding excessive heat.
- Quality control: For quality control of the Kinyoun stain, a positive control (e.g. containing *Cryptosporidium* oocysts) should always be included when the staining procedure is performed.

3.3 Staining procedure and analysis of microscope slides

1. Place slides on a staining rack and flood with fuchsin base (ZN A) for 4 min. Do not heat.
2. Wash gently in running water.
3. Decolorize with Decolorizer (ZN B) for 3 - 5 s.
4. Wash gently in running water.
5. Counterstain with Methylene blue (ZN C) for 30 s.
6. Wash gently in running water.
7. Air dry.
8. Examine the microscope slide carefully using a light microscope with 400x and 1000x magnification. Use oil immersion for the 1000x magnification. Acid-fast pathogens will stain reddish-pink and can easily be distinguished.
    - Note: Examine the stained slides preferably on the same day. If this is not possible, the slides can be examined later.
9. Differentiate different intestinal protozoa based on their different characteristics (see Figure 1).
    - Note: Also elements other than coccidian intestinal protozoa may stain in a similar way (e.g. some yeasts, helminth eggs, microsporidia). Staining artifacts may appear pinkish-red and might occasionally be confused as intestinal protozoa. Try always to identify and differentiate the sought intestinal protozoa based on their morphology, their size and their internal structures (see Figure 1). In uncertain cases, seek advice from an expert microscopist.

Cryptosporidium spp.	*Cyclospora cayetanensis*	*(Cysto-)Isospora belli*
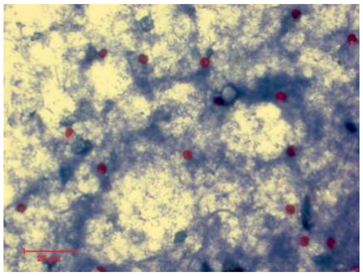<br>Image: S. Becker	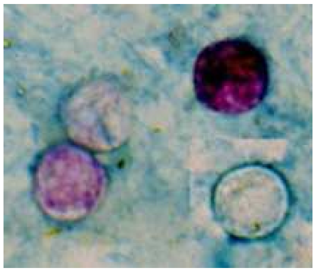<br>Image: CDC (DPDx)	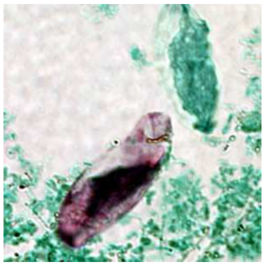<br>Image: CDC (DPDx)
- Spherical form<br>- Size of oocysts: 4-6 µm<br>- Stain bright pinkish-red	- Round, spherical form<br>- Size of oocysts: 8-10 µm<br>- Variably acid-fast (may range from unstained to pink to deep red)<br>- Note: As Methylene blue is used as counterstain in the modified acid-fast technique, the background colour will be blue (instead of green as in the picture).	- Ellipsoidal shape<br>- Size: 20-30 µm long, 10-20 µm wide<br>- Up to two round sporocysts may be seen inside the mature parasite<br>- Note: As Methylene blue is used as counterstain in the modified acid-fast technique, the background colour will be blue (instead of green as in the picture).

Figure 1 Differences between the three important coccidian pathogens giving rise to persistent digestive disorders.

3.4 Documentation of results

- Results should be recorded in the hospital lab register and later transcribed to the CRF.
  - Record if the test was done or not, and if the test was not done, provide a reason for not doing it.
  - Record if the result is POSITIVE or NEGATIVE.
  - If POSITIVE, record the detected intestinal protozoa species.
  - If POSITIVE, record the number of intestinal protozoa (for each species) and use the following semi-quantitative scheme:
    - Negative (no parasites seen);
    - + (≤1 parasite per 10 observation fields of 1000x magnification);
    - ++ (1-10 parasites per 10 observation fields of 1000x magnification);
    - +++ (1-10 parasites per observation field of 1000x magnification); and
    - ++++ (>10 parasites per observation field of 1000x magnification).

3.5 Preservation of microscope slides

- For quality control purposes, keep all stained slides with a unique identifier in a microscope slide box in a dry and cool place.

3.6 Waste management

- Dispose remaining stool samples as biohazards without contaminating the local environment.

4. REFERENCE

- Becker SL, Vogt J, Knopp S, Panning M, Warhurst DC, Polman K, Marti H, von Muller L, Yansouni CP, Jacobs J, Bottieau E, Sacko M, Rijal S, Meyanti F, Miles MA, Boelaert M, Lutumba P, van Lieshout L, N'Goran EK, Chappuis F, Utzinger J, 2013. Persistent digestive

disorders in the tropics: causative infectious pathogens and reference diagnostic tests. *BMC Infect Dis* 13:37

5. Records and archives

**Appendices & Forms for completion**	
**Number**	**Title**
1	Hospital Lab Register
2	CRF

6. Document History

**Revision**	
SOP-WP2-LAB-51-V01-17Feb2014	Initial version by Sören Becker
SOP-WP2-LAB-51-V02-20Feb2014	Reviewed by Lisette van Lieshout
SOP-WP2-LAB-51-V03-23 Feb2014	Approved by Lutz von Müller
SOP-WP2-LAB-51-V3.2-21May2014	Country-specific adaptation by Elsa Murhandarwati for the NIDIAG study sites in Indonesia
SOP-WP2-LAB-51-V3.2-24Jun2014	Approval of the adapted version for Indonesia by Sören Becker

**Name and function**	**Date**	**Signature**
Author		
Elsa Murhandarwati	21.05.2014	
Reviewed by		
Sören Becker	23.06.2014	
Approved by		
Sören Becker	24.06.2014	
