# Supplementary material for: Experiences and Lessons from a Multicountry NIDIAG Study on Persistent Digestive Disorders in the Tropics
Source: PLoS Negl Trop Dis. 2016 Nov 3;10(11):e0004818. doi: 10.1371/journal.pntd.0004818 (PMC5094778; doi:10.1371/journal.pntd.0004818)
Supplement: S3 Laboratory SOP — (PDF) [file pntd.0004818.s009.pdf]

	**SOP Title:** Crypto-Giardia Duo-Strip Rapid Diagnostic Test
	**Study title**: Diagnosis of neglected tropical diseases (NTDs) in patients presenting with persistent digestive disorders (≥2 weeks) in Côte d'Ivoire, Indonesia, Mali and Nepal.

1. Scope and application

This Rapid Diagnostic Test (RDT) is ready to use and is based on the homogeneous membrane system technology with colloidal gold particles. This device allows detection of both *Giardia lamblia* (syn.: *Giardia intestinalis*) and *Cryptosporidium* spp. in stool specimens. This device consists of a two-sided stick (similar to two separated sticks which would be placed back to back) each side specific either for *Cryptosporidium* or for *Giardia lamblia*. This SOP is applicable for the diagnostic evaluation of *Cryptosporidium* spp. and *Giardia lamblia* in patients enrolled under the digestive syndrome of the NIDIAG study in Côte d'Ivoire, Indonesia, Mali and Nepal.

2. Responsibilities

Function	Activities
Laboratory Technician	▪ Perform the Crypto-Giardia Duo-Strip rapid diagnostic test.<br>▪ Report the results in the Hospital Lab Register.
Study Nurse/Study Assistant	▪ Transcribe the results from the Hospital Lab Register to the Lab Report Form of the Case Report Form (CRF).

3. Safety, material and samples

3.1. Safety

- Handle all samples as potentially infectious. Wear gloves during the procedure.
- At each study site, safety precautions for handling and disposal of infectious materials should be practiced according to the laboratory safety rules of the participating hospital.

3.2. Materials and samples

3.2.1. Materials required

- Crypto/Giardia Duo-Strip (article no. C-1018, Coris BioConcept, Gembloux, Belgium). These strips come in a bottle or in a pouch with a desiccant bag.
- Dilution Buffer (15mL). Saline solution buffered to pH 7.5 with Tris and containing EDTA, NaN_3 (>0.1%), a detergent and blocking proteins.
- 3 or 5 mL test tubes
- Sampling loops for taking the faecal samples
- Cardboard rack

3.2.2. Samples

- Fresh stool (please refer to the SOP for stool collection)
- Comment: Stool samples should be analyzed on the day of collection.

4. Procedures

4.1. Preparations of the test

- Allow kit components, in unopened packaging, and specimens to reach room temperature (15-30°C) before performing a test.
- Once opened, run the test immediately. Indicate the patient's name or specimen number on the tube. Place the marked test tubes in a rack.

4.2. Specimen preparation procedure

- Add 0.5mL or 15 drops of the dilution buffer solution into a tube.
- Dip a loop containing the stool sample into the tube. **The dilution ratio must be about 4% w/v.** For liquid samples, take 2 loops of 10 µl; for solid samples, take 1 loop.
- Discard the sampling loop into the provided disposal
- Vortex the preparation to homogenize until the entire stool sample is suspended.
- Dip the sensitized strip in the direction indicated by the red arrow.
- Leave to react for 15 minutes.
- Read the result after 15 minutes.
- Positive results may be reported sooner (in case both the test and control lines become visible before 15 minutes).
- **Do not take the appearance of new lines into account after the reaction time is passed. The results must be read on still wet strips.**
- **Repeat the procedures when the kit shows an invalid result. Any test in which no control line appears within 15 minutes has to be considered invalid and the results must not be used.**

- **Procedure summary:**

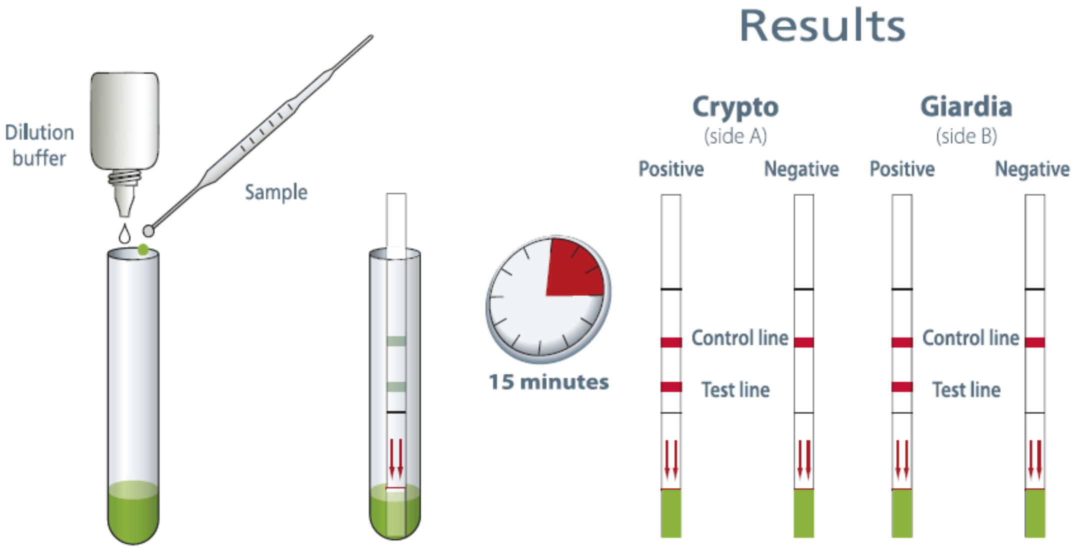

4.3. Documentation of results

- Record the results in the Hospital Laboratory Register.
- Record if the test was done or not, and provide a reason if it was not done.
- Record if the result is POSITIVE or NEGATIVE.
- In case of an invalid test result, repeat the RDT.
- Record the line intensity of the test line in the CRF (e.g. trace, 1+ (clearly positive, but faint line), 2+ (clearly positive with strong line), 3+ (strongly positive with very strong line)
- Transcribe the results into the CRF.

4.4. Waste management

- Dispose remaining stool samples according to the safety precautions of the local laboratory without contaminating the local environment.

5. References

Johnston S.P. et al. 2003. *Evaluation of three commercial assays for detection of giardia and cryptosporidium organisms in fecal specimens.* Journal of Clinical Microbiology, p.623-626

6. Records and archives

**Appendices & Forms for completion**	
**Number**	**Title**
1	Hospital Lab Register
2	CRF

7. Document History

**Revision**	
SOP-WP2-LAB-52-V01-19Nov2013	Initial version
SOP-WP2-LAB-52-V02-23Feb2014	Revised version after review by Elsa Murhandarwati, Katja Polman, Basudha Khanal, Sören L. Becker
SOP-WP2-LAB-52-V03-11Mar2014	Approved by Pascal Mertens
SOP-WP2-LAB-52-V04-14Jun2014	Adaptation of the semi-quantitative measurement in the section “4.3 Documentation of results”

**Name and function**	**Date**	**Signature**
Author		
Pierre Schneeberger	19.11.2013	
Reviewed by		
Elsa Murhandarwati<br>Katja Polman<br>Basudha Khanal<br>Sören L. Becker	06.12.2013<br>09.12.2013<br>15.12.2013<br>23.02.2014	
Approved by		
Ninon Horié	07.07.2014	
