# Supplementary material for: Experiences and Lessons from a Multicountry NIDIAG Study on Persistent Digestive Disorders in the Tropics
Source: PLoS Negl Trop Dis. 2016 Nov 3;10(11):e0004818. doi: 10.1371/journal.pntd.0004818 (PMC5094778; doi:10.1371/journal.pntd.0004818)
Supplement: S4 Laboratory SOP — (PDF) [file pntd.0004818.s010.pdf]

nidiag – Better Diagnosis of Neglected Infectious Diseases	**SOP Title:** Kato-Katz technique
	**Study title**: Diagnosis of neglected tropical diseases (NTDs) in patients presenting with persistent digestive disorders (≥2 weeks) in Côte d'Ivoire, Indonesia, Mali and Nepal.

1. Scope and application

The Kato-Katz technique facilitates the detection and quantification of helminth eggs that infected subjects pass in their faeces. A thick smear is prepared on a microscopic slide and helminth eggs are enumerated under a light microscope and recorded for each helminth species separately. Subsequently, the prevalence and intensity of helminth infections can be determined. This SOP is applicable for the diagnostic evaluation of *Schistosoma mansoni* (in Africa), the three common soil-transmitted helminths (*Ascaris lumbricoides*, *Trichuris trichiura* and hookworm), *Taenia* spp. and other helminths in patients enrolled under the digestive syndrome of the NIDIAG study in Côte d'Ivoire, Indonesia, Mali and Nepal.

2. Responsibilities

Function	Activities
Laboratory Technician	▪ Perform the Kato-Katz technique blinded to the results of the reference tests.<br>▪ Report the results in the Hospital Lab Register.
Study Nurse/Study Assistant	▪ Transcribe the results from the Hospital Lab Register to the Case Report Form (CRF).

3. Procedures

3.1 Safety

- Handle all faecal samples as potentially infectious. Wear gloves during the procedure.
- At each study site, safety precautions for handling and disposal of infectious materials should be practiced according to the laboratory safety rules of the participating hospital.

3.2 Materials and samples

3.2.1 Materials required

- Standard Kato-Katz plastic template with a punched hole for 41.7 milligrams (mg) of stool
- Aluminum foil
- Wire/plastic mesh
- Small plastic spatula
- Cellophane, pre-soaked in methylene blue for at least 24 hours (h)
- Microscope slide
- Light microscope
- Counter

3.2.2 Samples

- Fresh faecal sample (patient should hand in a fresh faecal sample; please refer to the SOP for stool collection).
- Comment: Faecal samples should be analyzed on the day of stool production and collection.

3.3 Procedures

1. Place a standard Kato-Katz template on a microscope slide, which has been labeled with a NIDIAG patient number.
2. Scoop approximately 2-3 grams (g) of a fresh faecal sample onto a piece of aluminum foil, and press a piece of wire or plastic mesh on top to sieve it.
3. Using a small plastic spatula, scrape the sieved material off the mesh and completely fill the hole in the Kato-Katz template. To remove excess faecal material, level the content of the hole with the spatula.
4. Vertically remove the template without disturbing the faecal material now adhering to the microscope slide. The template and spatula can be cleaned in water with detergent, rinsed in clean water, and reused.
5. Place a piece of pre-soaked cellophane over the faecal sample on the microscope slide.
6. To spread the faecal material into a thick smear, gently press a clean microscope slide against the sample slide, evenly distributing the material within a circle of a diameter slightly smaller than the width of the microscopic slide.
7. Allow the slide to clear for 30-60 minutes (min), during which the slides must be kept away from direct sunlight. When hookworm is present in the community under investigation (in all 4 study countries), it is essential to read the slides shortly after a clearing time of 30 min, with a maximum clearing time of 60 min. In Côte d'Ivoire and Mali, examine each slide again within the next 12-24 hours for detection of *Schistosoma mansoni* eggs. Examine the thick smear under a light microscope (40-100x magnification). Count the number of helminth eggs and record them for each helminth species separately.
8. To enhance the sensitivity of this technique, two Kato-Katz thick smears have to be prepared in parallel for each faecal sample. Whenever only one single helminth egg is detected on a Kato-Katz thick smear, this has to be confirmed by a second laboratory technician.

3.4 Documentation of results

- The following results should be recorded in the CRF:
  - Record if the test was done or not, and if the test was not done, provide a reason for not doing it.
  - Record if the result is POSITIVE or NEGATIVE.
  - If POSITIVE, record the number of helminth eggs observed on the slide according to the species of the detected organisms.
  - To get the number of eggs per 1 g of stool (EPG), multiply the egg count from the slide by a factor of 24 (24 x 41.7 mg ≈ 1 g). For example, if you find 600 eggs of hookworm, the calculation for the number of hookworm eggs per 1 g of stool is: 600 x 24 = 14,400 EPG.

3.5 Waste management

- Dispose remaining stool samples and slides with faecal thick smears without contaminating the local environment.

4. References

- WHO, 1994. Bench aids for the diagnosis of intestinal parasites. Geneva: World Health Organization
- Yap P, Fürst T, Müller I, Kriemler S, Utzinger J, Steinmann P, 2012. Determining soil-transmitted helminth infection and physical fitness of school-aged children. *Journal of Visualized Experiments* (66): e3966

5. Records and archives

**Appendices & Forms for completion**	
**Number**	**Title**
1	Hospital Lab Register
2	CRF

6. Document History

**Revision**	
SOP-WP2-LAB-53-V01-18Nov2013	Initial version
SOP-WP2-LAB-53-V02-05Dec2013	Reviewed by Sören L. Becker
SOP-WP2-LAB-53-V03-09Dec2013	Reviewed by Elsa Murhandarwati, Suman Rijal and Katja Polman
SOP-WP2-LAB-53-V04-13Dec2013	Revised by Peiling Yap
SOP-WP2-LAB-53-V05-13Feb2014	Revised by Jürg Utzinger

**Name and function**	**Date**	**Signature**
Author		
Peiling Yap	19.10.2013	
Reviewed by		
Sören L. Becker	05.12.2013	
Elsa Murhandarwati	06.12.2013	
Katja Polman	09.12.2013	
Suman Rijal	09.12.2013	
Moussa Sacko	11.12.2013	
Peiling Yap	13.12.2013	
Jürg Utzinger	13.02.2014	
Approved by		
Jürg Utzinger	13.02.2014	
