# Supplementary material for: Experiences and Lessons from a Multicountry NIDIAG Study on Persistent Digestive Disorders in the Tropics
Source: PLoS Negl Trop Dis. 2016 Nov 3;10(11):e0004818. doi: 10.1371/journal.pntd.0004818 (PMC5094778; doi:10.1371/journal.pntd.0004818)
Supplement: S5 Laboratory SOP — (PDF) [file pntd.0004818.s011.pdf]

	**SOP Title:** Baermann technique
	**Study title**: Diagnosis of neglected tropical diseases (NTDs) in patients presenting with persistent digestive disorders (≥2 weeks) in Côte d'Ivoire, Indonesia, Mali and Nepal.

1. Scope and application

The Baermann technique enables detection of the larvae of *Strongyloides stercoralis* and other pathogens, such as hookworm and *Trichostrongylus* spp. in the faeces of patients enrolled under the digestive syndrome of the NIDIAG study in Côte d'Ivoire, Indonesia, Mali and Nepal. The principle behind the Baermann technique is that since the larvae are phototactic and motile, they will migrate out of an illuminated fresh faecal sample submerged in tap water to be collected and observed under a light microscope.

2. Responsibilities

Function	Activities
Laboratory Technician	- Perform the Baermann technique blinded to the results of the reference tests.<br>- Report the results in the Hospital Lab Register.
Study Nurse/Study Assistant	- Transcribe the results from the Hospital Lab Register to the Case Report Form (CRF).

3. Procedures

3.1 Safety

- All faecal samples are potentially infectious. Wear gloves during the procedure.
- At each study site, safety precautions for handling and disposal of infectious materials should be practiced according to the laboratory safety rules of the participating hospital.

3.2 Materials and samples

3.2.1 Materials required

- Glass funnel that can hold at least 50 milliliters (ml) of water
- Rubber tube
- Clip
- Sieve or a piece of wire screen shaped like a cone
- Medical gauze
- Artificial light
- Centrifuge
- Centrifuge tube [50 millimeters (ml)]
- Tap water
- Microscopic slide
- Cover slip
- Light microscope

3.2.2 Samples

- Fresh faecal samples (patient should hand a fresh faecal sample; please refer to the SOP for stool collection).

- Comment: Stool samples should be analyzed on the day of production and collection.

3.3 Procedures

1. Set up a stand that can hold a glass funnel and attach a rubber tube, closed with a clip, to the bottom of the funnel (see Figure 1).
2. Place a sieve or a piece of wire screen shaped like a cone, in the funnel and layer it with a piece of medical gauze.
3. Fill up the funnel with tap water and ensure that the whole set-up does not leak.
4. Scoop 20-30 gram (g) of fresh stool onto the middle of the medical gauze and ensure that it is completely submerged in water. Add more water if necessary. Fold the medical gauze over the sample.
5. From below, shine artificial light at the funnel for 3 hours (hr).
6. Drain 50 ml of the lower portion of the water in the funnel into a centrifuge tube by gently releasing the clip on the rubber tube to avoid splashing of water and overfilling of centrifuge tube.
7. Centrifuge the collected water at 600 x *g* (relative centrifugal force) for 5 minutes (min) and carefully pour out the supernatant without disturbing the sediment. Retain the last few drops in the tube.
8. Re-suspend the sediment and pipette 2 drops of the solution onto a microscope slide and examine the slide under a light microscope (40x magnification for detection and 100-400x magnification for species confirmation).

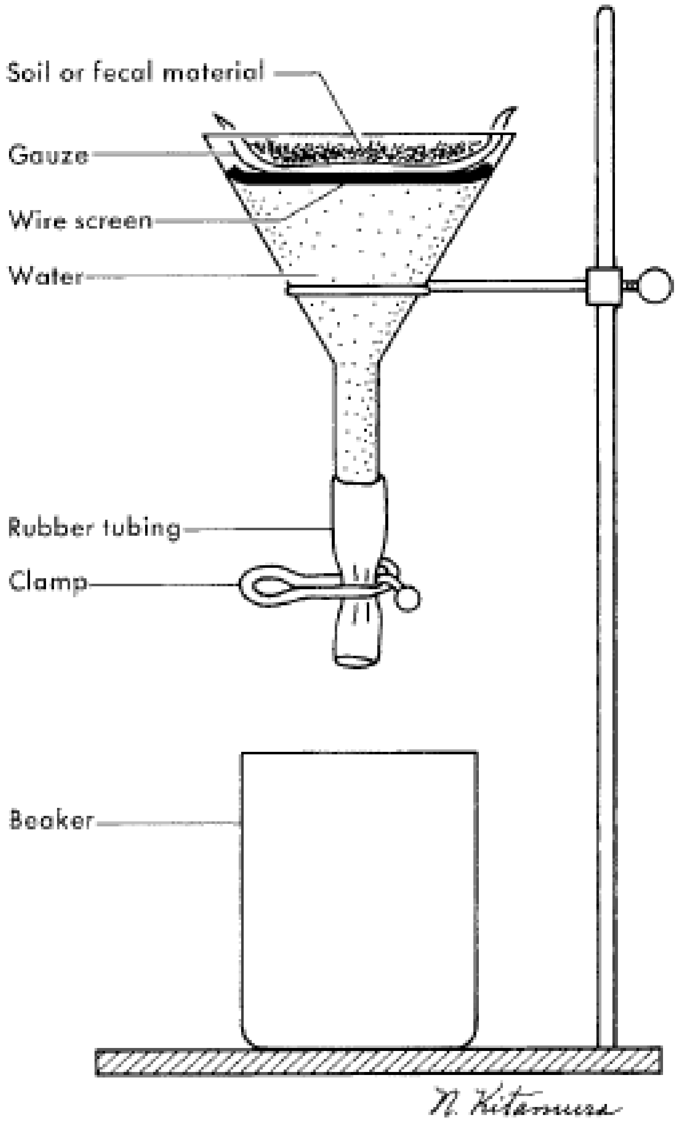

Figure 1 Set up for the Baermann technique

3.4 Documentation of results

- Results should be recorded in the hospital lab register and later transcribed to the CRF.
  - Record if the test was done or not, and if the test was not done, provide a reason for not doing it.
  - Record if the result is POSITIVE or NEGATIVE.
  - If POSITIVE, record the species of larvae detected and the number of larvae of each species observed on the microscope slide (see Figure 2 for differences between hookworm and *Strongyloides* L_1 larvae).

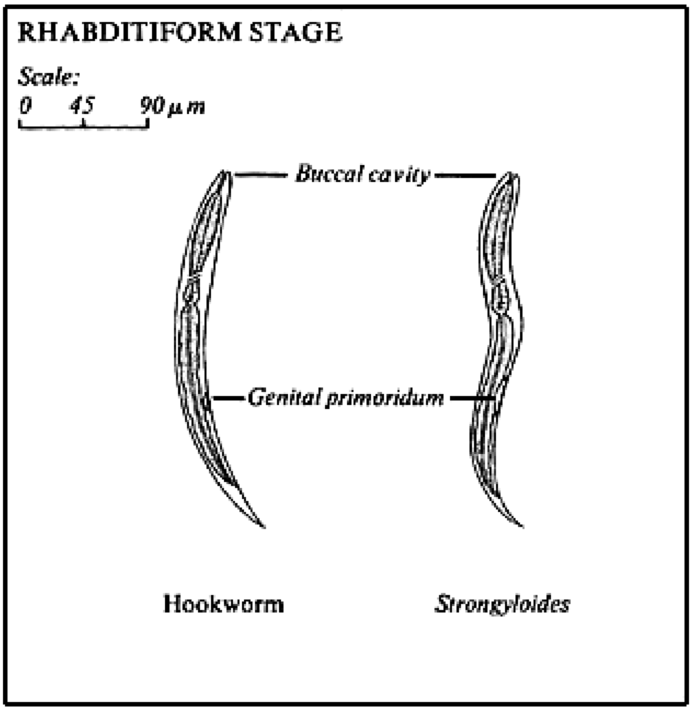

Figure 2 Differences between hookworm and *Strongyloides* L_1 larvae (*Strongyloides* has a shorter buccal cavity and larger genital primoridum than hookworm)

3.5 Preservation of Baermann sediments

- Preservation of Baermann sediments should be done for microscopically positive sediments. Mix 1 ml of the positive sediment with 1 ml of ethanol (if available 96%) in a Greiner 146361 tube. Transfer the aliquots to a freezer as soon as possible. If possible, store the aliquots -80°C. If this is not possible in your study site, store the sample at -20°C. If no freezer is available at all at the study sites, store the samples in the refrigerator.

3.6 Waste management

- Dispose remaining stool samples, the medical gauze containing stool samples and water left in the funnel as biohazards without contaminating the local environment.
- Glass funnel and sieve or wire screen can be washed with hot water without detergent and re-used.

4. References

- García LS, 2007. Diagnostic medical parasitology. Washington, DC: ASM Press, pp. 1-1202.
- Yap P, Fürst T, Müller I, Kriemler S, Utzinger J, Steinmann P, 2012. Determining soil-transmitted helminth infection and physical fitness of school-aged children. *Journal of Visualized Experiments* (66): e3966

5. Records and archives

**Appendices & Forms for completion**	
**Number**	**Title**
1	Hospital Lab Register
2	CRF

6. Document History

**Revision**	
SOP-WP2-LAB-54-V1-23Oct2013	Initial version
SOP-WP2-LAB-54-V2-09Dec2013	Reviewed by Katja Polman
SOP-WP2-LAB-54-V3-13Dec2013	Revised by Peiling Yap
SOP-WP2-LAB-54-V4-29Jan2014	Revised by Sören Becker and Peiling Yap
SOP-WP2-LAB-54-V4-29Jan2014	Revised by Jürg Utzinger
SOP-WP2-LAB-54-V4-4Apr2014	Revised by Katja Polman and the Asian training workshop participants
SOP-WP2-LAB-54-V5-26Apr2014	Approval by Jürg Utzinger

**Name and function**	**Date**	**Signature**
Author		
Peiling Yap	23.10.2013	
Reviewed by		
Katja Polman	09.12.2013	
Peiling Yap	13.12.2013	
Sören Becker	29.01.2014	
Peiling Yap	29.01.2014	
Jürg Utzinger	13.02.2014	
Approved by		
Jürg Utzinger	26.04.2014	
