# Supplementary material for: Experiences and Lessons from a Multicountry NIDIAG Study on Persistent Digestive Disorders in the Tropics
Source: PLoS Negl Trop Dis. 2016 Nov 3;10(11):e0004818. doi: 10.1371/journal.pntd.0004818 (PMC5094778; doi:10.1371/journal.pntd.0004818)
Supplement: S6 Laboratory SOP — (PDF) [file pntd.0004818.s012.pdf]

	**SOP Title:** Mini-FLOTAC technique
	**Study title**: Diagnosis of neglected tropical diseases (NTDs) in patients presenting with persistent digestive disorders (≥2 weeks) in Côte d'Ivoire, Indonesia, Mali and Nepal.

1. Scope and application

The Mini-FLOTAC technique facilitates the detection and semi-quantification of helminth eggs in patients recruited in the digestive syndrome of the NIDIAG study in Côte d'Ivoire, Indonesia, Mali and Nepal. The principle behind this technique is that given the correct specific gravity, helminth eggs will float and the apical layer of the floating suspension can be examined under a light microscope. Beside the high sensitivity of the technique for helminth diagnosis, the Mini-FLOTAC apparatus can be easily transferred and employed in laboratories that lack wide-bucket centrifuges.

2. Responsibilities

Function	Activities
Laboratory technician	▪ Perform the Mini-FLOTAC technique blinded to the results of the reference tests.<br>▪ Report the results in the Hospital Lab Register.
Study nurse/Study assistant	▪ Transcribe the results from the Hospital Lab Register to the Case Report Form (CRF).

3. Procedures

3.1 Safety

- Handle all samples as potentially infectious. Wear gloves and lab coat during the procedure.
- Formalin is poisonous and an irritant and should be handled under a well-ventilated hood.
- At each study site, safety precautions for handling and disposal of infectious materials should be practiced according to the laboratory safety rules of the participating hospital.

3.2 Materials and samples

3.2.1 Materials required

- Mini-FLOTAC apparatus
- Fill-FLOTAC
- 5% Formalin
- 1 mL pipette tip
- Wooden thong depressor or spatula
- Measuring cylinder
- Electronic weighing scale (accurate up to 0.1 g)
- Magnetic stirrer
- Hydrometer
- Microscope holder
- Light microscope
- Counter

3.2.2 Samples

- Fresh stool (patient should hand in the stool the following morning after container has been distributed; please refer to the SOP for stool collection).
- Comment: Stool samples should be analyzed on the day of collection (within 24 hours after receiving them in the laboratory).

3.3 Procedures

1. Assemble the Mini-FLOTAC apparatus by putting together the base with two numbered flotation chambers and the reading disk with two grids, so that the protrusion on the bottom of the disk fits into the grove at the top of the base. Hence, the disk can be turned along the grove of the base.
2. Turn the disk so that the grids of the disk are perpendicular to the chambers of the base and insert the protrusions on the bottom of the key into the holes on the right side of each chamber.
3. Use the key to turn the disk so that the grids are situated above the chambers.
4. To prepare the sample, label the Fill-FLOTAC, place it on the scale and press the tare button of the scale to set it to zero.
5. For non-diarrhoeic stool specimens, add approximately 1 g of stool to the Fill-FLOTAC using the collector of the Fill-FLOTAC device. For diarrhoeic stool specimens, pour the specimens directly into the Fill-FLOTAC and use a measuring scale to obtain approximately 2 g of stool.
6. Add 1 mL of 5% formalin to the stool specimens and homogenize with the applicator.
7. Top up mixture in Fill-FLOTAC with flotation solution (FS) to 20 mL. Type of FS used should correspond to the diagnosis that is to be performed (FS2 for soil-transmitted helminth diagnosis in all four countries; FS7 for *Schistosoma mansoni* diagnosis in Côte d'Ivoire and Mali)
   - For the diagnosis of soil-transmitted helminths, use flotation solution no. 2 (FS2; saturated sodium chloride (NaCl), specific gravity (s.g. 1.20)). FS2 is prepared as follows: add NaCl to 1 L of warm water (40–50 °C) until no more salt goes into solution (~500 g) and the excess settles on the bottom of the container. Dissolve by stirring on a magnetic stirrer. To ensure that the solution is fully saturated, it should be allowed to stand overnight at room temperature. Check the s.g. with a hydrometer, recognizing that the s.g. of the saturated solution will vary slightly depending on ambient temperature.
   - For the diagnosis of *Schistosoma mansoni*, use flotation solution no. 7 (FS7; zinc sulfate (ZnSO4·7H2O) (s.g., 1.35). FS7 is prepared as follows: add 685 g of zinc sulfate heptahydrate to 685 ml of tap water. Dissolve zinc sulfate in water by stirring on a magnetic stirrer. Check the s.g. with a hydrometer.
8. Close the Fill-FLOTAC, remove the cap of the applicator, thoroughly mix the solution by moving the applicator up and down and re-cap the applicator.
9. Tilt the Mini-FLOTAC by placing it against a 1 cm high edge and ensure that the slits in the disks are above the chambers.
10. Remove the cap of the other opening on the Fill-FLOTAC and put on a 1 mL pipette tip from which the tip has been cut off (to avoid obstruction of the pipette tip).
11. It is essential to homogenize the suspension again before each chambers is filled.
12. Turn the apparatus so that each chamber can be filled by squeezing the homogenized solution out of the Fill-FLOTAC through the pipette tip, to avoid air bubbles fill the chambers until a meniscus, i.e. small drop of solution outside of the chamber) is formed.
13. Leave to stand for 10 min at room temperature and then use the key to turn the disk so that the grids are perpendicular to the chambers.
14. Remove the key but keep the base and the disk together.
15. Place the disk and the base into the microscope holder by sliding the chambers into the gap in the middle of the holder.

16. Place the microscope holder and the Mini-FLOTAC on the microscope.
17. Systematically screen the grids for the presence of helminth eggs, count the eggs and record them for each species separately using the counter.
18. After the grids are examined the Mini-FLOTAC can be disassembled by turning the disk again so that the grids are above the chambers. Use the key to remove the disk; the entire apparatus can be washed with soapy water and re-used.

IMPORTANT: in the study centers of Côte d'Ivoire and Mali, a second Mini-FLOTAC will be performed for the diagnosis of *S. mansoni*. Repeat all steps above using FS7.

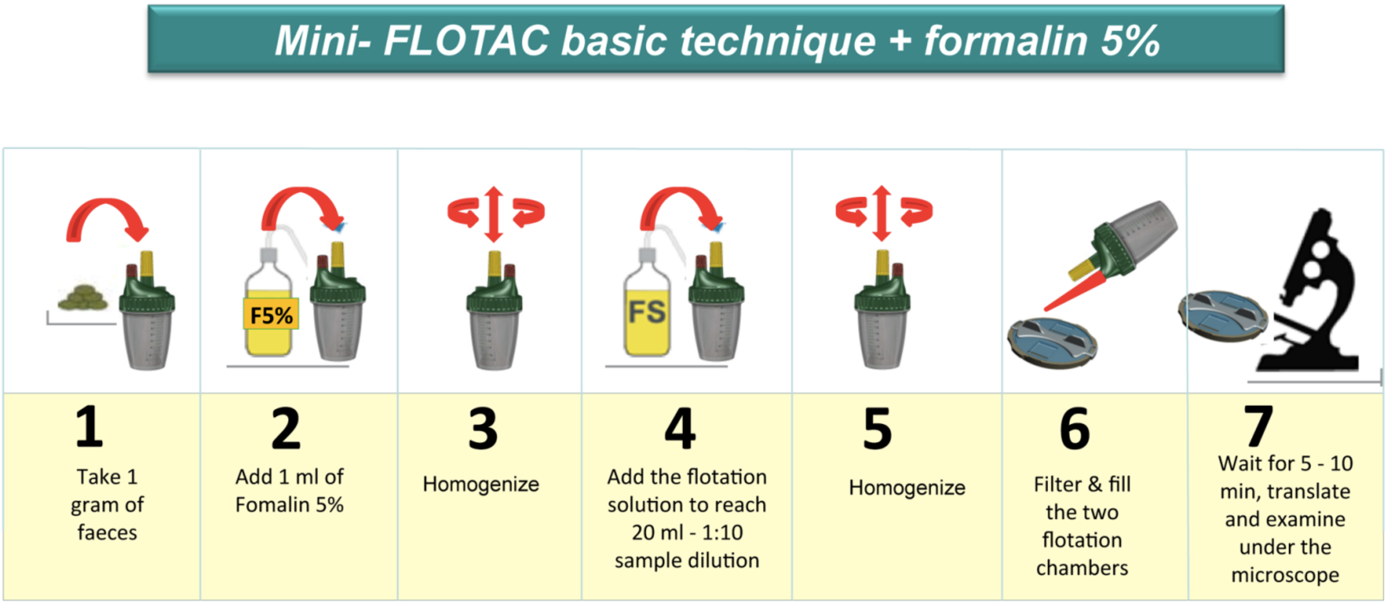

3.4 Documentation of results

- Results should be recorded in the CRF (either directly or later transcribed from hospital lab register).
- Record if the test was done or not, and if the test was not done, provide a reason for not doing it.
- Record if the result is POSITIVE or NEGATIVE.
- If POSITIVE, record the number of helminth eggs and document it for each species separately.

3.5 Waste management

- Dispose remaining stool samples and liquids without contaminating the local environment.

4. References

- Barda BD, Rinaldi L, Ianniello D, Zepherine H, Salvo F, Sadutshang T, Cringoli G, Clementi M, Albonico M, 2013. Mini-FLOTAC, an innovative direct diagnostic technique for intestinal parasitic infections: experience from the field. *PLoS Negl Trop Dis* 7**,** e2344.
- Cringoli G, Rinaldi L, Albonico M, Bergquist R, Utzinger J, 2013. Geospatial (s)tools: integration of advanced epidemiological sampling and novel diagnostics. *Geospat Health* 7, 399-404.
- Cringoli G, Rinaldi L, Maurelli MP, Utzinger J, 2010. FLOTAC: new multivalent techniques for qualitative and quantitative copromicroscopic diagnosis of parasites in animals and humans. *Nat Protoc* 5, 503-515.

5. Records and archives

**Appendices & Forms for completion**	
**Number**	**Title**
1	Hospital Lab Register
2	CRF

6. Document History

**Revision**	
SOP-WP2-LAB-55-V01-19Oct2013	Initial version
SOP-WP2-LAB-55-V02-05Dec2013	Reviewed and commented by Jean T. Coulibaly, Sören Becker and Jürg Utzinger
SOP-WP2-LAB-55-V03-09Dec2013	Reviewed by Elsa Murhandarwati and Katja Polman
SOP-WP2-LAB-55-V04-13Dec2013	Revised by Peiling Yap
SOP-WP2-LAB-55-V05-14Dec2013	Revised by Renion Sayé and Fransiska Meyanti
SOP-WP2-LAB-55-V06-14Jun2014	Adaptation of the quantitative measurement of parasitic elements, added under “3.4 Documentation of results”

**Name and function**	**Date**	**Signature**
Author		
Peiling Yap	22.10.2013	
Reviewed by		
Jean T. Coulibaly<br>Sören L. Becker<br>Jürg Utzinger<br>Elsa Murhandarwati<br>Katja Polman<br>Peiling Yap<br>Fransiska Meyanti<br>Renion Sayé	22.11.2013<br>04.12.2013<br>05.12.2013<br>06.12.2013<br>09.12.2013<br>13.12.2013<br>14.12.2013<br>14.12.2013	
Approved by		
Ninon Horié	07.07.2014	
