# Supplementary material for: Experiences and Lessons from a Multicountry NIDIAG Study on Persistent Digestive Disorders in the Tropics
Source: PLoS Negl Trop Dis. 2016 Nov 3;10(11):e0004818. doi: 10.1371/journal.pntd.0004818 (PMC5094778; doi:10.1371/journal.pntd.0004818)
Supplement: S7 Laboratory SOP — (PDF) [file pntd.0004818.s013.pdf]

	**SOP Title:** How to obtain a stool sample
	**Study title**: Diagnosis of neglected tropical diseases (NTDs) in patients presenting with persistent digestive disorders (≥2 weeks) in Côte d'Ivoire, Indonesia, Mali and Nepal

1. Scope and application

This SOP describes how to obtain a stool sample for in-depth diagnostic work-up to detect parasitic (all countries) and/or bacterial and viral (all countries except Indonesia and Mali) pathogens, using a panel of various diagnostic tests.

2. Responsibilities

Function	Activities
Study physician	• Explains the purpose and procedures to study participants
Laboratory technician	• Receives the stool sample and evaluates whether it can be used for the study diagnostic work-up

3. Materials

- Stool collection container:
    - Disposable (**sterile container with sufficiently large opening, only to be used once; volume: ≥125 ml**)
    - Tightly fitting lid (e.g. screw-cap)
- Spatula (separate or integrated)
- Container label including patient-specific ID and a space to note down the patient's name and the time of stool production
- 1 plastic bag per container, in which the container can be stored after stool collection
- Clean paper for stool collection
- Gloves for handling of the sample in the laboratory

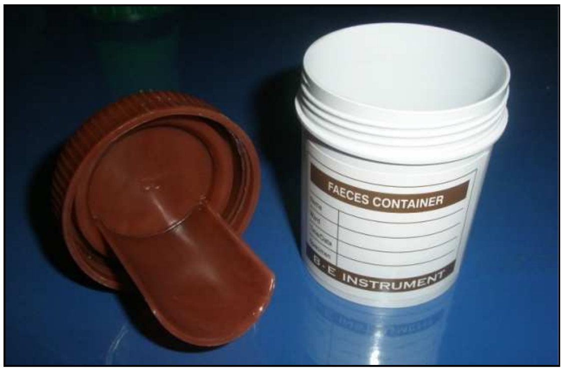

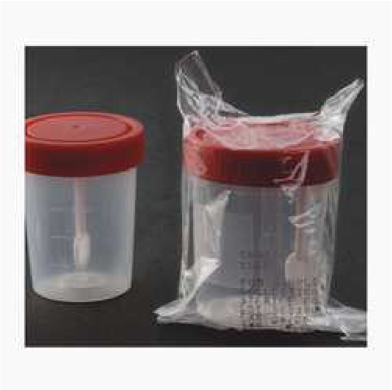

4. Stool container distribution and how to obtain the sample

- Explain to study participant why stool is needed
- Introduce the materials (stool collection container, spatula, non-transparent plastic bag)
- Instruct the individuals to use their assigned container only for their own stool
- Parents are to ensure their child's stool sample is placed into the container labeled with the child's name and the corresponding unique ID number

- Explain to the study participant the following details:
    1) How to deposit stool (on clean paper or aluminium foil in case of watery diarrhoea; in case of very young children, diapers may also be used)
    2) How to scoop the stool
    3) Specify the volume (fill the container up to a marked line at about 80% of the total height of the container; aim: ≥60 grams of stool material)
    4) How to place the stool sample into the container
    5) How to close the cap tightly and how to write down the time of stool collection on the respective space on the stool container label
    6) How to put the closed container into the plastic bag
    7) Any leftover stool should be properly discarded (e.g. thrown into the latrine or burnt)
    8) Filled containers are to be stored at room temperature and in the shade
    9) Transfer the fresh stool samples immediately to the laboratory for diagnostic processing
- Study personnel please take note:
    1) Pre-label the stool container with a printed, unique ID code (refer to respective SOP) before giving the stool collection kit to the patient.
    2) Ensure that the unique ID on the printed label corresponds to the ID that has been assigned to this patient
    3) Indicate to the patient a specific time and location for handing in of the stool container

5. Processing in the laboratory

- Process the stool material in the laboratory within 24 hours of receiving it. Times when stool material was received and processed should be noted down in the CRF as soon as possible.
- Evaluate whether the amount of stool is sufficient to perform all required diagnostic tests.
- If the amount of stool is not sufficient, try to obtain a second sample from the same patient as fast as possible, ideally on the following day. Preferably, all tests should be performed on one sufficiently large sample. If not feasible, perform the diagnostic techniques according to the priority list (refer to the SOP “Diagnostic sample flow”) on the first stool sample and once the first stool sample is used up, the remaining diagnostic techniques should be performed on the second stool sample. This must be noted in the CRF.
- If the total stool amount obtained from one individual is not sufficient to perform all techniques and no further sample can be obtained, there is a prioritization of which techniques have to be performed in a certain order. Refer to the SOP “Diagnostic sample flow” for further details.

6. Records and archives

**Appendices & Forms for completion**	
**Number**	**Title**
1	Hospital Lab Register
2	CRF

7. Document History

**Revision**	
SOP-WP2-LAB-56-V01-05 Dec 2013	Initial version by Peter Steinmann and Sören L. Becker
SOP-WP2-LAB-56-V02-09 Dec 2013	Reviewed by Elsa Murhandarwati and Katja Polman
SOP-WP2-LAB-56-V03-13 Dec 2013	Revised by Peiling Yap
SOP-WP2-LAB-56-V04-25 Mar 2014	Approved by Stefanie Knopp

**Name and function**	**Date**	**Signature**
Author		
Peter Steinmann<br>Sören L. Becker	19.11.2013<br>04.12.2013	
Reviewed by		
Elsa Murhandarwati<br>Katja Polman<br>Peiling Yap	06.12.2013<br>09.12.2013<br>13.12.2013	
Approved by		
Stefanie Knopp	25.03.2014	
