# Supplementary material for: Experiences and Lessons from a Multicountry NIDIAG Study on Persistent Digestive Disorders in the Tropics
Source: PLoS Negl Trop Dis. 2016 Nov 3;10(11):e0004818. doi: 10.1371/journal.pntd.0004818 (PMC5094778; doi:10.1371/journal.pntd.0004818)
Supplement: S8 Laboratory SOP — (PDF) [file pntd.0004818.s014.pdf]

	**SOP Title:** Formalin-ether concentration technique
	**Study title**: Diagnosis of neglected tropical diseases (NTDs) in patients presenting with persistent digestive disorders (≥2 weeks) in Côte d'Ivoire, Indonesia, Mali and Nepal

1. Scope and application

The formalin-ether concentration technique is typically conducted in reference laboratories for the concurrent diagnosis of helminth and intestinal protozoa infections in patients enrolled under the digestive syndrome of the NIDIAG study in Côte d'Ivoire, Indonesia, Mali and Nepal. By concentrating the stool sample via centrifugation, sensitivity of the microscopic detection method can be increased. With the different solutions added, fecal debris is extracted into the ether phase and parasitic elements are sedimented at the bottom.

2. Responsibilities

Function	Activities
Laboratory Technician	▪ Perform the formalin-ether concentration technique blinded to the results of the reference tests.<br>▪ Report the results in the Hospital Lab Register.
Study Nurse/Study Assistant	▪ Transcribe the results from the Hospital Lab Register to the Case Report Form (CRF).

3. Procedures

3.1 Safety

- Handle all samples as potentially infectious. Wear gloves during the procedure.
- Formalin is poisonous and an irritant, while ether is flammable and presents a risk of explosion.
- At each study site, safety precautions for handling and disposal of infectious materials should be practiced according to the laboratory safety rules of the participating hospital.

3.2 Materials and samples

3.2.1 Materials required

- Sodium acetate
- Acetic acid
- 40% formaldehyde
- 0.9% NaCl solution
- Ether
- Stool container
- Centrifuge tube [15 milliliters (ml)]
- Centrifuge stand
- Funnel
- Medical gauze
- Rubber stopper
- Spatula/ wooden stick
- Plastic pipette
- Microscopic slide

- Cover slip
- Light microscope
- Weighing scale

3.2.2 Samples

- Fresh stool (patient should hand in the stool the following morning after container has been distributed; please refer to the SOP for stool collection)

3.3 Procedures

1. Prepare the sodium acetate/acetic acid/formalin (SAF) solution by dissolving 15 grams (g) sodium acetate in a mix of 20 ml acetic acid and 40 ml 40% formaldehyde in 925 ml of water.
2. Using a weighing scale, preserve approximately 1 g of fresh stool in 10 ml of the SAF solution in a water-tight container. The stool sample should be thoroughly broken up and suspended.
3. For examination, the fixed sample is re-suspended and strained through a funnel layered with medical gauze. Rinse the container and funnel set-up with about 5 ml of SAF solution.
4. The filtrate is collected in a 15 ml tube and centrifuged at 2000 revolutions per minute (rpm) for 1 minute (min),
5. Remove the supernatant with a pipette or gently pour it out without disturbing the sediment. Add 7 ml of 0.9% NaCl to the sediment and mix with a wooden stick. Add another 3 ml of ether to the mixture.
6. Close tube with rubber stopper and shake vigorously for about 30 seconds while keeping the thumb on the stopper. Carefully remove the rubber stopper (beware of pressure build up) and centrifuge the tubes at 2000 rpm for 5 min.
7. After centrifugation, 4 layers should be detectable (see Figure 1). Pipette out the first 3 layers of ether, detritus and NaCl (saline). The remaining sediment should be less than 1 ml. If more than 1 ml of sediment is present, repeat steps 5 and 6.
8. Mix the sediment and place a drop on a microscopic slide. Add the cover slip.

7. Examine the whole slide under a light microscope for helminth eggs (10x objective) and for protozoa (40x objective with oil immersion).

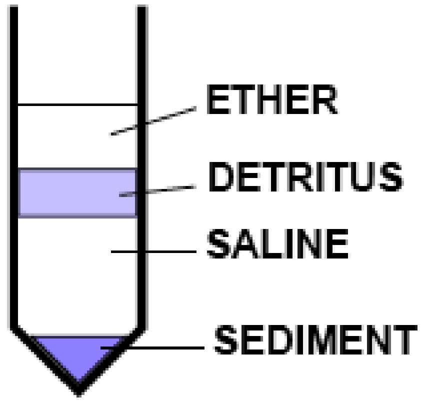

Figure 1 Layers detected after second centrifugation step

3.4 Documentation of results

- Results should be recorded for each species separately in the hospital lab register and transcribed later in the CRF.
- Record if the test was done or not, and if the test was not done, provide a reason for not doing it.
- Record if the result is POSITIVE or NEGATIVE.
- If POSITIVE, record the number of helminth eggs or protozoa and use the following semi-quantitative scheme:
  - Negative (no cysts or trophozoites in the entire sediment);

- Rare (one to five cysts or trophozoites per slide);
- Frequent (one cyst or trophozoite per observation field of 1000x magnification); and
- Very frequent (more than one cyst or trophozoite per observation field of 1000x magnification).

3.5 Waste management

Dispose remaining stool samples and slides without contaminating the local environment.

4. Records and archives

**Appendices & Forms for completion**	
**Number**	**Title**
1	Hospital Lab Register
2	CRF

5. Document History

**Revision**	
SOP-WP2-LAB-57-V01-28Oct2013	Initial version
SOP-WP2-LAB-57-V02-09Dec2013	Reviewed by Elsa Murhandarwati and Katja Polman
SOP-WP2-LAB-57-V03-13Dec2013	Revised by Peiling Yap
SOP-WP2-LAB-57-V04-21Mar2014	Reviewed by Basudha Khanal and Sören L. Becker

**Name and function**	**Date**	**Signature**
Author		
Peiling Yap	28.10.2013	
Reviewed by		
Elsa Murhandarwati<br>Katja Polman<br>Peiling Yap<br>Basudha Khanal<br>Sören L. Becker	06.12.2013<br>09.12.2013<br>13.12.2013<br>15.12.2013<br>11.02.2014	
Approved by		
Peter Steinmann	17.02.2014	
