# Supplementary material for: Experiences and Lessons from a Multicountry NIDIAG Study on Persistent Digestive Disorders in the Tropics
Source: PLoS Negl Trop Dis. 2016 Nov 3;10(11):e0004818. doi: 10.1371/journal.pntd.0004818 (PMC5094778; doi:10.1371/journal.pntd.0004818)
Supplement: S9 Laboratory SOP — (PDF) [file pntd.0004818.s015.pdf]

nidiag Better Diagnosis of Neglected Infectious Diseases	**SOP Title:** Koga agar plate culture
	**Study title**: Diagnosis of neglected tropical diseases (NTDs) in patients presenting with persistent digestive disorders (≥2 weeks) in Côte d'Ivoire, Indonesia, Mali and Nepal.

1. Scope and application

The Koga agar plate culture is a technique for the detection of helminth larvae. The principle behind this technique is that when fresh stool is incubated on the agar plate, the larvae of helminths will grow and move around the plate, leaving characteristic tracks on the surface of the agar. The larvae can subsequently be detected with microscopic examination. This SOP is applicable for the diagnostic evaluation of *Strongyloides stercoralis* and hookworms in patients enrolled under the digestive syndrome of the NIDIAG study in Côte d'Ivoire, Indonesia, Mali and Nepal.

2. Responsibilities

**Function**	**Activities**
Laboratory Technician	▪ Perform the Koga agar plate culture blinded to the results of the Baermann method.<br>▪ Report the results in the Hospital Lab Register.
Study Nurse/Study Assistant	▪ Transcribe the results from the Hospital Lab Register to the Case Report Form (CRF).

3. Procedures

3.1 Safety

- Handle all samples as potentially infectious. Wear gloves during the procedure. This is extremely important because the Koga agar plate culture allows *S. stercoralis* and hookworm larvae to develop into filariform L_3 larvae that are able to infect human hosts percutaneously through the intact skin.
- At each study site, safety precautions for handling and disposal of infectious materials should be practiced according to the laboratory safety rules of the participating hospital. Gloves must be weared during all procedures.

3.2 Materials and samples

3.2.1 Materials required

- Agar media (consisting of 1.5% agar, 0.5% meat extract, 1.0% peptone and 0.5% NaCl)
- Agar plate, 100 millimeters (mm) in diameter
- Incubator
- Plastic pipette
- Spatula
- SAF solution (see SOP for Formalin-ether concentration technique for preparation of SAF)
- Centrifuge tube [15 milliliters (ml)]
- Centrifuge
- Microscopic slide
- Cover slip
- Light microscope

3.2.2 Samples

- Fresh stool (patient should hand in the stool the following morning after container has been distributed; please refer to the SOP for stool collection)

3.3 Procedures

1. To prepare 1000 ml of agar media, weigh out 15 grams (g) of agar, 5 g of meat extract, 10 g of peptone and 5 g of NaCl and dissolve in water (top up to 1000 ml) over a warm water bath.
2. Distribute 10 ml of freshly prepared agar media to each clean agar plate and allow the agar to solidify and cool to room temperature. Agar plates can be prepared and stored at 2-8°C in sealed containers to avoid loss of moisture (do not freeze) for not more than a week.
3. Scoop approximately 2 g of a fresh stool sample and place it at the center of the agar plate. Cover the agar plate (please see Figure 1).

3\. Incubate the stool-filled agar plate in an incubator (26 – 33ºC) for 48 hours (hr).

4\. Examine the agar plate visually for characteristic tracks of the larvae.

5\. Place the agar plate under a light microscope and examine the whole plate for the presence of larvae without removing the cover (x40 magnification).

6\. Finally, pipette 15 ml of SAF to each plate to wash the agar surface. The washings are collected with the same pipette, transferred to a 15 ml centrifuge tube and subjected to centrifugation (2000 revolution/minute (min) for 5 min).

7\. Carefully pour out the supernatant without disturbing the sediment. Pipette the sediment onto a microscopic slide and examine the slide under a light microscope (x100 – 400 magnification). Differentiation of the larvae of both hookworm and *S. stercoralis* must be made.

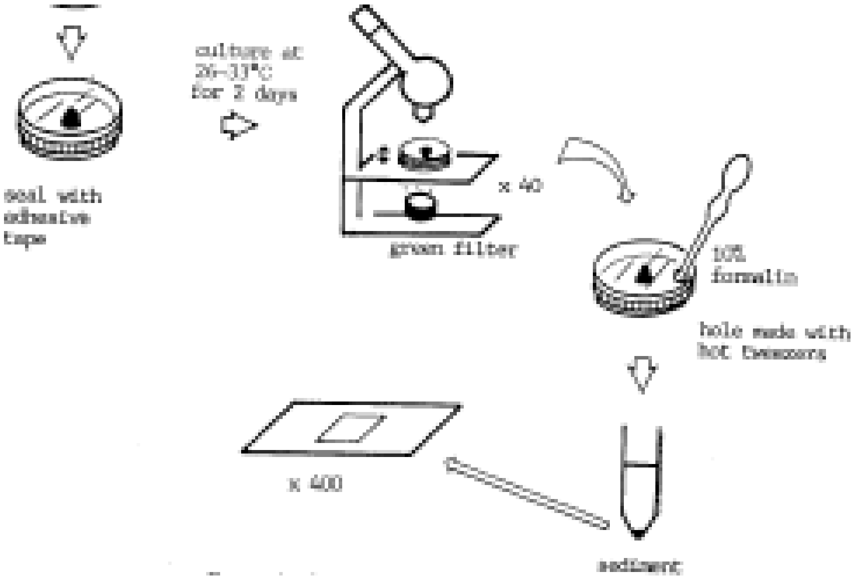

Figure 1 Steps for the Koga agar plate method

3.4 Documentation of results

- Record the results in the hospital lab register and transcribe them later to the CRF.
- Record if the test was done or not, and if the test was not done, provide a reason for not doing it.
- Record if the result is POSITIVE or NEGATIVE.
- If POSITIVE, record the species of larvae detected and the number of larvae of each species observed on the microscopic slide (see Figure 2 for differences between hookworm and *Strongyloides* L_3 larvae).

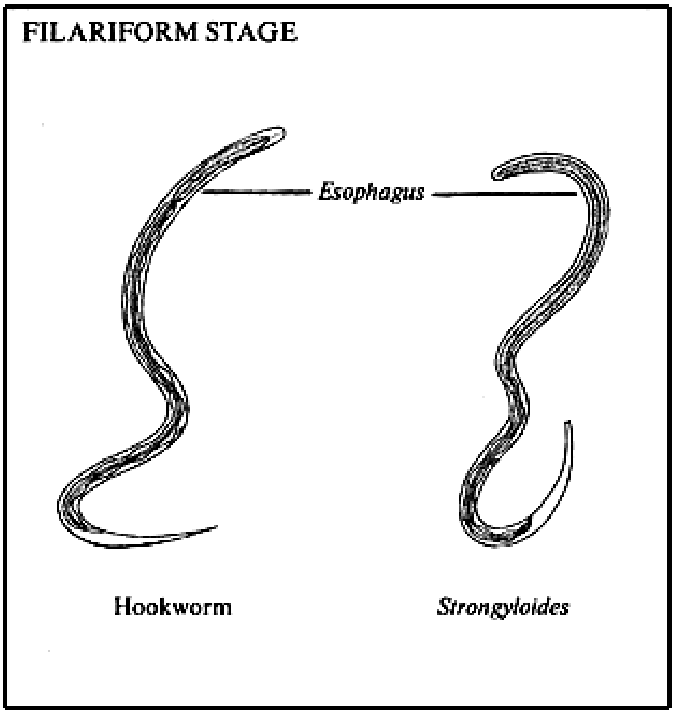

Figure 2 Differences between hookworm and *Strongyloides* L_3 larvae (*Strongyloides* has a longer esophagus than hookworm, and *Strongyloides* has a slit in the tail while hookworm has a pointed tail)

3.5 Preservation of culture sediments

- Preservation of Koga sediments should be done for microscopically positive sediments. The aim is to use these 'purified' helminth larvae (*S. stercoralis* or hookworm) for the development and validation of stool-based rapid diagnostic tests (RDTs) for the diagnosis of *S. stercoralis*.
- Preservation procedure: Mix 1 ml of the positive sediment with 1 ml of ethanol (if available 96%) in a Greiner 146361 tube. Transfer the aliquots to a freezer as soon as possible. If possible, store the aliquots -80°C. If this is not possible in your study site, store the sample at -20°C. If no freezer is available at all at the study sites, store the samples in the refrigerator.
- Aliquot labelling of the Koga-positive samples: Every tube containing a sediment of a sample that was positive for helminth larvae in the Koga technique is labelled with the number of the stool sample used (check SOP on Numbering Systems, SOP-WP6-DOC-02-V5-13Jun2014), followed by the two letters KO for Koga.
    - **Example**: The label for a Koga-positive sediment collected from the stool sample number 2 of patient 31 in the Tulehu hospital site in Indonesia is: 31031-Dx-ST2-KO.

3.6 Waste management

- Dispose remaining stool samples, agar plates and microscope slides as biohazards without contaminating the local environment.

4. References

- Koga K, Kasuya S, Khamboonruang C et al., 1991. A modified agar plate method for detection of *Strongyloides* stercoralis. American Journal of Tropical Medicine and Hygiene (45): 518 – 521

5. Records and archives

**Appendices & Forms for completion**	
**Number**	**Title**
1	Hospital Lab Register

2	CRF

6. Document History

**Revision**	
SOP-WP2-LAB-58-V01-28Oct2013	Initial version
SOP-WP2-LAB-58-V02-09Dec2013	Reviewed by Elsa Herdiana, Basudha Khanal and Katja Polman
SOP-WP2-LAB-58-V03-13Dec2013	Revised by Peiling Yap
SOP-WP2-LAB-58-V04-27Jan2014	Revised by Peiling Yap and Sören Becker
SOP-WP2-LAB-58-V05-23Jun2014	Adaptation of the chapter '3.5 Preservation of culture sediments'
SOP-WP2-LAB-58-V06-16Jan2015	Adaptation of chapter '3.3 Procedures'

**Name and function**	**Date**	**Signature**
Author		
Peiling Yap	28.10.2013	
Reviewed by		
Elsa Herdiana<br>Katja Polman<br>Basudha Khanal	06.12.2013<br>09.12.2013<br>10.12.2013	
Approved by		
Sören L. Becker	16.01.2015	
