# Supplementary material for: Experiences and Lessons from a Multicountry NIDIAG Study on Persistent Digestive Disorders in the Tropics
Source: PLoS Negl Trop Dis. 2016 Nov 3;10(11):e0004818. doi: 10.1371/journal.pntd.0004818 (PMC5094778; doi:10.1371/journal.pntd.0004818)
Supplement: S10 Laboratory SOP — (PDF) [file pntd.0004818.s016.pdf]

nidiag Better Diagnosis of Neglected Infectious Diseases	**SOP Title:** Direct fecal smear technique
	**Study title**: Diagnosis of neglected tropical diseases (NTDs) in patients presenting with persistent digestive disorders (≥2 weeks) in Côte d'Ivoire, Indonesia, Mali and Nepal

1. Scope and application

Direct fecal smear technique is the simplest and easiest technique to facilitate detection of intestinal parasites that infected subjects pass in their feces. The presence of intestinal protozoa (trophozoites or cysts) or helminth eggs can be observed directly with a light microscope. A small amount of fresh feces is mixed with either saline (to detect the protozoa motility) or lugol/iodine solution (to reveal the parasite structure). This SOP is applicable for the diagnostic evaluation of intestinal parasites in patients enrolled under the digestive syndrome of the NIDIAG study in Côte d'Ivoire, Indonesia, Mali and Nepal.

2. Responsibilities

Function	Activities
Laboratory Technician	▪ Performing the direct fecal smear technique blinded to the results of the reference tests.<br>▪ Reporting the results in the Hospital Lab Register.
CRF person	▪ Transcribing the results from the Hospital Lab Register to the Lab Report Form of the Case Report Form (CRF).

3. Procedures

3.1 Safety

- Handle all samples as potentially infectious. Wear gloves during the procedure.
- Practice safety precautions for handling and disposal of infectious materials.

3.2 Materials and samples

3.2.1 Materials required

- Wooden applicator sticks/match
- Object glass
- Cover-slip
- Pen/marker for indelible labeling
- Isotonic saline solution (0.85%; 8.5g/l)
- Lugol's iodine (1% solution)
- Pipettes
- Microscopic slide
- Light microscope

3.2.2 Samples

- Fresh stool (please refer to the SOP for stool collection)
- Comment: Stool samples should be analyzed on the day of collection

3.3 Procedures

1. Place a drop of saline on the centre of the left half of the slide and a drop of Lugol's solution on the centre of the right half of the slide on a microscopic slide, which has been labeled with a NIDIAG patient number (see Fig. 1).

2. With a wooden applicator stick or match, pick up a small portion of the stool specimen (size of match head) and mix with a drop of saline to form suspension (see Fig. 2).
3. Similarly, pick up a small portion of the stool specimen and mix with Lugol's solution to form suspension.

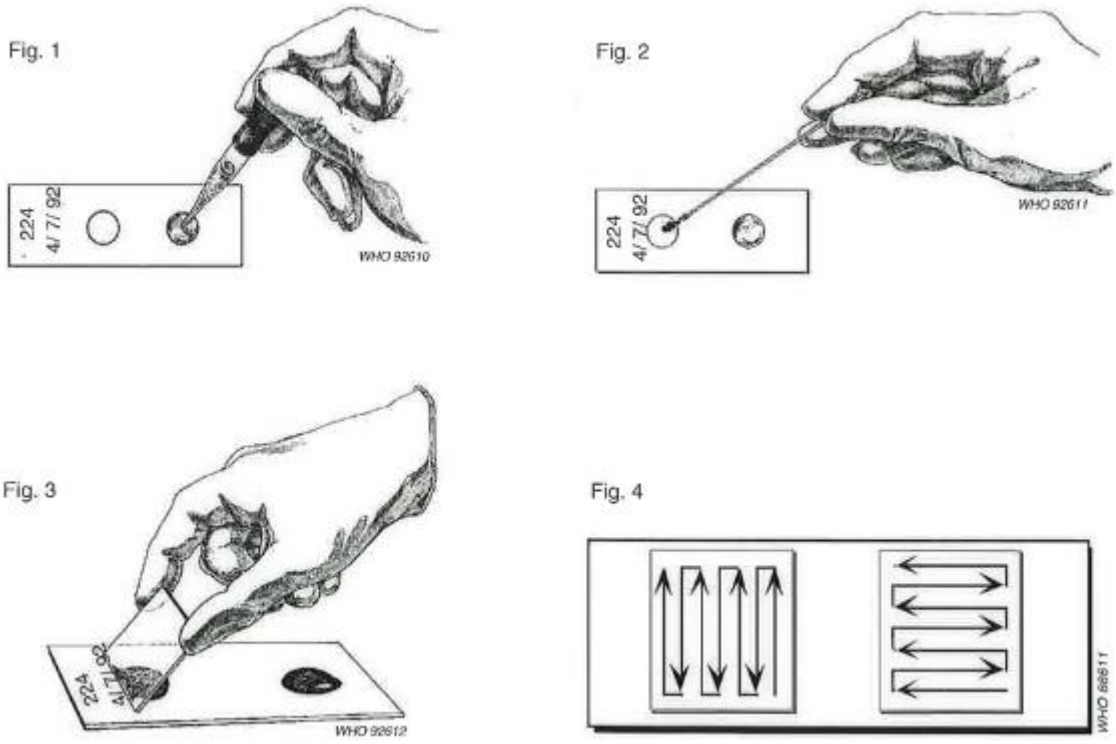

Figure 1-4. Preparation for Direct Smear Technique (adopted from WHO, 1994)

4. Cover each drop with a cover slip by holding the cover slip at an angle, touch the edge of the drop and gently lower the cover slip onto the slide to reduce the possibility of air bubbles in the smear (see Fig. 3).
5. Put the slide under a light microscope with 10x objective. Examine the entire cover slip area by moving the slide systematically backwards and forwards, or up and down (see Fig.4).
6. Switch to 40x objective lenses when suspected parasites are seen.

3.4 Documentation of results

- Results should be recorded directly in the Hospital Lab Register.
- Record if the result is POSITIVE or NEGATIVE.
- If POSITIVE, record all detected species and its trophozoites/cysts number (for intestinal protozoa) or eggs (for helminth) for each solution.
- Example:
    - in Saline solution:

1. *Ascaris lumbricoides*, no.of eggs: 5
2. *Giardia intestinalis/lamblia*, no.of cysts: 6

- In Lugol's solution:

1. *Ascaris lumbricoides*, no.of eggs: 3
2. *Giardia intestinalis/lamblia*, no.of cysts: 6

3.5 Waste management

- Dispose remaining stool samples and slides with fecal thick smears without contaminating the local environment.

4. References

WHO, 1994. Bench Aids for the diagnosis for intestinal parasites

5. Records and archives

**Appendices & Forms for completion**	
**Number**	**Title**
1	Hospital Lab Register
2	CRF

6. Document History

**Revision**	
SOP-WP2-LAB-59-V01-13JAN2014	Initial version
SOP-WP2-LAB-59-V02-11FEB2014	Review by Sören L. Becker

**Name and function**	**Date**	**Signature**
Author		
Elsa Murhandarwati	13.01.2014	
Reviewed by		
Sören L. Becker	11.02.2014	
Approved by		
Katja Polman	26.02.2014	
