# Supplementary material for: Experiences and Lessons from a Multicountry NIDIAG Study on Persistent Digestive Disorders in the Tropics
Source: PLoS Negl Trop Dis. 2016 Nov 3;10(11):e0004818. doi: 10.1371/journal.pntd.0004818 (PMC5094778; doi:10.1371/journal.pntd.0004818)
Supplement: S11 Laboratory SOP — (PDF) [file pntd.0004818.s017.pdf]

	**SOP Title:** Preparation of aliquots for molecular post-hoc testing
	**Study title**: Diagnosis of neglected tropical diseases (NTDs) in patients presenting with persistent digestive disorders (≥2 weeks) in Côte d'Ivoire, Indonesia, Mali and Nepal.

1. Scope and application

This SOP explains how to prepare aliquots for downstream molecular analyses of stool samples collected during the NIDIAG study on persistent digestive disorders in Côte d'Ivoire, Indonesia, Mali and Nepal. The aliquots prepared using this SOP will be analyzed with molecular tools in reference laboratories.

2. Responsibilities

**Function**	**Activities**
Laboratory Technician	▪ Prepare three aliquots of each stool sample<br>▪ Label the tubes and organize the storage<br>▪ Prepare the transfer to the reference laboratory
Country PI	▪ Prepare a material transfer agreement (MTA) with the concerned institutions and airlines in your country<br>▪ Set up a system that allows for regular specimen transfer to reference laboratories within your country and to specialized laboratories in Europe
Local/Country Data Manager	▪ Record all prepared aliquots in a "Study Specimens Log" (refer to the SOP "Numbering System to be used in NIDIAG WP2 studies" for details)

3. Material

4.4 3.1 Safety

- Handle all samples as potentially infectious.
- Wear gloves during the procedure.
- Practice safety precautions for handling and disposal of infectious materials.

4.5 3.2 Materials and samples

3.2.1 Materials required

- 1mL or 2 mL sterile Eppendorf tube
- Clean spatula
- 1000 µL Pipette
- Large orifice 1mL Tips
- Freezer (-80°C or -20°C)
- Scale
- 96% EtOH
- Labels with unique ID for each specimen (see also SOP on "Stool sample collection", SOP on "Diagnostic sample flow", SOP on "Numbering systems")

3.2.2 Sample

- Fresh stool (please refer to the SOP for stool collection)
- Aliquots should be prepared and stored as soon as possible.

4. Procedures

4.1 Important pre-analytical considerations

- Prepare the aliquots for molecular post-hoc testing immediately upon arrival of the fresh stool sample in the laboratory.
- Perform all other diagnostic procedures **after** the aliquots for molecular tests have been prepared.
- Prepare 3 aliquots of each stool sample to allow for the following examinations:

	**Intended use**	**To be examined in**
Aliquot no. 1	Multiplex PCR for intestinal protozoa and helminths	Leiden, The Netherlands
Aliquot no. 2	Microbiome analysis and post-hoc diagnostic testing for 'new', emerging pathogens (bacteria, viruses, parasites)	Basel, Switzerland
Aliquot no. 3	Multiplex PCR for bacterial and viral pathogens<br>Molecular characterization of *Strongyloides stercoralis* genotypes	Homburg, Germany

4.2 Preparation of the aliquot

- Use an empty Eppendorf tube in order to perform the tare on the scale.
- Transfer a given amount of stool into the Eppendorf tube in the following way:
  - If the sample is solid, transfer 500 mg to 1g into a clean Eppendorf using a clean spatula.
  - If the sample is liquid, transfer 500 µL to 1mL into a fresh Eppendorf using the pipette with large orifice tips.
- Prepare **three aliquots of each stool sample!**
- Label the sample correctly and record it in the "Study Specimens Log" (refer to the SOP "Numbering System to be used in NIDIAG WP2 studies" for details).
- Add 1-2 mL of 96% EtOH to the Eppendorf tube containing the stool sample and gently vortex for 30 seconds.
- Transfer the aliquots to a freezer as soon as possible. If possible, store the aliquots -80°C. If this is not possible in your study site, store the sample at -20°C. If no freezer is available at all at the study sites, store the samples in the refrigerator.
- When a sufficient amount of aliquots has been stored, send the samples to the designated reference laboratories. Importantly, all aliquots have to be sent first to the Swiss Tropical and Public Health Institute (Swiss TPH) in Basel, Switzerland and will then be distributed to the different European reference laboratories.

4.3 Documentation of the results

- Record in the CRF if the aliquoting was done or not.
- Document every aliquot in the "Study Specimens Log" (refer to the SOP "Numbering System to be used in NIDIAG WP2 studies" for details).

4.4 Waste management

- Dispose used spatula and tips without contaminating the local environment.

5. References

- Leiden University Medical Center Sample Preservation Sheet. Leiden, The Netherlands. http://www.lumc.nl/con/1040/81028091348221/811071047002556/ (accessed: 22 January, 2014)
- van Lieshout L, Verweij JJ: **Newer diagnostic approaches to intestinal protozoa.** *Curr Opin Infect Dis* 2010, **23:**488-493.

6. Records and archives

**Appendices & Forms for completion**	
**Number**	**Title**
1	Hospital Lab Register
2	CRF
3	SOP Numbering System to be used in NIDIAG WP2 studies
4	Leiden University Medical Center Sample Preservation Sheet
5	SOP on Stool sample collection
6	SOP on Diagnostic sample flow

7. Document History

**Revision**	
SOP-WP2-LAB-10-V01-07Feb2014	Initial version
SOP-WP2-LAB-10-V02-22Feb2014	Revision by Sören L. Becker
SOP-WP2-LAB-10-V03-23Feb2014	Approval by Lutz von Müller

**Name and function**	**Date**	**Signature**
Author		
Pierre Schneeberger	07.01.2014	
Reviewed by		
Sören L. Becker	22.01.2014	
Approved by		
Lutz von Müller	23.02.2014	
