# Supplementary material for: Experiences and Lessons from a Multicountry NIDIAG Study on Persistent Digestive Disorders in the Tropics
Source: PLoS Negl Trop Dis. 2016 Nov 3;10(11):e0004818. doi: 10.1371/journal.pntd.0004818 (PMC5094778; doi:10.1371/journal.pntd.0004818)
Supplement: S12 Laboratory SOP — (PDF) [file pntd.0004818.s018.pdf]

	**SOP Title:** Urine POC-CCA RDT for the diagnosis of *Schistosoma mansoni*
	**Study title**: Diagnosis of neglected tropical diseases (NTDs) in patients presenting with persistent digestive disorders (≥2 weeks) in Côte d'Ivoire, Indonesia, Mali and Nepal.

1. Scope and application

The point-of care circulating cathodic antigen (POC-CCA) urine cassette test is a rapid diagnostic test (RDT) for the qualitative presumptive detection of an active *Schistosoma* infection, specifically due to *S. mansoni.* This SOP describes how to perform the POC-CCA urine cassette test in order to evaluate the presence of *S. mansoni* in patients and asymptomatic controls in the two African countries of the NIDIAG digestive study (Côte d'Ivoire and Mali).

2. Responsibilities

Function	Activities
Laboratory Technician	▪ Perform the POC-CCA urine cassette test for the diagnosis of *S. mansoni* infection.<br>▪ Report the results in the Hospital Lab Register.<br>▪ Indicate the results in the Case Report Form (CRF).

3. Material

3.1. Safety

- Handle all samples as potentially infectious. Wear gloves and lab coats during the procedure.
- Practice safety precautions for handling and disposal of infectious materials.

3.2. Materials and samples

3.2.1. Materials required

- POC-CCA urine cassette test
- 3 mL bottle of buffer
- Urine collection device (plastic pipette)

3.2.2. Samples

- Urine (please refer to the SOP on urine collection)
- Urine samples should be examined on the day of collection.

4. Procedures

4.1. Preparation of the test

- Ensure all reagents are brought to room temperature (20-25°C) before beginning the assay
- Ensure the pouches containing the tests are not torn
- Remove the test cassette and collection device from their pouches just prior to use.

4.2. Assay procedure

- Homogenize the urine sample (shake it well)
- Squeeze the pipette top and insert the tip into the urine sample
- Allow the sample to fill up by gently releasing the top
- Transfer 1 drop of urine to the circular well of the test cassette by gently squeezing the top

- Allow the sample to absorb entirely into the specimen pad within the circular well
- Hold the buffer bottle vertically, approximately 1 cm above the circular well
- Add 1 drop of buffer solution to the circular well
- Read the result exactly **20 min** after adding buffer to the test cassette
- Any results read after ≥ **25 min** should be considered to be **invalid** and must be repeated
- The blue control line must turn pink. If the control line stays blue, the test should be considered as invalid
- Any line in the test area should be considered as positive (see grading procedure below)
- **Visualized procedure summary below:**

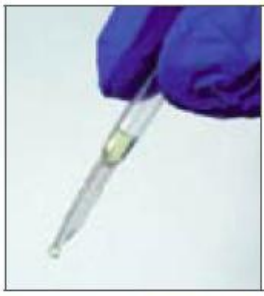

- Squeeze the pipette bulb and insert the tip into the urine sample.
- Allow the sample to fill up by gently releasing the bulb.

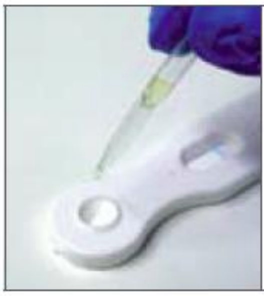

- Transfer 1 drop of urine to the circular well of the test cassette by gently squeezing the bulb.
- Allow the sample to absorb entirely into the specimen pad within the circular well.

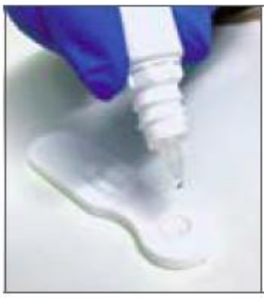

- Hold the Buffer bottle vertically and 1cm above the circular well.
- Add 1 drop of Buffer.

POSITIVE

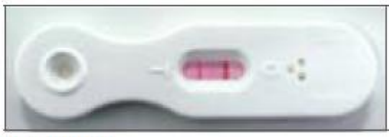

Control band turns pink.
A band is present in the test T area.
The test is positive for Bilharzia.

NEGATIVE

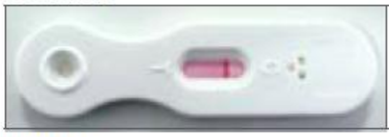

Control band turns pink.
No test T band present.
Demonstrates the test was performed correctly but no Bilharzia antigens were detected.

INVALID

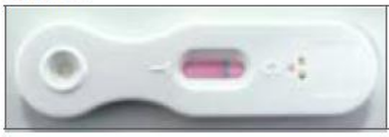

Control line stays blue.
Only a pink control line should be considered positive.
The test is invalid and should be repeated.

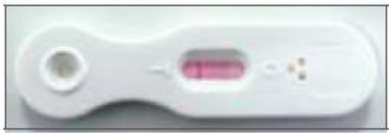

A test line with no control line.
A pink control line must be present.

4.3. Documentation of results

- Results should be recorded either in the Hospital Lab Register (and later transcribed into the CRF) or directly in the CRF. If the results are not recorded directly in the CRF, the study site manager/study nurse needs to double-check and verify the transcription of the results from the Hospital Lab Register to the CRF regularly.
- Record whether the test was done or not, and provide a reason if it was not done.
- Record whether the result is POSITIVE or NEGATIVE.
- In case of an invalid test result, repeat the RDT.
- Record the line intensity of the test line in the CRF (e.g. trace, 1+ (clearly positive, but faint line), 2+ (clearly positive with strong line), 3+ (strongly positive with very strong line).

4.4. Waste management

- Dispose remaining potentially contaminated material and urine without contaminating the local environment.

5. References

- Colley DG, Binder S, Campbell C, King CH, Tchuem Tchuenté LA, N'Goran EK, Erko B, Karanja DM, Kabatereine NB, van Lieshout L, Rathbun S, 2013. A five-country evaluation of a point-of-care circulating cathodic antigen urine assay for the prevalence of *Schistosoma mansoni*. *Am J Trop Med Hyg* 88, 426-432.

- Coulibaly JT, N'Gbesso YK, Knopp S, N'Guessan NA, Silué KD, van Dam GJ, N'Goran EK, Utzinger J, 2013. Accuracy of urine circulating cathodic antigen test for the diagnosis of *Schistosoma mansoni* in preschool-aged children before and after treatment. *PLoS Negl Trop Dis* 7, e2109.

- Coulibaly JT, Knopp S, N'Guessan NA, Silué KD, Fürst T, Lohourignon LK, Brou JK, N'Gbesso YK, Vounatsou P, N'Goran EK, Utzinger J, 2011. Accuracy of urine circulating cathodic antigen (CCA) test for *Schistosoma mansoni* diagnosis in different settings of Côte d'Ivoire. *PLoS Negl Trop Dis* 5, e1384.

- Ayele B, Erko B, Legesse M, Hailu A, Medhin G, 2008. Evaluation of circulating cathodic antigen (CCA) strip for diagnosis of urinary schistosomiasis in Hassoba school children, Afar, Ethiopia. *Parasite* 15, 69-75.

6. Records and Archives

**Appendices & Forms for completion**	
**Number**	**Title**
1	Hospital Lab Register
2	CRF

7. Document History

**Revision**	
SOP-WP2-LAB-61-V01-20Nov2013	Initial version
SOP-WP2-LAB-61-V02-28Nov2013	Revision by Jean T. Coulibaly and Sören L. Becker
SOP-WP2-LAB-61-V03-05Dec2013	Revision by Jürg Utzinger and Katja Polman
SOP-WP2-LAB-61-V04-25Mar2014	Approval by Stefanie Knopp

**Name and function**	**Date**	**Signature**

Author		
Pierre Schneeberger	20.11.2013	
Reviewed by		
Jean T. Coulibaly<br>Sören L. Becker<br>Jürg Utzinger<br>Katja Polman	22.11.2013<br>03.12.2013<br>05.12.2013<br>09.12.2013	
Approved by		
Stefanie Knopp	25.03.2014	
