# Supplementary material for: Experiences and Lessons from a Multicountry NIDIAG Study on Persistent Digestive Disorders in the Tropics
Source: PLoS Negl Trop Dis. 2016 Nov 3;10(11):e0004818. doi: 10.1371/journal.pntd.0004818 (PMC5094778; doi:10.1371/journal.pntd.0004818)
Supplement: S13 Laboratory SOP — (PDF) [file pntd.0004818.s019.pdf]

	**SOP Title:** Diagnostic sample flow (including techniques, handling and storage, preservation and shipment)
	**Study title**: Diagnosis of neglected tropical diseases (NTDs) in patients presenting with persistent digestive disorders (≥2 weeks) in Côte d'Ivoire, Indonesia, Mali and Nepal.

1. Scope and application

This SOP provides an overview on diagnostic procedures in the NIDIAG study on persistent digestive disorders. Specific details on the respective laboratories are given in the corresponding SOPs while the sample flow and work-up are described in this document.

2. Responsibilities

Function	Activities
Study physician	• Explain in detail the sample collection procedures to the patient (i.e. how to fill the sample container, amount of stool needed, when to hand in the sample, etc), provide brochure if available<br>• Provide a properly labelled container to patient.
Laboratory technician	• Receive sample from patient, check labelling (refer to SOP on numbering & labeling system), record whether amount is sufficient.<br>• Perform the diagnostic tests (see respective SOPs).<br>• Record the results in the Lab Register.<br>• Prepare samples for storage/transportation to reference laboratory (see SOP 'Preparation of aliquots for molecular post-hoc testing').
Study nurse/ Study assistant	• Transcribe the results from the Lab Register to the Case Report Form (CRF).

3. Diagnostic techniques and procedures

3.1 Urine sample

The only test to be performed on fresh urine samples is the POC-CCA RDT for diagnosis of *Schistosoma mansoni*. Due to the epidemiology of this trematode, the test will only be done in the African study sites (Côte d'Ivoire and Mali). See SOP on the 'rapid diagnostic test POC-CCA for the diagnosis of *S. mansoni'*.

3.2 Stool samples

To have sufficient stool material for all diagnostic procedures, a minimum of ~60 grams of stool is needed. Preferably a container of minimum 125 ml is provided to the patient, which should then be filled at least two-thirds full to a marked line.

- When the amount of stool is not sufficient, try to obtain a second stool sample (country-specific agreement). This must be noted in the CRF.
- If a sufficient amount of stool cannot be obtained, there is a need for prioritisation of the employed diagnostic tests in the following order:
    1. Aliquot for molecular post-hoc testing (~3 g of stool) (see SOP on 'Preparation for post-hoc molecular testing')
    2. Aliquot for bacteriology (only in case of diarrhoea). Of note: No bacteriology will be performed in Indonesia.
    3. Direct faecal smear
    4. Kato-Katz technique
    5. Formalin-ether concentration technique
    6. RDT for *Giardia* & *Cryptosporidium*

7. Baermann technique
8. Mini-FLOTAC
9. Koga agar plate
10. Kinyoun staining or further modified acid-fast staining procedure for *Cryptosporidium* spp., *Cyclospora cayetanensis*, *Cystoisospora belli*
11. Preservation of 10% of all samples in SAF medium (for later microscopy, e.g. quality control)

- Note: Even if the amount of stool is sufficient for all diagnostic tests, it will be logistically easier to adhere to the aforementioned list of subsequent examinations throughout the whole study process and to process every stool sample in this order.

Attempts should be made to obtain the stool sample before (empiric) treatment is started. However, the NIDIAG project is a non-interventional study and the common clinical practice (e.g. during outpatient consultations) will not be influenced.

These are the volumes needed:

Specimen	Tests for which used	Total volume collected
Urine	Urine CCA cassette test for *Schistosoma* spp	10 ml
Stool	Bacterial cultures (*Campylobacter, Shigella, Aeromonas, Salmonella* spp)	10 g
	Stool microscopy (direct examination, double Kato-Katz thick smear, mini-FLOTAC, Baermann, concentration techniques)	50 g
	Molecular post-hoc testing (on preserved stool samples)	3 g

4. Sample storage and preservation

Refer to the SOP on “Preparation of aliquots for molecular post-hoc testing” for details. In brief, samples should be stored in 96% ethanol at the coldest possible temperature on each site (-80°C, -20°C or 4°C).

5. Shipment of samples

Refer to the SOP on “Preparation of aliquots for molecular post-hoc testing” for details.

Importantly, all aliquots have to be sent first to the Swiss Tropical and Public Health Institute (Swiss TPH) in Basel, Switzerland and will then be distributed to the different European reference laboratories.

<u>All samples should be sent to the Swiss TPH within 6 months of sample collection.</u>

6. Records and archives

Appendices & Forms for completion	
**Number**	**Title**
1	CRF

7. Document History

**Revision**	
SOP-WP2-LAB-62-V1-05Dec2013	Initial version by Sören Becker
SOP-WP2-LAB-62-V2-02Apr2014	Revision based on multiple feedback rounds
SOP-WP2-LAB-62-V3-29May2014	Further revision by Sören Becker and approval by Peiling Yap

**Name and function**	**Date**	**Signature**
Author		
Sören L. Becker	05.12.2013	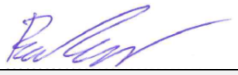
Reviewed by		
Elsa Murhandarwati<br>Suman Rijal<br>Asian workshop members<br>Sören L. Becker	06.12.2013<br>16.12.2013<br>02.04.2014<br>26.05.2014	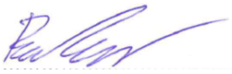
Approved by		
Peiling Yap	29.05.2014	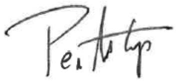
