# Supplementary material for: Experiences and Lessons from a Multicountry NIDIAG Study on Persistent Digestive Disorders in the Tropics
Source: PLoS Negl Trop Dis. 2016 Nov 3;10(11):e0004818. doi: 10.1371/journal.pntd.0004818 (PMC5094778; doi:10.1371/journal.pntd.0004818)
Supplement: S14 Laboratory SOP — (PDF) [file pntd.0004818.s020.pdf]

nidiag – Better Diagnosis of Neglected Infectious Diseases	**SOP title : Obtaining a midstream sample of urine (Digestive syndrome)**
	Study: Diagnosis of neglected tropical diseases (NTDs) in patients presenting with persistent digestive disorders (≥2 weeks) in Côte d'Ivoire, Indonesia, Mali and Nepal.

1. Scope and application

This document contains instructions on how to obtain a midstream sample of urine

2. Responsibilities

Function	Activities
Laboratory technician	• *Obtain urine samples*<br>• *Follow the procedures*

3. Procedures

3.1 Precautions

- All urine samples are potentially infectious. Respect the universal precautions. USE SINGLE-USE GLOVES DURING THE ENTIRE PROCEDURE!

3.2 Required material

- Single-use, plastic receptacle for urine
- Single-use, non-sterile gloves
- Marker

3.3 Procedure

- Put on single-use gloves
- Write the patient number, the date and the hour on the receptacle
- Give the receptacle to the patient
- Explain to the patient how to obtain a midstream urine specimen
- Ask the patient to produce a urine sample in a local rest room:
    - Minimum 50 ml
    - Midstream
- Close the cap of the sampling container tightly to avoid any contamination.
- Use the urine within 4 hours after obtaining the sample.

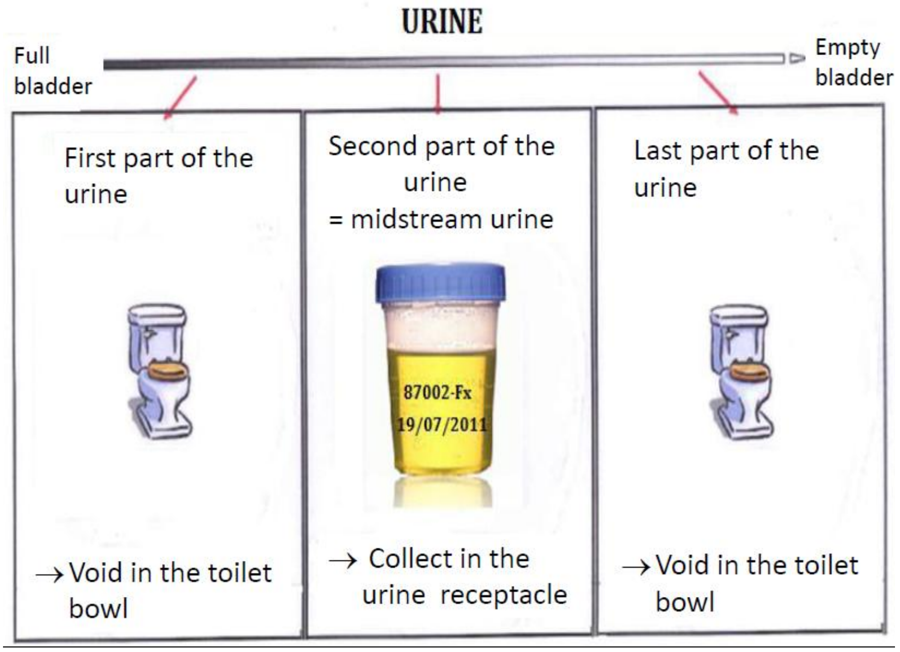

4. Records and archives

Appendices & forms to complete	
**Number**	**Title**
NA	Not applicable

5. Document history

Revision	
SOP-WP2-LAB-36-V1.1-13Aug2012	Initial version
SOP-WP2-LAB-64-V1-16Jul2014_DIG	Initial version modified by Jean T. Coulibaly for digestive syndrome

Name and function	Date	Signature
Author		
Tine Verdonck	19/07/2012	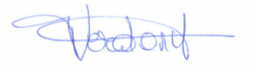
Reviewed by		
Jean T. Coulibaly	16/07/2014	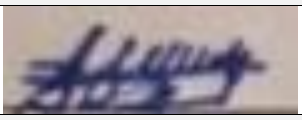
Approved by		
Ninon Horié	04/08/2014	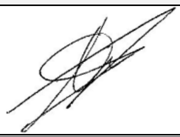
