# Supplementary material for: Experiences and Lessons from a Multicountry NIDIAG Study on Persistent Digestive Disorders in the Tropics
Source: PLoS Negl Trop Dis. 2016 Nov 3;10(11):e0004818. doi: 10.1371/journal.pntd.0004818 (PMC5094778; doi:10.1371/journal.pntd.0004818)
Supplement: S1 Quality SOP — (PDF) [file pntd.0004818.s021.pdf]

	**SOP Title:** Procedure for obtaining informed consent
	Project/study: This SOP applies to all NIDIAG studies

1 1. Scope and application

Before a patient decides to take part in a NIDIAG study, he/she should be informed of the study aim and objectives, the required clinical examinations, and the benefits and risks related to his/her participation. If the patient confirms his/her willingness to participate in the study, he/she should sign and date the Informed Consent Form (ICF).
The objective of this standard operating procedure (SOP) is to outline the procedures and responsibilities for obtaining the freely given informed consent from participants in NIDIAG studies.

2 2. Responsibilities

Function	Activities
Site investigator	- Ensuring that the patient receives the full information that allows him/her to take an informed decision, and that he/she has fully understood it.<br>- Obtaining and documenting the freely given informed consent of the patient (and/or parent/guardian/legal representative) before he/she undergoes any study-related procedures (including screening procedures) which are not part of routine clinical care.<br>- Storing ICF in appropriate place<br>- Documenting consent withdrawals during the study
Site Quality Manager and External Monitor	- Verifying that the ICF is completed, dated and signed by the investigator and the patient (and/or parent/guardian/ legal representative)<br>- Verifying consent withdrawals have been properly documented

3 Procedures

3.1 Timing

- Obtain informed consent from all patients (or parent/guardian/legal representative) prior to any study specific tests or evaluations, including screening procedures, which fall outside the routine medical care available locally for the condition under study.

3.2 Information and discussion

3.2.1 Information

- Give the patient (and/or parent/guardian/legal representative) adequate, full and comprehensive information about the study, according to the information contained in the Informed Consent Form (ICF) and according to the principles of the Helsinki Declaration, WHO and ICH Guidelines and any applicable national regulations.
- In particular, explain the following:
  - The study purposes;
  - The study procedures, and in particular, all additional diagnostic procedures to be conducted according to the study protocol;
  - The duration of the participation for the participant, the number of study visits and the duration of the follow-up period according to the study protocol;
  - The risks or inconveniences to the participant, including the risks related to diagnostic procedures that are not part of the routine diagnostic workup (ex. extra blood sampling, etc...);
  - The expected benefits for the patient, if any, and the expected benefits for the community;
  - The alternative procedure/s that are available, and their benefits and risks;
  - The reimbursement for travel expenses, as described in the protocol;

- That the subject (and/or their parent/guardian/legal representative) may refuse to participate or withdraw from the study, at any time (also during the trial), without penalty or loss of benefits;
- Mechanisms of indenisation in case of harm derived from the participation in the study;
- The fact that any trial records identifying the subject will be kept confidential and that the subject's identity will remain confidential also when the results of the trial are being published;
- The fact that the subject (and/or their parent/guardian/legal representative) will be informed if new information becomes available that might influence their willingness to continue participation in the study;
- The person/s to contact for more information on the study;

3.2.2 Language

- Give information in simple, lay language which is understandable to the patient (and/or parent/guardian/legal representative). Avoid complicated medical terms.
- Use the patient's mother tongue whenever possible.
- Allow sufficient time for the patient (and/or parent/guardian/legal representative) to read the ICF and to ask questions about the study. Address all his/her questions.
- Make sure the patient does not feel obliged to participate in the study.

3.2.3 Illiteracy

- If the patient (and/or parent/guardian/legal representative) is illiterate, an impartial independent witness must be present throughout the entire informed consent discussion.
- Assess on a case-by-case basis, the need for an independent witness, even for patients who have received some degree of formal education.

3.3 Legally incompetent patients

3.3.1 Chronic situations

- For legally incompetent patients (physically or mentally incapable of giving consent, or minors), the consent of a legally authorized representative will be obtained. It is up to the Investigator to verify that the person is legally empowered according to national laws and regulations.
- Whenever possible, for instance in case of adolescents, also the assent from the patient will be obtained, to ensure that he/she is not forced to research participation.

3.3.2 Emergency situations

- In case the patient is not legally incompetent but he/she is admitted in a critical medical condition which impairs his/her ability to decide, the informed consent procedure should not delay care.
- Therefore, under emergency conditions, the consent of a legally authorized representative will be initially obtained. It is up to the Investigator to verify that the person is legally empowered according to national laws and regulations.
- Once the emergency is over and the patient feels better, however, the consent of the patient should be obtained. If the patient does not accept to consent, any information already collected that concerns him/her will be excluded from the study data.

3.4 Signature and documentation

- After explanation and discussion, the patient (and/or parent/guardian/legal representative) should confirm his/her consent in written, by dating and signing the ICF.
- The investigator who conducted the interview, or the qualified person delegated by the investigator for the informed consent procedure who conduct the interview, should also sign and date the ICF.
- If the patient is illiterate, a thumb-print can replace the signature, provided that an impartial witness has been present during the entire informed consent discussion. The impartial witness must also date and sign the ICF. By signing the ICF, the witness attests that the information in the ICF and any other written information was accurately explained to, and apparently understood by the patient and that informed consent was freely given.

- The ICF should be signed in two original copies. One copy will be given to the patient (or parent/guardian/legal representative) and the other will be kept in the Investigator Master File or at another secured location, only accessible to the medical study team.

3.5 After the signature

- It is the investigator's responsibility to ensure that any new information which could affect the patient's willingness to be in the study is timely provided to the patient (and/or parent/guardian/legal representative).
- If during the study, a patient (and/or parent/guardian/legal representative) decides to withdraw his/her consent, he / she is free to do so. Indicate the date/time and the reason for withdrawal in the CRF and other applicable study documents, such as the recruitment log.

4 Storage and access to the ICF

- The signed ICFs must be kept in the Investigator Master File (IMF) and locked in a safe place. The access should be strictly restricted to the study medical staff.
- Do not file the ICF in the Case Report Form (CRF).
- The following persons are allowed to consult the ICF:
    - the site principal investigator
    - the site investigators, clinical officers and nurses
    - the Site Quality Manager
    - the External Monitors
    - the representatives of the Ethic Committees, if required by national regulation
    - the representatives of the regulatory authorities
- The ICF must be kept together with other study documents for at least 10 years after the end of the study.

5 Definitions and abbreviations

5.1 Definitions

- Informed Consent: Informed Consent is the process by which a study participant voluntarily confirms his/her willingness to participate in a study. Only participants who have fully understood all aspects of their participation in the study can make the appropriate judgement and give their consent to participate in the study
- Confidentiality: Maintenance of the privacy of trial subjects including their personal identity and all personal medical information.
- Investigator: Each medical person who is involved in the study conduct, and responsible for the trial and for the rights, health and welfare of the subjects in the trial.
- Impartial witness: a person who is independent form the study, who cannot be unfairly influenced by people involved with the study, who attends the informed consent process if the patient cannot read, and who reads the ICF and any other written information supplied to the patient.

5.2 Abbreviations

- CRF: Case Report Form
- ICF: Informed Consent Form
- IMF: Investigators Master File
- SOP: Standard Operating Procedure

6 Records and archives

**Appendices & Forms for completion**	
**Number**	**Title**
1	Informed Consent Form

7 Document History

Indicate previous versions of the SOP and the changes made

**Revision**	
NA	*NA*

**Name and function**	**Date**	**Signature**
Author		
Veerle Lejon	01 February 2012	
Reviewed by		
Raffaela Ravinetto	01 February 2012	
Approved by		
Emilie Alirol	01 February 2012	
