# Supplementary material for: Experiences and Lessons from a Multicountry NIDIAG Study on Persistent Digestive Disorders in the Tropics
Source: PLoS Negl Trop Dis. 2016 Nov 3;10(11):e0004818. doi: 10.1371/journal.pntd.0004818 (PMC5094778; doi:10.1371/journal.pntd.0004818)
Supplement: S3 Quality SOP — (PDF) [file pntd.0004818.s023.pdf]

nidiag Better Diagnosis of Neglected Infectious Diseases	**SOP Title:** Management of Study Documents
	**Project/study**: This SOP applies to all NIDIAG clinical studies (WP2).

1 Scope and application

This procedure gives a list of essential documents to be collected by the study teams for NIDIAG clinical studies. It also describes how study-related documents should be handled, stored and archived.

2 Responsibilities

Function	Activities
Principal Investigator at each site	- Collect all essential documents pertaining to the work conducted at the study site and file them in the Site Investigator File (SIF)<br>- Ensure the IF is kept up to date<br>- Ensure all essential documents are stored under appropriate conditions and for the required period of time<br>- Make sure access to essential documents is restricted to authorized staff<br>- Make sure that copies of all essential documents originated at the site are sent to the country coordination (except documents baring patients identifiers)
Laboratory head at each site	- Collect all essential documents pertaining to the laboratory work conducted at the study site and file them in the Laboratory File (LF)<br>- Ensure the LF is kept up to date<br>- Ensure all essential documents are stored under appropriate conditions and for the required period of time<br>- Make sure access to essential documents is restricted to authorized staff<br>- Make sure that copies of all essential documents originated at the site laboratory are sent to the country coordination (except documents baring patients identifiers)
Country Coordinating Investigator	- Collect all essential documents pertaining to the work conducted in the country and file them in the Investigator Master File (IMF)<br>- Ensure the IMF is kept up to date<br>- Ensure all essential documents are stored under appropriate conditions and for the required period of time<br>- Make sure access to essential documents is restricted to authorized staff<br>- Make sure that copies of all essential documents originated at the country clinical sites and at the country coordination are shared with the WP2 Task Leader, and that the site receive all the documents which are relevant for their work
WP2 Task Leader	- Collect all essential documents pertaining to the work conducted under the specific task (i.e. Digestive Syndrome, Fever Syndrome or Neurological Syndrome) and file them in the Sponsor Master File (SMF). This responsibility can be delegated, and the delegation must be documented.<br>- Ensure the SMF is kept up to date<br>- Ensure all essential documents are stored under appropriate conditions and for the required period of time<br>- Make sure access to essential documents is restricted to authorized staff<br>- Make sure that copies of all essential documents originated within the country coordinating investigators

Site Quality manager (QM) or equivalent function, and External Monitor	- Verify this SOP is complied with by all intended users and in particular<br>a. verify the IMF, SIF and LF are complete and up-to-date<br>b. verify that access is restricted to authorized individuals<br>- Report all major or systematic deviations from this SOP to the WP2 and WP6 leaders

3 Procedures

3.1 Collection and handling of study essential documents:

A list of all documents to be collected and filed can be found at the end of this SOP. Study documents should be filed in 4 different binders:

- Laboratory File (LF): The Laboratory File includes all essential documents related to the laboratory work at the site level. There is one Laboratory File per study site. The laboratory personnel is responsible for keeping it updated and for ensuring it is stored adequately.
- Site Investigator File (SIF): The Site File includes all essential documents and forms related to the conduct of the study at the site level. There is one Site File per study site. The site investigators are responsible for keeping it updated and for ensuring it is stored adequately.
- Country Investigator's Master File (IMF): The Investigator's Master File includes all essential documents related to the conduct of the study at the country level. There is one Investigator Master File per country. The Country Coordinating Investigator is responsible for keeping it updated and for ensuring it is stored adequately.
- Sponsor Master File (SMF): The Sponsor's Master File includes all essential documents related to the conduct of the study as per ICH GCP guidelines. There is one Sponsor Master File per clinical syndrome. The Task Leader, or the delegated function at his/her institution, is responsible for keeping it updated and for ensuring it is stored adequately.

Refer to Table 1 to know which document needs to be filed in which binder.

File the documents in the order given in Table 1.

3.2 Storage and access to study documents

All documents must be stored in a secured place.

The Laboratory File should be stored in the laboratory. The Site File should be stored under responsibility of the Principal Investigator. The Investigator's Master File should be stored under the responsibility of the Country Coordinating Investigator. The Sponsor File should be stored under the responsibility of the Task Leader.

Access to study documents should be restricted to the staff involved in NIDIAG.

- The access to the Site Investigator File should be restricted to the staff involved in patient's management (study nurses, site investigators, etc…)
- The access to the Laboratory File should be restricted to the lab personnel (Lab technicians, lab head, etc…) and supervisors, including the Country Coordinating Investigator and the quality manager or equivalent function
- The access to the Investigator's Master File should be restricted to the Country Coordinating Investigator and his/her team.
- The access to the Sponsor Master File should be restricted to the Task Leader and his/her team.

The study personnel should prevent accidental destruction of study documents. This involves protection against fire and flood.

Upon request and under confidentiality agreement, site Quality Managers, study monitors, external auditors, inspectors (national regulatory authorities) and Ethics Committee should be given direct access to all study documents.

3.3 Maintenance

- All study documents should be kept up to date. The site principal investigator and the Country Coordinating Investigator should, among others, ensure that the latest versions of the protocol, Informed Consent Form (ICF), Case Report Form (CRF) and Standard Operating Procedures are used. The site investigators are responsible for keeping all documents mentioned in Annex 1 up-to-date and complete.
- The laboratory head should regularly check, among others, that laboratory normal values included in the Laboratory File correspond to the ones used for the analyses, that the study specimen log and records of retained samples are updated in accordance with study recruitment, and that shipping records of biological samples to reference labs are filed. The laboratory head is also responsible for the maintenance of all laboratory documents listed in Annex 1.
- The country coordinating investigator or his/her delegate are responsible for the maintenance of all documents of the Investigators Master File listed in Annex 1. The Country Coordinating Investigator should make sure that changes in essential documents are communicated in a timely fashion to the site principal investigator and to the WP2 Task Leader whenever relevant.
- The Task Leader or his/her delegate should ensure that the content of the Sponsor Master File is regularly updated and matches the current status of the study in each partner country.

All changes occurring during the study should be documented in the SIF, LF (ex: new staff's CV should be collected, new contracts should be established and the organizational diagram of study staff and signature sheet should be updated).

3.4 Archiving

The site principal investigator should keep the Site Investigator File until the Country Coordinating Investigator collects it at the end of the study.

The lab head should keep the Laboratory File until the Country Coordinating Investigator collects them at the end of the study.

The Country Coordinating Investigator should keep the Investigator's Master File and all other study documents for a period of time corresponding to the country's regulatory requirements and for 5 years after the end of the study.

The Task Leader should keep the Sponsor's Master File and all other study documents for a period of time corresponding to the country's regulatory requirements and for at least 5 years after the end of the study.

4 Definitions and abbreviations

<u>Essential Documents:</u> Documents which individually and collectively permit the evaluation of the conduct of a study and quality of the data produced. These documents are important for the successful management of the study. They are also crucial to demonstrate compliance with GCP/GCLP, protocol, SOPs and to enable quality control. A list of essential documents is provided in Annex 1.

<u>Source Documents:</u> These are original documents. They consist of data relevant to the current research such as medical records, administrative files, laboratory reports, consultation reports, pharmacy dispensation registers, etc...

<u>CRF:</u> Case Report Form

<u>ICF</u>: Informed Consent Form

<u>IMF</u>: Investigator Master File

<u>LF:</u> Laboratory File

<u>SIF:</u> Site Investigator File

<u>SMF</u>: Sponsor Master File

<u>SOP</u>: Standard Operating Procedure

5 Records and archives

Appendices & Forms for completion	
**Number**	**Title**
NA	NA

6. Document History

Revision	

Name and function	Date	Signature
Author		
Emilie Alirol	10 May 2012	[illegible]
Reviewed by		
Raffaella Ravinetto	14 May 2012	[illegible]
Approved by		
Rosanna Peeling	24 May 2012	[illegible]

Table 1: List of essential documents to be filed in the SIF,LF

Title of document	SIF	LF	IMF	SMF
**1. Study Documents**				
Signed study protocol and amendments if any	x	x	x	x
Sample Case Report Form (CRF)			x	x
Sample Informed Consent Form (ICF) as well as any written information given to study participants	X		x	x
Study SOPs	x	x	x	x
Nidiag ethical charter	X		X	X
**2. Regulatory Documents**				
All correspondence with ITM IRB and EC of Antwerp University Teaching Hospital (UZA) including final approval of the study protocol and its amendments	x			x
All correspondence with national IRB/EC including final approval of the protocol and its amendments	x		x	x
Approval from the national regulatory authorities (where required)	x		x	x
Interim reports to ECs/IRBs			x	x
Import authorizations for RDTs (if required)			x	x
Insurance statement			x	x
**3. Study Staff and Contracts**				
Delegation log (updated with dates and signatures) of all study staff at the study site, including the lab	x		x	
Delegation log (updated with dates and signatures) of all study staff at the Sponsor level				x
Site contact list	X		x	x
Sponsor contact list (per each protocol)	X		X	X
CVs of all personnel involved in the study (investigators, sub-investigators, lab technicians, etc…)	x		x	x
Signature sheet of all authorized staff at study site, including the lab	x		x	x
GCP/GCLP training certificate of all personnel involved in the study, including the lab	x		x	x
Copy of NIDIAG grant agreement			x	x
Signed agreement between study personnel and partner institution			x	x
4. **Management of RDTs**				
Shipping records of RDTs (including documents for custom clearance)	x		x	x
RDT accountability at study site		x	x	
5. **Biological Samples**				
Country-specific normal values / range for laboratory procedures		x	x	x
Documents pertaining to Quality Control of Laboratory Procedures		x	x	
Shipping records for biological samples (to national and international reference labs)		x	x	
Study specimen logs, including Record of retained biological samples		x	x	
Country-specific sample flow diagram		x	x	x
RDTs results form		x(2)	x(1)	
Completed Temperature Logs		x		
**6. Monitoring**				
Monitoring Plan			x	x
Study Initiation Visit Report or letter of follow up	x		x	x
Monitoring Visit Reports or letter of follow-up				x
Close-out Monitoring Report or letter of follow up				x

**7. Study participants**				
Patient Identification List	x		x	
All signed Informed Consent and Assent forms	x			
Source Documents (including medical files and lab investigations results)	x(2)	x(2)	x(1)	
Signed, dates and completed CRFs	x(1)		x(2)	
**8. Correspondence**				
All essential correspondence between TMG and/or country coordinating investigator and/or the site(s)	x	X	x	x

(1) Copies (2) Originals

SIF: Site Investigator File; LF: Lab File; IMF: Investigator Master File; SMF: Sponsor Master File
