# Supplementary material for: Experiences and Lessons from a Multicountry NIDIAG Study on Persistent Digestive Disorders in the Tropics
Source: PLoS Negl Trop Dis. 2016 Nov 3;10(11):e0004818. doi: 10.1371/journal.pntd.0004818 (PMC5094778; doi:10.1371/journal.pntd.0004818)
Supplement: S4 Quality SOP — (PDF) [file pntd.0004818.s024.pdf]

	**SOP Title:** How to write Standard Operating Procedures (SOPs)
	**Study title**: NA (This SOP applies to all NIDIAG studies)

1. Scope and application

This procedure provides a guideline on how to write a Standard Operating Procedure (SOP), including how to format the document. The purpose of a SOP is to provide detailed instructions on how to carry out a task so that any team member can carry out the task correctly every time. The purpose or objective of a SOP should restate and expand a well-written title. A well-written SOP will facilitate training. The best SOP is one that accurately transfers the relevant information and facilitates compliance with reading and using the SOP. This SOP for SOPs is aimed at WP leaders, task leaders and all those who will be involved in SOP writing. It applies to all SOPs developed within the NIDIAG consortium.

2. Responsibilities

Function	Activities
SOP author	Draft SOP in consultation with the intended users<br>Correct SOP according to WP6 feedback<br>Make SOP available to intended users<br>Amend SOP if required
WP6 representative	Review SOP initial draft and consecutive amendments<br>Release and formally approve SOP versions<br>Make SOP available to the consortium through NIDIAG website
Site Quality Manager and External Monitor	Ensure compliance of SOP by all intended users<br>Ensure the SOP version used is the most recent one approved by WP6<br>Report non compliance to PI and WP6

3. Procedures

3.1 Writing a Standard Operating Procedure (SOP)

- Write one SOP per study-related activity. Ex: Performance of lumbar puncture, Handling, transport and storage of CSF samples, Microscopic detection of trypanosomes etc… Do not mix too many activities in one SOP.
- Make sure you are familiar with the procedure to be described in the SOP. If you are not, ask somebody who performs the procedure regularly to show it to you. Have this person read your first draft before you send it to WP6 for review
- Describe in details how the procedure is being carried out
- List the steps in a chronological order as in the example below

<u>Making a cup of tea:</u>

1. Collect a cup and saucer
2. Place teabag into cup
3. Boil water in kettle
4. Add water to cup and teabag
5. Allow tea to infuse
6. Remove teabag
7. Add milk and sugar (if desired)

- Use a simple, active language e.g. 'weigh 10 mg' rather than '10 mg should be weighed'

- Indicate in the "Responsibilities" section who is doing what. Do not use people's name, use functions / job title e.g. laboratory technician, physician…
- Include all necessary information to perform the procedure, not more.
- Use the fewest possible words, if different steps are involved in the activity, use bullet points
- If possible add visual displays (VD) such as diagrams, flow charts, pictures or table
- Have a specific reader in mind. Know the type of person who will be reading the procedure and tailor the writing according to the end user.
- Avoid "do this or alternatively do that"
- Avoid "where appropriate"
- Make sure all technical terms and acronyms are defined under the "Definition" section

3.2 SOP Format

- Use the SOP template provided by WP6. The template is available on the NIDIAG website (http://www.nidiag.org/)
- Each SOP should have a unique identifier which includes:
  - the number of the Work package under which the SOP is being developed
  - an acronym referring to the type of the procedure (LAB: for laboratory SOP, DOC: for SOP related to documentation management, CLIN: for clinical SOP, DATA: for SOP related to data management, QUAL: for SOP related to Quality Assurance (QA) and Quality Control (QC))
  - the number of the SOP

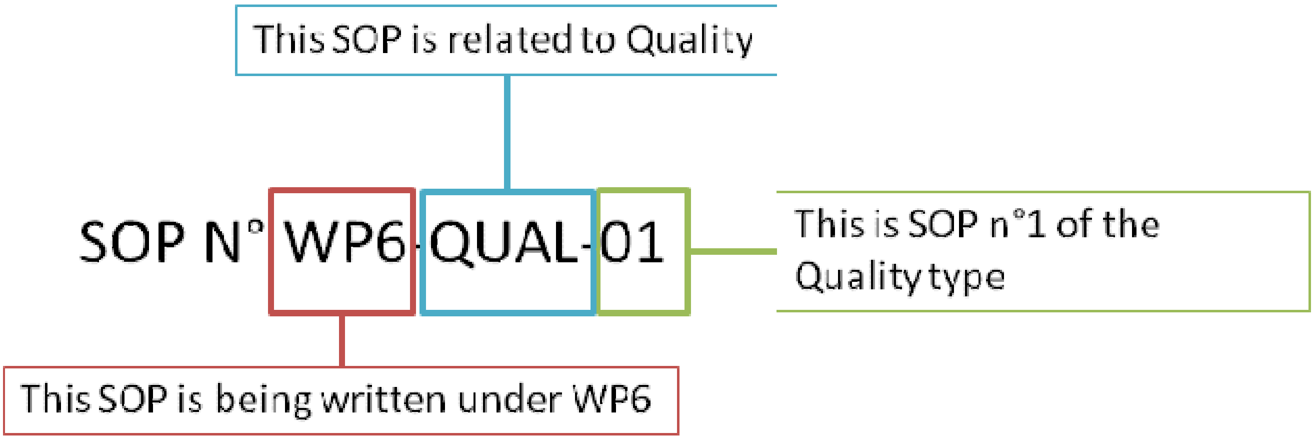

- If the procedure is a lengthy one, then the description of the procedure can be split up and placed under smaller headings. e.g. '3.1 Materials, 3.2 Preparation of reagents, 3.3 Operation and maintenance, …'
- On each page of the SOP indicate:
  - The SOP number, the version number and version date
  - The page number and the total number of pages

3.3 SOPs review and version control

- Each SOP should be reviewed by a WP6 authorized representative. The first draft should be circulated as version 0. Comments and corrections from WP6 should be incorporated in this draft to create version 1. WP6 is responsible for the final approval (final OK) of the document.
- The SOP should be signed by the SOP author and by the person who reviews and approves it.

- Corrections and modifications of the initial version should also be submitted for review to WP6. Version 2 should be created only after WP6 feedback and approval has been obtained.
- Each consecutive version and reason for/description of each modification made to the SOP should appear in the "Document History" section at the end of the SOP.

3.4 SOPs language

SOPs will be written in English. However, SOPs intended to French-speaking users will be translated to French.

4. Definitions

Principal Investigator (PI): Person who is responsible for the overall conduct of the study at a given site. The principal investigator is the responsible leader of the team involved in NIDIAG.

Standard Operating Procedures (SOPs): SOPs are issued to specifically instruct employees / team members in areas of responsibility, Work Instructions, appropriate specifications and required records. SOPs outline procedures, which must be followed to claim compliance with GCP and GCLP principles or other Statutory rules and regulations. Procedures can take the form of a narrative, a flow chart, a process map, computer screen printouts or combination of all or any other suitable form, however must be written in appropriate, effective grammatical style. (e.g. plain English).

Form: A form is a document which is to be printed at the time of use and filled out for the purpose of becoming a record (e.g. Library Log Form), or for the purpose of becoming Visual Display tool.

Template: A template is a form to be used as a model for creating other documentation

Visual Display (VD): A VD is a form requiring no additional data to be added, (i.e. no written record) which provides visual information to instruct in the process, e.g. "Out of Order" tag stuck on a machine. The information can be in the form of pictures or photographs; flowchart; operating instructions; or a notice. The Visual Display form is usually located in a permanent position, however maybe in use for a specific period of time, e.g. for a single batch. Pages from a single Visual Display form must be located together in a specified location.

5. Records and archives

Appendices & Forms for completion	
**Number**	**Title**
NA	*NA*

6. Document History

**Revision**	
-	-

**Name and function**	**Date**	**Signature**
Author		
Emilie Alirol	24.10.2011	
Reviewed by		
Raffaella Ravinetto<br>Rosanna Peeling	27.10.2011	
Approved by		
Veerle Lejon	07.11.2011	
