# Supplementary material for: Experiences and Lessons from a Multicountry NIDIAG Study on Persistent Digestive Disorders in the Tropics
Source: PLoS Negl Trop Dis. 2016 Nov 3;10(11):e0004818. doi: 10.1371/journal.pntd.0004818 (PMC5094778; doi:10.1371/journal.pntd.0004818)
Supplement: S5 Quality SOP — (PDF) [file pntd.0004818.s025.pdf]

	**SOP Title:** External monitoring
	Project/study: this SOP applies to all NIDIAG clinical studies (WP2).

1. Scope and application

Monitoring visits are conducted throughout NIDIAG clinical studies to ensure that:

1) the rights and well-being of research subjects are protected
2) the study is conducted in accordance with GCP and regulatory requirements and with the study protocol
3) study data are accurate, complete and verifiable from source documents.

This procedure describes how external monitoring visits should be carried out, documented and followed up, in compliance with WHO and ICH Good Clinical Practices Principles.

2. Responsibilities

Function	Activities
External Monitor	- Plan monitoring visits in accordance with the monitoring plan, as agreed with the WP6 leader<br>- Verify that the trial is conducted in compliance with international and national regulatory/ethical requirements, with the study protocol and amendments, and with the NIDIAG SOPs<br>- Report any deviations in the monitoring visit report<br>- Send the monitoring visit report to the concerned WP6 leader
Quality manager (QM) or equivalent function	- Support the external monitor in the fulfillment of his/her tasks<br>- Attend monitoring visits
Site PI	- Support the external monitor in the fulfillment of his/her tasks<br>- Attend monitoring visits and ensure all key-study staff are present<br>- Ensure that the external monitor has full access to the study documents and facilities<br>- Ensure that the corrective actions listed in the monitoring visit report and/or follow-up letter are implemented
Country coordinating investigator or his/her delegate<br><br>WP2 leader or his/her delegate	- Verify that the corrective actions listed in the monitoring visit report and/or follow-up letter are implemented (joint follow-up will take place within the clinical trial-specific Trial Management Groups)
WP6 leader or his/her delegate	- Be the link between the Trial Management Group and the monitor<br>- Establish monitoring plan for each study site<br>- Ensure that the Monitor receives any new key-information relevant to the study status and conduct<br>- Review the monitoring visit reports and/or the follow-up letter.<br>- Send timely a copy of the monitoring visit report and/or follow-up letter to WP2 leader or his/her delegate, the Country coordinating investigator, and to the concerned members of the Trial Management Group<br>- Send a copy of the monitoring visit report and/or follow-up letter to the site PI

3. Procedures

3.1 Monitoring schedule and visit planning

The WP6 leader, in consultation with the Country Coordinating Investigator, the WP2 leader and other concerned members of the Trial Management Group, will determine the appropriate calendar of monitoring visits for each clinical study l (e.g. country monitoring plan).

The monitor is responsible for planning monitoring visits in accordance with the monitoring plan.

Prior to visit, the monitor should:

- Review the study protocol, CRF, monitoring tools (Monitoring Visit Report and/or Follow-up Letters, NIDIAG SOPs…) and previous reports, as well as any other key documents provided by the WP6 leader.
- Inform the WP6 leader and the site PI at least 2 weeks ahead of the monitoring visit, and ask them to ensure that all key-staff are present for the visit.
- Send a provisional visit agenda, agreed with the WP6 leader, to the site PI.
- Discuss with the WP6 leader about the latest developments of the study and agree on communication streamlines, so that the WP6 leader can contact the Monitor if needed for the follow up of the visit's findings.
- Enquire about the number of patients included in the study site since last visit. Make sure that enough time is allowed during the visit to complete all monitoring activities.

3.2 Study initiation visit

It is performed after receipt of all study materials, after approval of the EC and CA, and prior to the start of recruitment. At least the following activities must be carried out:

Ethical and GCP training: the Monitor should meet the site PI and study staff, to verify that they have an appropriate understanding of ethical and GCP requirements. If needed, he/she should conduct a GCP training or refreshment session.

Protocol and CRF review: the Monitor will review the protocol and the CRF with all the Investigators and co-workers.

Site Investigator's file and Laboratory File: the Monitor should verify the completeness of the Site Investigator's file and Laboratory File (ref. SOP No WP6-DOC-03 on Management of Study Documents). If logistically feasible and if time allows it, the Monitor should also check the completeness of the Investigator Master File.

Visit of site's facilities: the Monitor should visit the site's facilities, including the laboratory, to confirm that they are adequate for the conduct of study activities. He/she should also check that there are appropriate spaces and facilities for storage of the study materials and for archiving the site investigator's file and source documents.

Reporting: the Monitor will document all the above in a Monitoring Visit Report, which will be sent to the WP6 leader/delegate. It is the responsibility of the WP6 leader to timely distribute it to the country coordinating investigator/delegate, to the WP2 leader/delegate, to other concerned members of the Trial Management Group and to the site Principal Investigator for follow up on findings and corrective actions.

Exceptions: the WP6 leader may decide to skip the study initiation visit, if all the points have been carried out and documented on previous visits, and corrective actions have been implemented.

3.3. Routine Monitoring visits

The first monitoring visit takes place as early as possible, and no later than one month after recruitment started, so that any major mistakes or systematic problems may be identified and corrected in the early phases. At least the following activities should be carried out:

Follow up of previous visit: the Monitor will follow-up any pendings from the previous visit/s.

New staff: if new staff have been appointed, the Monitor will check the CV, delegation log and training records. If needed, he/she will carry out a GCP training/refreshment session.

Progress of the trial: the Monitor will discuss the recruitment status (planned vs. achieved) with the Site Principal Investigator. He/she will also check if the patients' referral process is working satisfactorily.

Informed consent: the Monitor will check informed consent has been obtained and properly documented for each patient prior to undergoing any Study-specific procedures (ref SOP No WP6-DOC-01 on Informed Consent).

Compliance with the protocol: the Monitor will verify the overall compliance with the protocol and amendments. Any serious and/or any systematic deviations will be brought to the attention of the country coordinating investigator and WP2 leader. Corrective action must be planned.

CRF: the Monitor will provide guidance and support on the correct procedures for filling the CRF and (when appropriate) for performing data entry.

Source data verification The Monitor will review the patients' files, to verify that the information entered in the CRF match the original observations in the hospital files and in the laboratory print-outs, and that it is consistent and accurate. The SDV is carried out on a sample basis (e.g., on 15% of all CRF) and percentage must be increased if the findings are not satisfactory.

Site Investigator's file and Laboratory file: the Monitor will verify completeness of the Investigator's study file and of the Laboratory file (ref. SOP No WP6-DOC-03 on Management of Study Documents)

Rapid diagnostic tests: the Monitor will check that the storage, transportation and dispensing conditions are appropriate, and will check that the expiry dates have not been exceeded. He/she will review the drug accountability forms.

Reporting: the Monitor will document all the above in a Monitoring Visit Report, which will be sent to the WP6 leader/delegate. It is the responsibility of the WP6 leader to timely distribute it to the country coordinating investigator/delegate, to the WP2 leader/delegate, to other concerned members of the Trial Management Group, and to the site Principal Investigator for follow up on findings and corrective actions (the WP6 leader may choose to send to the PI either a follow-up letter summarizing the main findings and corrective actions needed, or a copy of the Visit Report itself).

3.4. Close-out visit

It is performed after the last visit has been completed, all data have been entered in the database and all queries resolved. At least the following activities should be carried out:

Follow up of previous visit: the Monitor will follow-up all pendings from the previous visits. A plan of action will be agreed for corrective actions that cannot be implemented during the visit.

Final trial assessment: the final number of patients screened, recruited, drop-out and completed, must be verified and attached to the close-out monitoring visit report.

Unused study rapid diagnostic tests: the Monitor will check the accountability forms and will perform a final inventory, to check that there are no discrepancies. The tests that are registered in the country can be given to the Hospital Pharmacy. For non-registered products, arrangements will be defined by the WP6 leader.

Site Investigator's file: the Monitor will verify completeness of the Site Investigator's file, and will check that it is moved to the place of the country coordinating investigator, to be available for audits or inspections (ref. SOP No WP6-DOC-03 on Management of Study Documents).

Reporting: the Monitor will document all the above in a Close-Out Visit Report, which will be sent to the WP6 leader/delegate. It is the responsibility of the WP6 leader to timely distribute it to the country coordinating investigator/delegate, to the WP2 leader/delegate, to other concerned members of the Trial Management Group and to the site Principal Investigator for follow up on findings and corrective actions (the WP6 may choose to send to the Principal Investigator either a follow-up letter summarizing the main findings and corrective actions needed, or a copy of the Visit Report itself).

Exceptions: the WP6 leader may decide to skip the close-out visit, provided that all the above points have been carried out and documented on previous visits (e.g., by the laboratory supervisor) and that corrective actions have been implemented.

3.5. Note on laboratory supervision

Most clinical monitors do not have a specific laboratory expertise. Because of the characteristics of the Nidiag project, external clinical monitoring must be complemented by regular supervision visits carried out by the lab experts.

4. Definitions and abbreviations

Audit A systematic examination, carried out independently of those directly involved in the clinical trial, to determine whether the conduct of the trial complies with the protocol and whether the data reported are consistent with the records on site.

Case-Report Form A document used to record data on each trial subject during the trial, as defined by the protocol. The data should be collected by procedures which guarantee preservation, retention and retrieval of information and allow easy access for verification, audit and inspection.

Compliance Adherence to all the research related requirements, Good Clinical Practice requirements, and the applicable regulatory requirements.

Good Clinical Practice A standard for clinical studies which encompasses the design, conduct, monitoring, termination, audit, analyses, reporting and documentation of the studies and which ensures that the studies are scientifically and ethically sound and that the clinical properties of the pharmaceutical product (diagnostic, therapeutic or prophylactic) under investigation are properly documented.

Informed consent A subject's voluntary confirmation of willingness to participate in a particular trial, and the documentation thereof. This consent should only be sought after all appropriate information has been given about the trial including an explanation of its status as research, its objectives, potential benefits, risks and inconveniences, alternative treatment that may be available, and of the subject's rights and responsibilities in accordance with the current revision of the Declaration of Helsinki.

Inspection An officially-conducted examination by relevant authorities at the site of investigation and/or at the site of the sponsor in order to verify adherence to Good Clinical Practice.

Investigator Each medical person who is at some stage involved in the study conduct, and responsible for the trial and for the rights, health and welfare of the subjects in the trial. The investigator should have qualifications and competence in accordance with local laws and regulations as evidenced by up-to-date curriculum vitae and other credentials.

Monitor A person appointed by, and responsible to, the sponsor for the monitoring and reporting of progress of the trial and for verification of data.

Patient/subject file A collection of data consisting of all relevant information on the patient or subject (such as hospital file, consultation records or special subject file) that permits the authenticity of the information presented in Case-Report Forms to be verified and, where necessary, completed or corrected.

Principal investigator The investigator serving as coordinator within each study site.

Protocol A document which states the background, rationale and objectives of the trial and describes its design, methodology and organization, including statistical considerations, and the conditions under which it is to be performed and managed. The protocol should be dated and signed by the investigator, the institution involved and the sponsor.

Protocol amendment A written description of a change to or a formal clarification of the protocol.

Source data All records or certified copies of original observations, clinical findings or other activities in a clinical trial necessary for the reconstruction and evaluation of the trial. Such material includes laboratory notes, memoranda, calculations and documents, as well as all records of data from automated instruments or exact, verified copies in the form of photocopies,

microfiches etc. Raw data can also include photographic negatives, microfilm or magnetic media (e.g. computer diskettes).

Source data verification The procedures carried out to ensure that the data contained in the final study report match original observations. These procedures may apply to raw data, data in Case-Report Forms (in hard copy or electronic form), computer print-outs and statistical analyses and tables.

Trial Management Group The entire group of individuals and functions that are jointly responsible for the appropriate set up, conduct and daily management of the clinical trial in WP2.

AE	Adverse Event
CA	Competent Authorities
CRF	Case-Report Form
CV	Curriculum Vitae
EC	Ethics Committee
GCP	Good Clinical Practice
ICH	International Conference for Harmonisation
SDV	Source Data Verification
SOP	Standard Operating Procedure
TMG	Trial Management Group
WHO	World Health Organisation

5. Records and archives

Write in this section the records that need to be filled when following the procedure, and where these records will be stored and eventually archived. This can include a lab book for example, or a checklist,

**Appendices & Forms for completion**	
**Number**	**Title**
1	Monitoring visit report

6. Document History

**Revision**	
V.2	Compared to the first version of this SOP (dated 7 Jul 2012), the WP6 now becomes the central contact for the external monitor, in compliance with the ICH GCP (the WP6 represents the sponsor).

**Name and function**	**Date**	**Signature**
Author		
Raffaella Ravinetto	5th December 2012	
Reviewed by		
Emilie Alirol	5th December 2012	
Approved by		
Rosanna Peeling	5th December 2012	
