# Supplementary material for: Experiences and Lessons from a Multicountry NIDIAG Study on Persistent Digestive Disorders in the Tropics
Source: PLoS Negl Trop Dis. 2016 Nov 3;10(11):e0004818. doi: 10.1371/journal.pntd.0004818 (PMC5094778; doi:10.1371/journal.pntd.0004818)
Supplement: S6 Quality SOP — (PDF) [file pntd.0004818.s026.pdf]

	**SOP Title:** Internal Quality Control Activities
	Project/study: This SOP applies to all NIDIAG studies.

1. Scope and application

All NIDIAG studies have to be conducted in compliance with the NIDIAG Ethical Charter, the national and international applicable regulations, the WHO Good Clinical Laboratory Practices (GCLP) and the ICH and WHO Good Clinical Practices (GCP) Guidelines. The GCP/GCLP monitoring system to be implemented throughout NIDIAG includes 2 complementary components: 1) An internal quality control (IQC) component and 2) an external quality control (EQC) component which includes lab monitoring. The IQC component involves site Quality Managers (QM) who are responsible for performing regular quality checks at the study site.
Since the GCP and GCLP supervision may entail different skills and expertise, the site country coordinating investigator can decide to allocate the task of QM to one or two persons within his/her team, depending on available skills and expertise.
This procedure describes the responsibilities of the site QM and how his/her work articulates with the work of the external monitoring component (SOP-WP6-QUAL-02) and of lab monitoring.
This SOP doesn't apply to DRC.

2. Responsibilities

**Function**	**Activities**
Site Quality manager (QM)	- Ensure the study site team is aware of all aspects related to GCP/GCLP compliance<br>- Ensure the study site team is trained on the study protocol and relevant NIDIAG SOPs<br>- Conduct regular quality control checks for all study-related activities<br>- Document the quality control checks in the QM Report and discuss the GCP/GCLP deviations identified with the PI and the study team<br>- Report any major deviation –in addition to the site PI- to the WP6 leader, who will coordinate communication with the External Monitor and/or the lab monitor, as needed<br>- Support the PI, the External Monitor and the lab supervisor in the organization and conduct of monitoring visits (SOP-WP6-QUAL-02).<br>- Attend monitoring visits<br>- Assist Site Principal Investigator in the implementation of corrective actions following IQC and EQC
External Monitor<br>Lab Monitor	- Provide advice and support to QM on the performance of quality control checks between monitoring visits<br>- Review any major deviation reported by the QM and if required, discuss these deviations with WP6 leader
Site Principal Investigator (PI)	- Ensure that the QM has full access to the study documents and facilities<br>- Ensure that the QM is allocated enough time to carry out the requested activities, without any detriment for his/her other routine tasks<br>- Ensure that the QM receives any new key-information relevant to the study status and conduct Implement corrective actions to address the deviations identified by the QM
Country Coordinating Investigator	- Assign the task of QM to one or two member(s)

WP6 leader or his/her delegate	- Be the link between the Trial Management Group and the QM<br>- Provide guidance to QM for the planning of QC activities at each study site<br>- Review the QM reports and timely send a copy to the Country Coordinating Investigator, the WP4 representative and the concerned members of the Trial Management Group<br>- Be in touch with the QM and PI regularly, to advise them on problems concerning the quality and/or the frequency of QC

3. Procedures

3.1 Training of the study site team

All staff involved in NIDIAG should receive training on the study protocol, the SOPs that are relevant to his/her work, Good Clinical Practice (GCP) and Good Clinical Laboratory Practice (GCLP). The QM will, together with the site PI and the external monitor/lab monitor, provide such training for all staff who have not attended NIDIAG GCP/GCLP workshop, before recruitment starts. The model training material is available through the NIDIAG website (www.nidiag.org) or through WP6 leader.

The QM together with the PI will provide training for new staff in case of handovers, or retraining to staff in need of it.

3.2 Description of Internal Quality Control Activities

3.2.1. Verifying Informed Consent Forms (ICF)

Verify compliance with NIDIAG SOP-WP6-DOC-01. In particular, the QM should try to be present during the informed consent discussion for the first 10 patients enrolled in the study and then after, at least once every 25 patients, provided of course the individual patients agree. He/she should verify that the process of obtaining Informed Consent complies with the principles of the Helsinki Declaration, WHO and ICH Guidelines for Good Clinical Practice.

Verify that **all** patients included in the study have provided written informed consent:

- Verify that names, date and time of ICF signature are appropriate.
- Check whether or not a legal representative /an independent witness was required for each one of the cases according to GCP and SOP-WP6-DOC-01
- For legally incompetent subjects, check whether assent was required and if yes, if it was obtained
- For patients included under an emergency situation, check that consent was initially obtained from the patient's relatives and then reiterated by the patient himself/herself
- Verify that consent has been obtained prior to any study related procedure
- Verify with the investigator that each participant has been given a signed copy of the ICF
- Verify that all consent forms are securely stored in the Site Investigator's File

Document the ICF review in the Quality Manager report.

3.2.2. Reviewing Case Report Forms (CRF) and Lab Data Collection Forms

Check that the "Patient Identification list" is completed, accurate and coherent with the center's register (check patient's name, initials, date of birth/age, registration number, date of admission).

Check that patients' numbers have been allocated in accordance with SOP-WP6-DOC-02.

Note down the number of screened patients and the number of enrolled patients in the QM report.

For all patients, check that CRF have been completed in accordance with SOP-WP6-DATA-01. Check that CRF entries are legible and that only authorized staff have filled in the CRFs and laboratory data collection forms.

Check all patient's CRF against source documents, i.e. the patient medical file, the laboratory notebook(s), the study doctor's notes, the study center's register and any other source document available at each site.

In particular check the following:

- The results of the reference tests performed on site are consistent throughout the CRF, the lab notebook(s) and other lab report forms
- The results of the reference tests performed in reference labs are consistent throughout the CRF and the reference lab report forms
- The results of the index tests are consistent throughout the CRF, the lab notebook(s) and any other source document available at each site (if applicable).
- The medication prescribed are consistent throughout the CRF, the patient medical file and the study doctor's notes
- The timing of clinical examinations, sample collection and laboratory investigations are consistent throughout all documents

Regarding Index test results the source is the lab register / lab notebook in Cambodia, Nepal and Sudan.

In case of discrepancies between the CRF and the source documents, report them in the QM report. Remind the PI to ensure missing data is completed and that incorrect entries are corrected by the responsible site investigator.

Check that the format of corrections made in CRF and other data collection forms complies with SOP-WP6-DATA-01.

3.2.3. Reviewing the Site Investigator File/Lab File

The Site Investigator File includes all essential documents to be collected and filed by the study team throughout the NIDIAG project.

The lab file includes all essential lab documents to be collected and filed by the lab team.

Support the PI and the lab manager to check that the SIF and the lab file are complete and kept up-to-date in accordance with SOP-WP6-DOC-03.

Verify that the site investigator sends copies of relevant documents to the Country Coordinating Investigator.

3.2.4. Biological Samples

All laboratory activities should be verified by a QM with appropriate laboratory skills.

Some of the samples collected during NIDIAG are processed and analyzed on site. Some others are sent to reference laboratories (in the country or abroad), while others are stored in view of future research.

Check that the sample flow is running smoothly and in accordance with the corresponding SOP.

Verify that the handling, processing, management and disposal of hazardous specimens meet the necessary safety precautions.

Check that the "Study Specimen Log" or equivalent document(s) is completed, accurate and coherent with the center's register and the CRF (check patient's number, date of specimen collection, type of specimen collected, whether the specimen was shipped or not, etc…).

Check that the samples are correctly numbered and labeled in accordance with SOP-WP6-DOC-02. Verify that samples are not labeled with patient's personal identifiers (names, address, telephone number).

Check that samples are appropriately stored, and in particular that:

- access is restricted to authorized staff
- study specimens are stored separately from specimens collected for other lab routine activities
- the storage temperature is appropriate according to relevant SOPs and checked regularly by responsible staff

Check that analyses performed on site are conducted in accordance with the protocol and applicable NIDIAG SOPs. Verify that:

- analyses are performed only by appropriately trained staff
- low-quality samples (i.e. insufficient volume collected, contaminated samples, etc…) are rejected upon receipt

- deviations from SOPs are documented in the lab notebook(s)
- analyses yielding abnormal test results or out-of-range /invalid controls are repeated
- results are validated by lab director before being transmitted to the site PI
- blinding is respected for index tests
- results are transmitted in a timely fashion to the site PI

Check that the shipment of samples to reference laboratories (inside or outside the country) is appropriate: verify that the timing of shipment complies with the applicable NIDIAG SOP, that the temperature during transport is adequate and that the necessary safety precautions are taken for hazardous samples. Also, verify that the date and time of specimen shipment, the duration of transport and the date and time of specimen receipt at the reference lab is documented in the “Study Specimen Log”.

3.2.5. Index RDTs storage and accountability

Verify that the index RDTs evaluated in NIDIAG studies are stored in appropriate manner. In particular, check that:

- Access to RDTs stored is restricted to authorized staff
- The storage temperature is checked regularly by responsible staff. RDT kits should not be used if they are damaged.

Also verify that:

- RDTs are used before the expiry dates
- RDTs are used only for patients included in the study
- An adequate system for RDT accountability is in place

3.3 Frequency of Quality Control activities

Quality control activities should be performed regularly throughout recruitment, on at least every 25 patients enrolled in the study (see Table 1).

Quality control activities should be performed prior to routine monitoring visits. The PI is responsible for ensuring that the study team implements corrective actions formulated by the QM and address deviations BEFORE the external monitor visit.

It is anticipated that internal quality control activities, if conducted regularly as indicated below, should take approximately 3 days to complete.

Table 1: Suggested frequency of internal quality control activities

Internal QC activity	Frequency of QC activity
Verification of ICF	ALL patients, every 10 patients enrolled
Verification of Patient ID list	ALL patients, at least every 25 patients enrolled
Review of CRF	ALL patients, at least every 25 patients enrolled
Review of SIF, IMF and LF	At least once in a month and every time there is major change (i.e. change in staff, amendment of study protocol, receipt of new RDTs batches)
Verification of biological samples	ALL patients, at least every 25 patients
Verification of index test storage and accountability	At least once in a month and upon receipt of a new RDT package

3.4 Reporting

Each time quality control activities are conducted, a QM report should be completed and shared and discussed with the site PI and the WP6 leader and/or his delegates.

All critical and major deviations should be immediately reported (in addition to the site PI) to the WP6 leader or delegate, who will circulate them to the relevant persons in the TMG, to help formulating corrective actions.

QM are encouraged to group internal QC activities over a period of 2-3 days and to adapt the frequency of the quality checks based on recruitment pace and problems encountered. As long as possible, QM should submit complete reports to the WP6 representative, although some activities may not be conducted with the same frequency.

4. Definitions and Abbreviations

4.1 Definitions

Laboratory File (LF): The Laboratory File includes all essential documents related to the laboratory work at the site level. There is one Laboratory File per study site. The laboratory personnel is responsible for keeping it updated and for ensuring it is stored adequately.

Site Investigator File (SIF): The Site File includes all essential documents and forms related to the conduct of the study at the site level. There is one Site File per study site. The site investigators are responsible for keeping it updated and for ensuring it is stored adequately.

Country Investigator's Master File (IMF): The Investigator's Master File includes all essential documents related to the conduct of the study at the country level. There is one Investigator Master File per country. The Country Coordinating Investigator is responsible for keeping it updated and for ensuring it is stored adequately.

Critical deviation: Conditions, practices or processes that adversely affect the rights, safety or wellbeing of the subjects and/or the quality and integrity of data. These must be documented and reflected in the yearly and final study reports. Corrective actions must be immediately implemented.

Major deviation: Conditions, practices or processes that might adversely affect the rights, safety or wellbeing of the subjects and/or the quality and integrity of data. These must be documented and, if needed, reflected in the yearly and final study reports. Corrective actions must be implemented.

Quality Assurance (QA): All those planned and systematic actions that are established to ensure that the study is performed and the data are generated, documented (recorded), and reported in compliance with Good Clinical Practice (GCP) and the applicable regulatory requirement(s).

Quality Control (QC): The operational techniques and activities undertaken within the quality assurance system to verify that the requirements for quality of the trial-related activities have been fulfilled.

Source Document: These are original documents. They consist of data relevant to the current research such as medical records, administrative files, laboratory reports, consultation reports, pharmacy dispensation registers, etc...

4.2 Abbreviations

CRF: Case Report Form

EQC: External Quality Control

GCLP: Good Clinical Laboratory Practice

GCP: Good Clinical Practice

ICF: Informed Consent Form

IMF: Country Investigator File

IQC: Internal Quality Control

LF: Laboratory File

PI: Principal Investigator

RDT: Rapid Diagnostic Test

QM: Quality Manager

SIF: Site Investigator File

**Revision**	
V.3	Project/study concerned was changed from “Fever study” to “All studies” since Neuro and Digestive syndromes are also concerned by this SOP.
V.2	Compared to the first version of this SOP (dated 6 Aug 2012), the WP6 now becomes the central contact for the quality manager, and the external lab monitor is regularly mentioned when applicable.<br>Also, the extent and frequency of quality control activities was further described

5. Records and archives

**Appendices & Forms for completion**	
**Number**	**Title**
SOP-WP6-QUAL03-annex1	Quality Manager Report Template

6. Document History

**Name and function**	**Date**	**Signature**
Author		
Emilie Alirol	01 February 2013	
Reviewed by		
Raffaella Ravinetto<br><br>Rosanna Peeling	01 February 2013<br><br>01 February 2013	
Approved by		
Ninon Horié	17 March 2014	
