# Supplementary material for: Experiences and Lessons from a Multicountry NIDIAG Study on Persistent Digestive Disorders in the Tropics
Source: PLoS Negl Trop Dis. 2016 Nov 3;10(11):e0004818. doi: 10.1371/journal.pntd.0004818 (PMC5094778; doi:10.1371/journal.pntd.0004818)
Supplement: S7 Quality SOP — (PDF) [file pntd.0004818.s027.pdf]

nidiag Better Diagnosis of Neglected Infectious Diseases	**SOP Title: Laboratory (GCLP) supervision visits**
	Project/study: NIDIAG: this SOP applies to all NIDIAG clinical studies (WP2).

1. Scope and application

All NIDIAG studies have to be conducted in compliance with the NIDIAG Ethical Charter, the national and international applicable regulations, the WHO Good Clinical Laboratory Practices (GCLP) and the ICH and WHO Good Clinical Practices (GCP) Guidelines. The GCP/GCLP monitoring system to be implemented throughout NIDIAG includes 2 complementary components: 1) An internal quality control (IQC) component and 2) an external quality control (EQC) component.
This procedure describes how laboratory supervision visits should be carried out, documented and followed up, in compliance with the GCLP principles.

2. Responsibilities

Function	Activities
Laboratory supervisor	- Verifies that the trial is conducted in compliance with the GCLP requirements, with the study protocol and amendments, and with the NIDIAG SOPs<br>- Reports any deviation in the laboratory visit report<br>- Sends the laboratory visit report at a minimum to the WP2 leader, the country coordinator investigator, the site principal investigator and the WP6 monitoring coordinator*<br>- Address specific questions in-between two visits
External Monitor	- Reports any deviations regarding the laboratory in the monitoring visit report
Quality manager (QM) or equivalent function	- Supports the laboratory monitor in the fulfillment of his/her tasks<br>- Attends the laboratory monitoring visits
Site Principal Investigator (PI)	- Supports the laboratory monitor in the fulfillment of his/her tasks<br>- Attends the laboratory monitoring visits and ensures all key-study staff are present<br>- Ensures that the laboratory monitor has full access to the study documents and facilities<br>- Ensures that the corrective actions listed in the laboratory visit report and/or follow-up letter are implemented
Country coordinating investigator	- Ensures that the laboratory monitor receives any new key-information relevant to the study status and conduct<br>- Verifies that the corrective actions listed in the laboratory visit report and/or follow-up letter are implemented
WP6 monitoring coordinator*	- Makes sure that the laboratory monitor receives the reports from the external monitor

*The WP6 monitoring coordinator is Raffaella Ravinetto for Neurological syndrome, Ninon Horié for Fever syndrome and Peiling Yap for Digestive syndrome.

3. Laboratory supervision visits to be carried out

3.1 Laboratory supervision visit planning

The WP6 coordinator, in consultation with the Country Coordinating Investigator and the WP2 leader, will determine the appropriate calendar for laboratory monitoring visits for each clinical study. The laboratory supervision visits take place at the field site itself and at the referral labs and central administration, if applicable.

Prior to visit, the laboratory supervisor should:

- Review the study protocol, CRF, study SOPs and previous reports from external monitor/laboratory supervisor, as well as any other key documents provided by the country coordinating Investigator or by the WP2 leader.
- Inform the country coordinating investigator and the site PI at least 1 month ahead of the laboratory supervision visit, and ask them to ensure that all key-staff are present for the visit.
- Send a provisional visit agenda to the country coordinating investigator.

1.2 Laboratory supervision visits to be carried out

1. Laboratory assessment visit

The laboratory assessment is carried out before start of recruitment. It is carried out by the WP2 leader or a qualified delegated person, together with the country coordinating investigator, and ideally with either the laboratory supervisor (if already identified) or a delegate with knowledge on routine laboratory procedures and GCLP aspects. A report with the outcomes and conclusions from the assessment is prepared and distributed among the study coordination team.

During the laboratory assessment visit, the following aspects are checked (checklists can be used as an aid):

- The general conditions of the laboratory (facilities, electricity supply, which kind of laboratory testing is performed, type and quality of reagents and equipment, stock system, laboratory recording, etc.)
- The availability and knowledge of the lab staff (sufficient dedicated number of lab staff available, lab staff competent or not, back-up available or not)
- The quality management system for the lab (quality controls available, participation in EQA, documentation, etc.)

The following decisions and actions should be taken before study initiation visit:

- Decide which (index) testing should be performed on site and which should be performed in referral labs and make sure that all (index) tests are validated before use.
- Develop an analytical plan or alternatively, make sure that this type of information is available through other means (SOPs, lab file), *e.g.* sample and result flow chart, SOP on handling, storing and management of study samples; documentation of normal ranges, SOP on data management, …
- Make sure a high quality Case Report File (CRF) is developed in collaboration with WP6 data manager and statisticians.
- Make sure that all necessary equipment, test kits and reagents for all laboratory tests to be performed are available.
- Make sure SOPs are available for all laboratory procedures and related activities (*e.g.* quality assurance, data management). If needed, prepare site specific SOPs.
- Make sure an efficient ordering system is in place for a timely and routinely supply of test kits and reagents (either locally or centrally at the study coordinator site[1])

If any of the above requirements are not fulfilled, the study should NOT be initiated.

2. Study initiation visit

The study initiation visit is performed after receipt of all study materials, after approval of the EC, and prior to the start of recruitment. Ideally the visit is carried out together with the external monitor and/or WP2 leader. At least the following activities must be carried out:

- *GCLP training*[2]:

  In discussion with the WP6 monitoring coordinator and the monitor of the site, a formal GCLP training is given for all staff who have not yet attended NIDIAG GCP/GCLP workshop, by either the external monitor and/or the laboratory supervisor in collaboration with the QM, before start of recruitment.

- *Protocol review*: the laboratory aspects of the protocol are reviewed with all the study staff, the analytical plan and related documents are discussed (*e.g.* sample and result flow chart).
- *Laboratory training:* If required training is provided on all the SOPs related to laboratory activities (laboratory procedures, handling/storing/management of study samples, source data and data management, stock system, quality assurance, etc.)
- *CRF training:* Training on the laboratory part of the Case Report File (CRF) is provided.
- *Laboratory File*: The laboratory file is verified for completeness (ref. SOP No WP6-DOC-03 on Management of Study Documents).
- *Laboratory facility*: the laboratory supervisor should confirm that the lab is adequate for the conduct of study activities (storage space for lab materials and source documents, temperature monitoring).
- *Piloting*: If all findings are satisfactory, a piloting study (with 10-20 patients) can be conducted at the end of the initiation visit, under supervision of the laboratory supervisor and/or the external monitor/WP2 leader. During this piloting study, all activities are carried out as if real patients were recruited. Corrective actions are applied where needed.
- *Start of recruitment:* If the piloting phase was satisfactory, the recruitment can start.

A laboratory supervision report of the initiation visit and the piloting phase is prepared, documenting all the above, which is sent to the WP2 leader/delegate, the country coordinating investigator/delegate, and to the site Principal Investigator for follow-up on findings and corrective actions.

Exceptions: the WP2 leader may decide to skip the study initiation visit, if all the points have been carried out and documented on previous visits, and corrective actions have been implemented.

3. <u>Routine laboratory supervision visits</u>

Routine laboratory supervision visits take place as planned with the WP6 monitoring coordinator and the country coordinating investigator (ideally every 3 months at the beginning of the study, up to every 6-8 months at a further stage of the study) These visits can coincide with the visits of the GCP monitor and/or the WP2 leader.

At least the following aspects should be checked:

- *Follow-up of previous visit*: Follow-up of any pendings from the previous visit/s.
- *Lab facility:* Check if there were any problems concerning the lab facility during the course of the study. These should be recorded in a notebook (*e.g*. problems with electricity)
- *Stock management and ordering of laboratory products*
    - Check that the laboratory products are stored in a suitable environment (dry, cool, protected from flooding/rodents, at the correct temperature - air conditioning, fridge - etc.)
    - Check that proper temperature monitoring is in place for storage room, fridges, freezers, incubators and laboratory. (Refer to SOP-WP6-QUAL-06 How to install and use a “Min/Max” thermometer). Temperature monitoring forms should be filed in the laboratory file.
    - Check that a good stock management system is in place for the laboratory products to be used for the Nidiag study: stock cards and monthly inventory of stock (Refer to SOP-WP6-QUAL-07-V01-24Sep2012 - Stock management). Monthly inventories should be stored in laboratory file.
    - Check that the “First in, first out” principle is used: use first the products that will expire first.
    - Check that there is a track record of what has been ordered (information is available depending on where ordering takes place: either at study coordination level or at country coordination level/field site).

    - Which items have been ordered, what was the quantity, at what time was the order done
    - Lot number and expiry dates of ordered items
    - If shipment of laboratory items: when did the shipment take place, proof of reception of the items, data on temperature monitoring (and if needed humidity monitoring) during shipment of items via a temperature logger
  - If index tests are ordered centrally (at study coordination level):
    - Check that a track record is available of what has been ordered (see previous point)
    - Check that 1 retention kit per lot of index tests is kept at the study coordination level
    - Check that version control of instructions for use (IFU) is being applied:
      - which lot has which IFU version number and date of issue
      - for every new version of IFU, adapt the related SOP if needed

- *Biological specimens*
  - Check that all biological specimens are handled as described in the applicable SOPs (*e.g.* biosafety). Check if the sample flow is running smoothly.
  - Check that all biological specimens are correctly labeled (Refer to SOP-WP6-DOC-02-V2.2-21December2012 – Numbering system).
  - Check that the required volume of samples is collected as described in the applicable SOPs (e.g. *SOP-WP2-LAB-44 – blood collection)*, and that this information is correctly recorded (in CRF / specific sampling forms).
  - Check that the required volume of left-over samples are collected in proper containers (*e.g.* 2 ml cryo-vials) according to applicable SOPs and that the stored samples are correctly recorded in the study specimen log stored in the laboratory file.
  - Check that the Study Specimen Log or equivalent document(s) is completed, accurate and coherent with the center's register and the CRF (check patient's number, date of specimen collection, type of specimen collected, whether the specimen was shipped or not, etc…).
  - Storage of biological specimens
    - Check that all biological specimens are properly stored until transport or until the end of the study (*e.g.* -70°C for left-over patient samples, incubator for blood cultures)
    - Check that a freezer inventory is available for the -70°C freezer, together with daily temperature monitoring records (laboratory file)
    - Check that the bacterial isolates are properly stored (Refer to SOP-WP6-LAB-01) until transport or until the end of the study.
  - Transport of biological specimens
    - Check that a packing list is filled out for each transport, and a copy of the packing list is stored in the laboratory file.
    - Check that the transport of biological specimens within the country (*e.g.* from study site to referral lab) or between countries (*e.g.* from study site to ITM Antwerp) is done according to applicable SOPs: triple packaging (biosafety), correct temperature (*e.g.* ice packs, temperature logger)
    - Check that the date and time of specimen shipment, the duration of transport and the date and time of specimen receipt at the reference lab is documented in the Study Specimen Log.

- *Laboratory testing*
  - Check that laboratory request forms are in place (which samples to be taken, which tests to be done, etc.) and are according to study protocol and applicable SOPs.
  - Check that the correct materials and reagents are used for the laboratory testing. None of the products should be expired.

- Check that country specific normal values of all relevant laboratory testing are available in the laboratory file and the Site Investigator file.
- Check that the tests are interpreted according to applicable SOPs.
- Check that test results are correctly recorded in laboratory notebooks and/or CRF.
- Check that all source data (test results) are correctly stored in lockable cabinets and can be traced for data source verification by the external monitor.
- Check that the results are reported to the PI in a correct and timely manner.

- *Index testing*
  - Check that the index tests are correctly handled and stored (Refer to SOP-WP6-QUAL-05 Handling and storage of RDTs)
  - Check that none of the index tests in use are expired.
  - Check that the index tests are only used for patients that are recruited in the study.
  - Check that the index test log is properly filled.
  - Check that the index tests are performed according to applicable SOPs (correct volume of sample, correct buffer, correct number of buffer drops, correct reading time).
  - Check that micropipettes are used instead of the blood transfer devices included in the RDT kits.
  - Check that the procedures for repeat testing and documentation of results (desiccant color change, presence of control line, presence of background, intensity of test lines) are correctly applied.
  - Check that results are correctly interpreted according to applicable SOPs.
  - Check that pictures are taken of the index tests, and if this data is securely saved on a computer (with back-ups).
  - Check that all source data (index test results) and CRFs are correctly stored (*e.g.* separately from the rest of the CRF if blinding of index tests results is required) and that they can be traced for data verification by the external monitor.

- *Quality control:*
  - Check that internal quality controls are used according to applicable SOPs:
    - Daily/weekly internal controls for biochemistry, hematology, serology etc.
    - ATCC strains and quality parameters for bacteriology (% contamination, % clinically significant organisms (CSO), volume of blood collected in blood culture bottles)
    - Second reading for specific tests (refer to specific SOPs)
  - Perform re-reading of positive microscopy slides (*e.g.* malaria slides, Gram stainings) and a percentage of negative microscopy slides (blinded of original results).
  - Check the report of the external quality assessment if the laboratory is participating in an EQA program (*e.g.* EQA for bacterial diagnostics for Nidiag sites in the first trimester of 2014).
  - Check the report of the quality audit if performed (to be agreed within TMG).
  - Check that all records of the quality controls are filed in the lab file.

- *SOPs*
  - Check that every test and every equipment has an up-to-date SOP.
  - Check that all versions of the SOPs are filed in the lab file.
  - Check that only the latest versions of the SOPs are used in the lab.
  - Check that the SOPs are reviewed and revised regularly.

- *CRF*
  - If applicable, check if the laboratory technician that fills the CRF is trained in Good Documenting Practice (GDP).

- Check that the laboratory part of the CRFs are properly filled in according to GDP.
- Check that the laboratory CRFs are securely stored until they are sent back to the PI.
- For fever study: Check that the index testing part of the CRF is separately stored from the rest of the CRF to be sent directly to the data entry clerk, without passing by the PI (blinding of index test results).
- Data source verification of the CRFs is done by the external monitor.

- *Equipment*
  - Equipment inventory
    - Check that an equipment inventory is in place for the equipment in the lab.
  - Maintenance plan
    - Check that a maintenance plan is in place for the equipment or if in the SOPs of the equipment it is mentioned how the equipment should be maintained and at which time points this maintenance should be done.
  - Service and repair
    - Check that a logbook is in place to record if an equipment is out of order, together with the actions taken and the outcome.
    - Check that if an equipment is out of order, this is clearly labeled on the machine.
  - Calibration
    - If applicable, check if calibration is performed when needed, and if a record of these calibrations is filed in the lab file.

- *Infection control and Waste management*
  - Check that the laboratory is clean, if the benches are disinfected daily, if the required personal protection is used (lab coats, gloves, masks, etc.).
  - Check that a site-specific procedure for waste management is in place and is correctly applied.
  - Check if the procedure for the management of expired products (SOP in preparation for Nidiag) is correctly applied.

- *Laboratory File*
  - Review the laboratory file and check if it is complete (Refer to SOP-WP6-DOC-03 Management of study documents): all SOPs, all quality control forms, all filled packing lists, study specimen log, index test log shipment information, normal values, etc.

A laboratory supervision report of the visits is prepared, documenting all the above, which is sent to the WP6 coordinator, the WP2 leader/delegate, the country coordinating investigator/delegate, and to the site Principal Investigator for follow-up on findings and corrective actions.

4. Definitions and Abbreviations

4.1 Definitions

Laboratory File (LF): The Laboratory File includes all essential documents related to the laboratory work at the site level. There is one Laboratory File per study site. The laboratory personnel is responsible for keeping it updated and for ensuring it is stored adequately.

Site Investigator File (SIF): The Site File includes all essential documents and forms related to the conduct of the study at the site level. There is one Site File per study site. The site investigators are responsible for keeping it updated and for ensuring it is stored adequately.

Quality Assurance (QA): All those planned and systematic actions that are established to ensure that the study is performed and the data are generated, documented (recorded), and reported in compliance with Good Clinical Practice (GCP) and the applicable regulatory requirement(s).

Quality Control (QC): The operational techniques and activities undertaken within the quality assurance system to verify that the requirements for quality of the trial-related activities have been fulfilled.

Source Document: These are original documents. They consist of data relevant to the current research such as medical records, administrative files, laboratory reports, consultation reports, pharmacy dispensation registers, etc...

4.2 Abbreviations

CRF: Case Report Form

EC: Ethics Committee

EQC: External Quality Control

GCP: Good Clinical Practice

GCLP: Good Clinical Laboratory Practice

GDP: Good Documenting Practice

IFU: Instruction For Use

IQC: Internal Quality Control

LF: Laboratory File

PI: Principal Investigator

RDT: Rapid Diagnostic Test

QM: Quality Manager

SIF: Site Investigator File

5. Records and archives

**Appendices & Forms for completion**	
**Number**	**Title**

6. Document History

**Revision**	

**Name and function**	**Date**	**Signature**
Author		
Barbara Barbé	23/02/2014	
Reviewed by		
Raffaella Ravinetto	17/04/2014	
Approved by		
Ninon Horié	06/05/2014	
