# Supplementary material for: Experiences and Lessons from a Multicountry NIDIAG Study on Persistent Digestive Disorders in the Tropics
Source: PLoS Negl Trop Dis. 2016 Nov 3;10(11):e0004818. doi: 10.1371/journal.pntd.0004818 (PMC5094778; doi:10.1371/journal.pntd.0004818)
Supplement: S8 Quality SOP — (PDF) [file pntd.0004818.s028.pdf]

	**SOP Title: How to install and use a "Min/Max" thermometer ?**
	Project/study: This SOP applies to all NIDIAG studies

1. Scope and application

This SOP describes the installation and the use of the Min/Max thermometer, in order to allow daily temperature monitoring by measuring the current temperature, the maximum and minimum temperature of a refrigerator, -20℃ freezer, incubator, or water bath, to ensure its performance.
A probe is submerged in a fluid (water for refrigerators, incubators or water baths ; glycerol for freezers) to avoid temperature fluctuations when opening the fridge/freezer's door.

2. Responsibilities

Function	Activities
Laboratory technician/Quality manager/Laboratory manager	- Installation of the Min/Max thermometer<br>- Daily temperature monitoring<br>- Daily temperature recording (SOP-WP6-QUAL-06-V1.1-04Feb2013-annex1-4)

3. Procedures

3.1 Materials

- Min/Max thermometer – 50℃/+70℃
- Water/Glycerol 87%
- Plastic tube, 10 ml
- Silicones/glue

3.2 Procedure

3.2.1. Preparation of the thermometer

1. Install the battery in the thermometer with correct polarity positioning (+ side up). **(If nothing appears on the display, replace the battery).**
2. Fill a plastic tube with water (for refrigerator/incubator) or glycerol (for freezer)

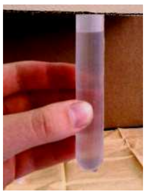

3. Make a hole in the plastic cap to allow the probe to pass. Submerge the probe of the thermometer in the plastic tube and close the cap. Fill up the cap with silicones/glue. Avoid air bulls. Let dry for 24 hours.

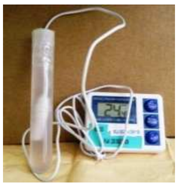

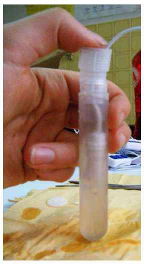

3.2.2 Installation of the thermometer

1. Attach the tube with the sensor inside the fridge/-20℃ freezer/incubator/water bath, at the middle of the equipment.
   <u>Comment</u>: The Min/Max thermometer **CANNOT** be used for -80℃ freezers!

2. Attach the thermometer on the outside of the equipment, by using the magnets on the back of the thermometer.

3.2.3 Temperature monitoring

1. Check that the temperature is displayed in ℃, if n ot, select “℃” with the red button “℃/℉” at the back of the thermometer.
2. Check that the temperature inside the equipment is displayed (“Fridge” has to be displayed above the temperature measurement). If not, select “Fridge” with the blue button “ROOM/FRIDGE”. (If “Room” is selected, the room temperature is displayed)

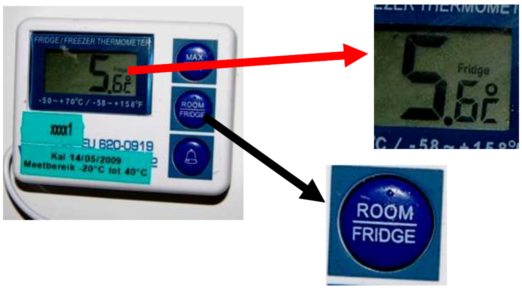

' with the
:" to display the
water bath.

3. Record the current temperature twice daily, once in the morning and once in the evening, on the Daily temperature registration form (SOP-WP6-QUAL-06-V1.1-04Feb2013-annex1-4).
4. Record every evening the maximum and minimum temperatures, by selecting the button “MAX/MIN” on the Daily temperature registration form (SOP-WP6-QUAL-06-V1.1-04Feb2013-annex1-3).

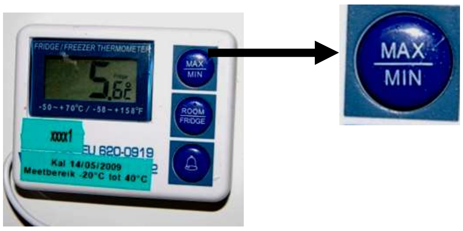

ıximum and
vith the

5. After recording of the actual temperature, the maximum and the minimum temperature, reset the min/max values to the current temperature. Press the "MAX/MIN" button for 2 seconds (until the second beep).
6. For -80℃ freezers, use the Daily temperature registrati on form (SOP-WP6-QUAL-06-V1.1-04Feb2013-annex4) to record the temperature that is displayed on the equipment itself twice daily. (Min/Max thermometer **CANNOT** be used for – 80℃ freezers!)

5. Records and archives

**Appendices and forms to complete**	
**Number**	**Title**
SOP-WP6-QUAL-06-V1.1-04Feb2013-annex1	Daily temperature registration form - Fridge
SOP-WP6-QUAL-06-V1.1-04Feb2013-annex2	Daily temperature registration form - Incubator
SOP-WP6-QUAL-06-V1.1-04Feb2013-annex3	Daily temperature registration form - Freezer -20℃
SOP-WP6-QUAL-06-V1.1-04Feb2013-annex4	Daily temperature registration form - Freezer -80℃

6. Documents and history

**Revision**	
SOP-WP6-QUAL-06-V01-19Sep2012	Initial version
SOP-WP6-QUAL-06-V1.1-04Feb2013	Translation in English

**Name and function**	**Date**	**Signature**
Author		
Barbara Barbé	04/02/2013	
Reviewed by		

Approved by		
Emilie Alirol	05/02/2013	
