# Supplementary material for: Experiences and Lessons from a Multicountry NIDIAG Study on Persistent Digestive Disorders in the Tropics
Source: PLoS Negl Trop Dis. 2016 Nov 3;10(11):e0004818. doi: 10.1371/journal.pntd.0004818 (PMC5094778; doi:10.1371/journal.pntd.0004818)
Supplement: S9 Quality SOP — (PDF) [file pntd.0004818.s029.pdf]

	**SOP Title: Stock management**
	Project/Study: This SOP applies to all NIDIAG WP2 clinical studies.

1. Scope and application

This SOP describes the stock management of consumables and materials that are used for NIDIAG, by using stock cards and performing monthly inventories.

2. Responsibilities

Function	Activities
Laboratory technician / Laboratory manager	- Stock tracking of consumables and materials.<br>- Management of machines.<br>- Recording of entries and exits of stock on the stock cards.<br>- Monthly physical inventory of all stock.

3. Procedures

3.1 Materials

- Stock card (cf. SOP-WP6-QUAL-07-V1.1-04Feb2013-annex1)
- Monthly inventory form (manual recording on print-out of document or computer file (cf. SOP-WP6-QUAL-07-V1.1-04Feb2013-annex2)

3.2 Procedure

3.2.1 Stock cards

1. Record all the entries ("IN") of the consumables or materials on the stock cards. **Use 1 stock card per product and per expiry date.**
2. Record all exits ("OUT") of consumables or materials from the stock to the laboratory on the stock cards.

   Comment:

   - Record each time a box is removed from the stock and sent to the laboratory.
   - Record the number of tests, not the number of boxes that are removed.

   E.g. malaria RDTs: packaging is 25 tests per box. The exit of 2 boxes of malaria RDTs is recorded as an exit of 50 tests (units).

3.2.2 Monthly inventory

1. Do a physical count of all reagents and consumables at the end of each month and fill out the inventory form, sheet "consumables" of SOP-WP6-QUAL-07-V1.1-04Feb2013-annex2:
   - Record the stock that was present at the beginning of the month (= physical count at the end of last month)
   - Record the stock received during the month (= products delivered during that month)
   - Record the stock still present at the end of the month (= physical count at the end of this month)

   Comment: the stock is recorded per test (not per box), per bottle (not per kit), etc.
2. Calculate the consumption of the consumables of each month by using the following formula:

   Consumption of this month =

(Initial stock at beginning of the month + Stock received during the month) – Final stock at the end of the month

3. Do a physical count of the materials at least once every 3 months and record the information on the sheet "materials" of the same document (SOP-WP6-QUAL-07-V1.1-04Feb2013-annex2). Do not calculate consumption for materials: materials are re-usable (e.g. micropipette). Record when a material breaks down or is out of use under "comments".

4. Record the machines in use for the NIDIAG on the sheet "machines" of the document (SOP-WP6-QUAL-07-V1.1-04Feb2013-annex2). Record if the machine functions properly, or – if not – what are the problems and which actions have been taken. Regular maintenance activities can be recorded under "comments". Fill out the form every month.

4. Records and archives

**Appendices and forms to complete**	
**Number**	**Title**
SOP-WP6-QUAL-07-V1.1-04Feb2013-annex1	Stock card
SOP-WP6-QUAL-07-V1.1-04Feb2013-annex2	Excel document for stock inventory

5. Documents and history

**Revision**	
SOP-WP6-QUAL-07-V01-24Sep2012	Initial version
SOP-WP6-QUAL-07-V1.1-04Feb2013	Translation in English

**Name and function**	**Date**	**Signature**
Author		
Barbara Barbé	04/02/2013	
Reviewed by		
Approved by		
Emilie Alirol	07/02/2013	
