# Supplementary material for: Experiences and Lessons from a Multicountry NIDIAG Study on Persistent Digestive Disorders in the Tropics
Source: PLoS Negl Trop Dis. 2016 Nov 3;10(11):e0004818. doi: 10.1371/journal.pntd.0004818 (PMC5094778; doi:10.1371/journal.pntd.0004818)
Supplement: S10 Quality SOP — (PDF) [file pntd.0004818.s030.pdf]

	**SOP Title : Handling of expired and disqualified products**
	Project/Study : This SOP is applicable to all NIDIAG studies

1. Scope and application

This document gives instructions on how to handle expired products and products that are disqualified as “unfit for purpose” (drugs, laboratory reagents, RDT kits, etc.) in the NIDIAG studies.

2. Responsibilities

Function	Activities
Laboratory technician	• Collect all expired or disqualified laboratory products.<br>• Centralize all expired laboratory products in the central stock of the site.
Clinician	• Collect all expired drugs.<br>• Centralize all expired drugs in the central stock of the site.
Site investigator	• Ensure that all expired or disqualified products are packed in a box marked “Out of use”.<br>• Transport all expired and disqualified products once a month to the coordination site (if applicable).
Principal investigator	• Receive and store all expired and disqualified products at the central stock of the coordination site as “Out of use” until the end of the study.<br>• Use a logbook to register all expired and disqualified products.<br>• Keep the logbook up to date.

3. Procedures

3.1 Procedure on the site

- Always verify the expiration date before using a product (laboratory reagent, RDT kit, drug, etc.)
- If expired or disqualified, do not use the product! Store the product in a box marked with “OUT OF USE!” in the central stock of the site.
- At the end of the month, transport the box(es) with the expired and disqualified products to the coordination site (if applicable).

3.2 Procedure at the country coordination level

- Use a logbook to register all expired and disqualified products (See example in **Annex 1**).
- Receive the expired and disqualified products. Update the logbook after each reception.
- Store the products at the central stock of the coordination site in a box(es) marked “OUT OF USE!”
- Record the reason for classifying the products as “Out of use” in the logbook under “Reason” (*e.g.* expired product, product unfit for purpose (refer to a report))
- If expired products are used for training or educational purposes, note this in the logbook under “Comments”.
- Destroy the expired and disqualified products according to local waste management procedures at the end of the NIDIAG study.

4. Records and archives

**Appendices and forms to complete**	
**Number**	**Title**
SOP-WP6-QUAL-08-Annex 1	Logbook for expired and disqualified products

5. Document and history

**Revision**	
**SOP-WP6-QUAL-08-V1.0-05Mar2014**	Initial version

**Name and function**	**Date**	**Signature**
Author		
Barbara Barbé	05/03/2014	
Revised by		
Jan Jacobs	05/03/2014	
Approved by		
Ninon Horié	07/03/2014	
