# Supplementary material for: Experiences and Lessons from a Multicountry NIDIAG Study on Persistent Digestive Disorders in the Tropics
Source: PLoS Negl Trop Dis. 2016 Nov 3;10(11):e0004818. doi: 10.1371/journal.pntd.0004818 (PMC5094778; doi:10.1371/journal.pntd.0004818)
Supplement: S11 Quality SOP — (PDF) [file pntd.0004818.s031.pdf]

| | |
|---|---|
| | **SOP Title:** Handling and storage of RDTs |
| | Project/study: This SOP applies to the NIDIAG Digestive syndrome study. |

## 1. Scope and application

This SOP describes the storage and handling of RDTs. This SOP focuses on, but is not limited to, the Index tests that are evaluated for the NIDIAG digestive syndrome study.

## 2. Responsibilities

| Function | Activities |
|---|---|
| Laboratory Technician | - Comply to this SOP<br>- Ensure correct storage of the RDTs<br>- Ensure correct stock management of the RDTs<br>- Keep the Index test log form up-to-date<br>- Daily temperature recording of storage room |
| Laboratory Manager | - Supervision of the activities mentioned above |
| Quality Manager | - Verify correct storage of RDTs<br>- Verify Index test log form<br>- Verify stock management of the RDTs<br>- Propose corrective actions if inconsistencies to this SOP are noticed. |

## 3. Procedures

### 3.1 Materials

- Forms described in “5. Records and archives”
- Min-Max thermometer
- Lockable cupboard
- Biohazard waste container

### 3.2 Procedures

#### 3.2.1 Handling of RDTs

**3.2.1.1 General regulations**

1) Wear gloves.
2) Handle all specimens as potentially infectious.
3) Bring all reagents/components to room temperature before testing.
4) Do not use the test device after expiry date.
5) Do not use the test device if the foil pouch is damaged or if the seal is broken.
6) Keep the pouch sealed until use.
7) Perform the test immediately after opening of the pouch.
8) Check the desiccant that is included in the pouch for colour change. Discard the device if the colour has changed (= desiccant is saturated). Use a new test device.
9) Label the test device (or the tube in case of a dipstick) with the patient’s ID number and date.
10) Do not mix components from different kits/lot numbers.
11) Some buffers (assay diluents) contain sodium azide as preservative. Avoid direct skin contact!
12) Do not replace the buffer (assay diluent) by other fluids.
13) Do not reuse the test device.
14) Discard used test devices, samples and potentially contaminated materials in a biohazard waste container.

#### 3.2.1.2 Specific handling and procedures for Nidiag RDTs

Refer to the specific Nidiag SOPs for the handling and the procedures of the specific RDTs used for the Nidiag studies.

### 3.2.2 Storage of RDTs

#### 3.2.2.1 General storage conditions for RDTs

1) Store the RDT kits in a dry and cool place, protected from direct sunlight.
2) Store the RDT kits according to the temperature ranges specified by the manufacturer.
3) DO NOT freeze RDTs or kit components.
4) Some RDTs can be stored in the fridge. Bring the tests to room temperature before use.

#### 3.2.2.2 Specific storage conditions for index tests

1) Store the index tests in a locked cupboard.
2) Restrict access of the cupboard to authorized Nidiag personnel only.
3) Comment: Only use the index tests for patients enrolled in the Nidiag studies.
4) Store the index tests according to the temperature ranges in **table 1**. Try to avoid as much as possible temperatures above the upper temperature limit.
5) Perform twice daily temperature recording of the storage room. Use the Temperature Registration Form in **annex 1**. If the index tests are stored in the fridge, use SOP-WP6-QUAL-06-annex1
6) Comment: Refer to **SOP-WP6-QUAL-06** for installation of Min-Max thermometer and temperature recording. The probe of the Min-Max thermometer can but does not have to be put in a tube with water when used to record the room temperature.
7) Record the room temperature of the room where the index testing takes place in the CRF, when performing the index tests.

**Table 1. Temperature ranges for storage of Index tests**

| Index test | Temperature range |
|---|---|
| Urine CCA Test | 4-28°C |
| Crypto-Giardia Duo-strip | 4-30°C |

### 3.2.3 Stock management and accountability of Index tests

#### 3.2.3.1 Index log form

1) Record for each index test which lot numbers were used for which patients in the Index Log Form in **annex 2**.
2) Keep the Index Log Form up-to-date.
3) File the Index Log Form in the Lab file.

#### 3.2.3.2 Stock cards and monthly inventories

1) Use a stock card for each lot number of each index test, according to **SOP-WP6-QUAL-07**.
2) Do a physical count of the index tests at the end of each month and fill out the inventory form, of **SOP-WP6-QUAL-07-annex2** (sheet “consumables”), according to **SOP-WP6-QUAL-07.**

## 4. Definitions

RDT = Rapid Test Diagnostic
CRF = Case Report Form

## 5. Records and archives

| Appendices & Forms for completion | |
|---|---|
| **Number** | **Title** |
| CRF digestive | |
| SOP-WP6-QUAL-05-V01-08Feb2013-annex1<br>SOP-WP6-QUAL-08-V1-12Dec2013-annex2<br>SOP-WP6-QUAL-06-V1.1-04Feb2013-annex1<br>SOP-WP6-QUAL-07-V1.1-04Feb2013-annex1<br>SOP-WP6-QUAL-07-V1.1-04Feb2013-annex2 | Temperature registration form – Room temperature<br>Index log form<br>Temperature registration form - Fridge<br>Stock card<br>Stock inventory form |

## 6. Document History

| Revision | |
|---|---|
| SOP-WP6-QUAL-09-V1-26May2014 | Initial version |

| Name and function | Date | Signature |
|---|---|---|
| *Author* | | |
| *Barbara Barbé* | 20/08/2013 | |
| *Reviewed by* | | |
| *Pascal Mertens* | 11/03/2014 | |
| *Approved by* | | |
| *Ninon Horié* | 26/05/2014 | |
