# Supplementary material for: Experiences and Lessons from a Multicountry NIDIAG Study on Persistent Digestive Disorders in the Tropics
Source: PLoS Negl Trop Dis. 2016 Nov 3;10(11):e0004818. doi: 10.1371/journal.pntd.0004818 (PMC5094778; doi:10.1371/journal.pntd.0004818)
Supplement: S1 Data management SOP — (PDF) [file pntd.0004818.s032.pdf]

No. 32 – Data management SOP: Completing case report forms (CRFs)

nidiag<br>Better Diagnosis of Neglected Infectious Diseases	**SOP Title:** Procedure for completion of Case Report Forms
	Project/study: NIDIAG

1. Scope and application

The Case Report Form (CRF) contains all the data collected during the study. There is one CRF per patient. The aim of this SOP is to describe how CRFs should be completed and corrected . It applies to all NIDIAG studies and to all personnel involved with the on-site completion and verification of the CRFs.

2. Responsibilities

**Function**	**Activities**
Site investigator (or delegated staff e.g. nurses)	- Fill in the CRF according to the SOP<br>- Ensure the legibility, completeness, correctness of the data and consistency with the source documents (patient medical records, laboratory and clinical test results)<br>- Store the CRF in appropriate place<br>- Correct errors reported by the study Monitor or Site Quality Manager
Site Quality Manager and External Monitor	- Verify that the data are accurate, complete and up-to-date<br>- Report mistakes and discrepancies to the investigator<br>- Ensure errors detected during monitoring are corrected by

	the investigator

3. Procedures

3.1 Subject privacy

- The CRF is an anonymous document. The patient's name, telephone or address must not appear on the CRF. The only identifiers present on the CRF are the patient number, his/her initials, date of birth and his/her age.
- It is possible to file copies of the source documents (e.g. medical record, lab results) in the CRF only if these copies are anonymized.
- Do not file the informed consent in the CRF.

3.2 Completing the CRF

- Use a ballpoint pen to fill in the CRF.
- All entries should be in CAPITAL LETTERS.
- Only enter results in the fields provided.
- Make sure the data collected in the CRF are consistent with the source documents.
- Do not leave questions unanswered. If an answer is not known, mark "NK" (Not Known). If a procedure is not done, enter ND (Not Done). If a question is not applicable, mark "NA" (Not Applicable).
- The CRF should be kept up-to-date while the patient is in the study.
- Sign and date the CRF each time a visit is completed. By doing so, you take responsibility for the correctness and accuracy of the data.

3.3 Correcting the CRF

- Each correction in the CRF must be dated and initialled (or signed).
- The original entry should remain legible. Do not use any correction fluid or pen to erase the entry you wish to modify. Draw through the error with a single line and write the correct answer next to the original entry.

- The instructions for completion and correcting of the CRF will be added in the first page(s) of the CRF. Depending on the study progress or study, amendments to these instructions or other instructions might be added.

3.4 Storage and access

- CRFs must be kept in a locked and safe place. The access should be strictly restricted to the study staff.
- The CRF must be retained with regard to local legislation and for at least 2 years after the end of the study.

4. Definitions and abbreviations

- CRF= Case Report Form: a printed document designed to record all the protocol required data.
- Investigator (or Site investigator): A person who is responsible for the conduct of a clinical trial at a trial site.
- Site quality manager: A person who is responsible for ensuring quality systems are applied at each step of the NIDIAG studies on a day-to-day basis. He/She oversees all research activities and makes sure that studies are conducted in accordance with the protocol, SOPs, GCP/GCLP and national regulations.
- Monitor: The monitor is a person who is responsible for verifying that the study is conducted, recorded and reported in accordance with the study protocol, the Good Clinical Practice (GCP), and the Standard Operating Procedures (SOPs).
- Source documents: These are original documents. They consist of data relevant to the current research such as medical records, administrative files, laboratory reports, consultation reports, pharmacy dispensation registers, etc...

5. Records and archives

**Appendices & Forms for completion**

Number	Title
1	Case Report Form

6. Document History

**Revision**	
NA	*NA*

Name and function	Date	Signature
Author		
Veerle Lejon	01/02/2012	
Reviewed by		
Hary van Loen		
Approved by		
Emilie Alirol	01 February 2012	
