# Supplementary material for: Experiences and Lessons from a Multicountry NIDIAG Study on Persistent Digestive Disorders in the Tropics
Source: PLoS Negl Trop Dis. 2016 Nov 3;10(11):e0004818. doi: 10.1371/journal.pntd.0004818 (PMC5094778; doi:10.1371/journal.pntd.0004818)
Supplement: S2 Data management SOP — (PDF) [file pntd.0004818.s033.pdf]

No. 33 – Data management SOP: Procedures for data entry management

	**SOP Title:** Procedures for data entry management in NIDIAG studies
	Project/study: This SOP applies for the NIDIAG WP2 studies, in particular the Digestive study

Scope and application

In order to protect research participant's confidentiality, all research records should remain anonymous. All personal information (i.e. names, telephone number, address, etc…) should be removed from the CRF, labels and other study documents, and should be replaced by a unique patient study number. This unique number is attributed upon inclusion of a research participant in a study and allows for the tracking of his/her medical information and biological specimens. This SOP describes how unique patient study numbers are created and how patient's specimens are numbered and labelled.

Responsibilities

**Function**	**Activities**
Site investigator	• Attributing a unique study number to each patient included in the study in accordance with this SOP<br>• Establishing a patient identification list detailing the correspondence between patient's study number and patient's name<br>• Ensuring the identification list is kept in a secure place and that access to it is restricted to the site investigator's team<br>• Ensuring that the patient study number is consistent throughout all study documents and all study samples
Site investigator or	• Attributing a unique study specimen number to each specimen collected during the study in accordance with this

Lab technician	SOP<br>• Establishing a study specimen log detailing the study specimen number, the patient study number, the type of biological sample, and the date and time of collection
Quality manager (except in DRC)	• Verifying that this SOP is complied with<br>• Verifying that the patient identification list and the study specimen log are correct, up-to-date, and securely stored<br>• Verifying that the patient's study number is consistent throughout all study documents and all study samples<br>• Verifying that study samples are identified and labelled in accordance with the study protocol, the patient identification list and this SOP

Patient Identification List

- List all patients potentially eligible to be included in the study in the "Patient Identification List" (see figure 1). There is one "Patient Identification List" per syndrome and per centre.
- Indicate the following information:

1) Patient's name, age and sex
2) Whether the patient was included in the study or not
3) The reason for non-inclusion if applicable (ex: refused to participate, younger than 5 years old, etc...)
4) The patient study number (only patients included in the study get a study number, see below)
5) The date of inclusion in the study

Patient identification list

Country & study centre (name, number): Nepal, Dankuta Hospital (61)

Row nr	Patient's name (Last, First)	Age *(years)*	Sex (M/F)	Included (yes /no)	Reason for non inclusion	Patient Study n°	Inclusion date *(dd/mmm/yyyy)*
1	Dupont Francis	41	M	yes	na	61001-Fx	14/07/2012
2	Piccard Marie	4	F	no	The patient is below 5	na	na
3							
4							
5							

Figure 1: Example of patient identification list for the Fever Syndrome

Patient study number

- Give a unique patient study number to each patient included in one of the NIDIAG study.
- The patient study number consists of 4 fields:

1) Country number (1 digit, see list table 1)
2) Center's number (1 digit, see list in table 1)
3) Patient order number (3 digits)
4) NIDIAG syndrome code (2 letters see list in table 2)

Ex: for the first patient included in the Neurological Syndrome clinical study in Mosango , the patient study number is 21001-Nx.

The patient study number is attributed to the patient after inclusion in the study, i.e. after checking inclusion/exclusion criteria and after the patient has signed the informed consent

Field 1 and 2: Country Number & Centre's number

The first digit of the patient study number indicates the country where the patient was included. The second digit of the patient study number indicates the study site where the patient was included.

Table 1: List of country and centre numbering (2 first digits of patient study number)

Country number	Centre Number	Country	Study site
1	1	Cambodia	Sihanouk Hospital Center of HOPE
2	1	DR Congo	Hôpital rural de Mosango
3	1	Indonesia	Tulehu Hospital

3	2	Indonesia	Tulehu Health Center
4	1	Ivory Coast	Hôpital Méthodiste de Dabou
5	1	Mali	INRSP Reference Lab of Parasitology, Bacteriology and Virology
5	2	Mali	Niono Health Center
6	1	Nepal	Dhankuta Hospital
6	3	Nepal	BPKIHS
7	1	Sudan	Tabarak Allah Hospital

Field 3: Patient order number

In each centre, every patient included in the study (irrespective of the syndrome) gets an order number, consisting of 3 digits.

The first patient included gets number 001, the second 002 etc..

Field 4: Syndrome

The last field of the patient number consists of letters indicating the syndrome for which the patient is included.

Table 2: List of syndromes and their letter

Letter	Syndrome
Nx	Neurological
Fx	Fever
Dx	Digestive
FN or NF	Fever and Neurological

Examples

1) The first ‘case’ patient enrolled in Hôpital Méthodiste de Dabou in Ivory Coast gets number 41001. He is included in the Digestive syndrome clinical study, his complete patient study number becomes: 41001-Dx.

2) The second ‘case’ patient enrolled in Hôpital Méthodiste de Dabou in Ivory Coast gets number 41002. He is included in the Digestive syndrome clinical study, his complete patient study number becomes: 41002-Dx.

Field 5: Number for control patients in the Digestive study :

From the moment a patient number for an enrolled ‘case’ is assigned, then a suitable ‘control′ patient (same gender, age group, region) to the enrolled case is searched. This suitable control patient will be assigned with the same patient number as its linked ‘case’, but with an additional number: 01 (or 02 or 03… if applicable)

For example: for an enrolled case patient 41001-Dx a suitable control patient with number 41001-Dx-**01** will be assigned. For an enrolled case patient 41002-Dx a suitable control patient with number 41002-Dx-**01** will be assigned etc… However if for the control patient during the recruitment process, stool (and urine) sampling is not performed, or when abdominal pain or diarrhoea is developed, then a new suitable ‘control’ patient has to be found .E.g. if 41001-Dx-01 is not suited, then 41001-Dx-**02** will be assigned as a control to 41001-Dx. If 41002-Dx-01 is not suited, then 41002-Dx-**02** will be assigned as a control to 41002-Dx., **….**

Study specimen number

All samples collected from a study patient during the study period should be identified, at all times, by a unique study specimen number. This applies to samples used for the index tests, for the reference tests, and for long-term storage.

The study specimen number consists of:

1) the patient study number

2) the specimen type abbreviation (2 letters, see table 3 below)

3) the specimen number out of the total number of specimens collected (important when more than one tube is collected).

Table 3: List of sample types and their abbreviation

**Specimen type**	**Specimen type abbreviation**
**Ur**ine	UR
**St**ool	ST

Examples

The first 'case' patient included in Hôpital Méthodiste de Dabou in Ivory Coast gets number 41001. He is included in the Digestive syndrome, his complete patient study number is: 41001-Dx. A suitable 'control' patient will be assigned a complete patient study number 41001-Dx-01.

- Stool is collected, in accordance with the study protocol:
- and is numbered per visit as ST1, ST2 (if applicable) or ST3 (if applicable).

For case patient 41001-Dx following samples might be prepared :

- at **visit 1**: 41001-Dx-**ST1**
- at **visit 2** (if applicable): 41001-Dx-**ST2**
- at **Visit 3** if applicable): 41001-Dx-**ST3**

For control patient 41001-Dx-01 the sample 41001-Dx-01-**ST1** (**at Visit 1**) will be prepared.

- Preparation of aliquots on left-over stool are numbered per visit with addition of a letter a, b,c:

For case patient 41001-Dx :

- at **visit 1**: If 3 aliquots are prepared from a sample, then they are numbered 41001-Dx-**ST1a** and 41001-Dx-**ST1b** and 41001-Dx-**ST1c**

- at **visit 2** (if applicable): If 3 aliquots are prepared from a sample, then they are numbered 41001-Dx-**ST2a** and 41001-Dx-**ST2b** and 41001-Dx-**ST2c**
- at **visit 3** (if applicable): If 3 aliquots are prepared from a sample, then they are numbered 41001-Dx-**ST3a** and 41001-Dx-**ST3b** and 41001-Dx-**ST3c**

For control patient 41001-Dx -01

- at **visit 1**: If 3 aliquots are prepared from a sample, then they are numbered 41001-Dx-01-**ST1a** and 41001-Dx-01-**ST1b** and 41001-Dx-01-**ST1c**
- Urine is also taken (in Côte d'Ivoire and Mali only) and is numbered per visit as UR1, UR2 (if applicable) or UR3 (if applicable) as follows:

For case patient 41001-Dx following samples might be prepared : 41001-Dx-**UR1** (**at Visit 1**); 41001-Dx-**UR2** (**at Visit 2** if applicable)); 41001-Dx-**UR3** (**at Visit 3** if applicable)).

For control patient 41001-Dx-01 following samples will be prepared : 41001-Dx-01-**UR1** (**at Visit 1**).

If aliquots for urine are stored, then use the same numbering procedure as for stools (see above)

Study Specimens Log

All specimens collected from a study patient during the study period should be recorded in a "Study Specimens Log" (see figure 2). There is one Study Specimen Log per Syndrome per centre.

The following information should be indicated:

1) Patient's Study Number
2) Type of sample
3) Date of collection
4) Time of collection
5) Study Specimen label (only applicable when using excel sheets)
6) Person who performed the collection
7) Date of shipment
8) Organization to whom the sample is shipped
9) Person to whom the sample is shipped

10) Date of receipt of sample

11) Comments (if applicable)

Study specimen log											
Country & study centre (name, number): RD Congo, Mosango (21)											
Row nr	Patient study n°	Sample type (__ _)	Date collection (dd/mmm/yyyy)	Time collection (hh:mm)	Study specimen label	Collected by	Date of shipment (dd/mm/yyyy)	Sent to Organization	Sent to person	Date of receipt	Comments
1	21001-Nx	HE1	12/Mar/2012	10:24	21001-Nx-HE1-12/Mar/2012	nurse x	16/03/2012	INRB	Lunguya	17/03/2012	
2	21001-Nx	BD1	12/Mar/2012	10:24	21001-Nx-BD1-12/Mar/2012	nurse x	na	na	na	na	
3	21001-Nx	BH1	12/Mar/2012	10:25	21001-Nx-BH1-12/Mar/2012	nurse x	na	na	na	na	
4	21001-Nx	UR1	12/Mar/2012	10:45	21001-Nx-UR1-12/Mar/2012	nurse x	na	na	na	na	
5	21001-Nx	PH1	12/Mar/2012	11:00	21001-Nx-PH1-12/Mar/2012	tech y	na	na	na	na	
6	21001-Nx	PH2	12/Mar/2012	11:00	21001-Nx-PH2-12/Mar/2012	tech y	16/03/2012	ITM	Jan Jacobs	17/03/2012	
7	21001-Nx	CS1	12/Mar/2012	12:05	21001-Nx-CS1-12/Mar/2012	Dr X	na	na	na	na	
8	21001-Nx	CS2	12/Mar/2012	12:05	21001-Nx-CS2-12/Mar/2012	Dr X	na	na	na	na	
9	21001-Nx	CS3	12/Mar/2012	12:05	21001-Nx-CS3-12/Mar/2012	Dr X	16/03/2012	ITM	Jan Jacobs		lost
10	21001-Nx	CS4	12/Mar/2012	12:05	21001-Nx-CS4-12/Mar/2012	Dr X	16/03/2012	ITM	Jan Jacobs	17/03/2012	
11	21001-Nx	SE1	12/Mar/2012	15:56	21001-Nx-SE1-12/Mar/2012	tech y	na	na	na	na	
12	21001-Nx	SE2	12/Mar/2012	15:56	21001-Nx-SE2-12/Mar/2012	tech y	16/03/2012	ITM	Jan Jacobs	17/03/2012	tube damaged
13											
14											

Figure 2: An example of Study Specimen Log. This example matches the one given above under 5.1. The serum and plasma samples have been processed from the blood collected on dry tube or heparine as indicated under "comments". For shipped date of shipment, receiving organization and person and confirmation of receipt are indicated. The other samples have been analysed on site, therefore, "not applicable (na)" is filled in.

Study specimen labelling

The study specimen label consists of 2 fields:

1) Specimen identification number (such as: 21001-Nx-CS1 and 61014-Fx-PH1)

2) Collection Date Format: DD-MMM-YYYY for example: 13-FEB-2012

MMM is the English abbreviation of the month, listed in table 4.

Table 4: List of the abbreviations to be used for the months

Month	**Abbreviation**
**January**	JAN
**February**	FEB
**March**	MAR

**April**	APR
**May**	MAY
**June**	JUN
**July**	JUL
**August**	AUG
**September**	SEP
**October**	OCT
**November**	NOV
**December**	DEC

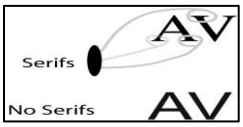

Study sites are responsible for printing study specimen labels. The writing should be clear, in dark permanent ink and block capital letters (do not use inkjet printers since this ink will be dissolved when it comes in contact with water).

The size of the letter type on the label should be at least 9-points.

The letter type of the label is preferentially a “sans serif” letter type (see figure) such as Arial

Align text left

Examples of labels

1) A label for blood EDTA sample n°1 collected from patient 115 in Tabarak Allah (Sudan) during the fever syndrome study. Date of collection 23 August 2012:

71115-Fx-BE1
23-AUG-2012

2) A label for urine sample n°1 collected from patient 89 in Mosango (DR Congo) during the neurological syndrome study. Date of collection 2 June 2013:

21089-Nx-UR1<br>02-JUN-2012

Correct label placement

- <u>Large tubes (longer than label):</u>

1) Hold sample tube horizontally with cap in left hand.
2) Affix patient label to be read from left to right, starting below tube cap (directly over manufacturer label).
3) Label as high as possible on tube (To allow for maximum length of uncovered tube at bottom, facilitating placement of tubes in racks etc.)

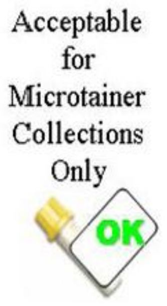

- <u>Small tubes (shorter than label, for example cryovials):</u>

1) Hold sample tube vertically.
2) Affix label, making sure that text is not overlapped.
3) If using paper labels on tubes that will be cooled or frozen: cover the label completely with transparent tape (the tape should overlap the complete label) to avoid that the label comes off the tube.

Records and Archives

**Appendices & Forms for completion**	
**Number**	**Title**
1	Patient Identification List
2	Study Specimens Log

Document History

**Revision**	
SOP-WP6-DOC-02-V1.0-25Jun2012	Initial version

SOP-WP6-DOC-02-V1.1-09Jul2012	Translation in French
SOP-WP6-DOC-02-V2.0-21Dec2012	Addition of specimen abbreviations used in the fever syndrome
SOP-WP6-DOC-02-V2.1-18Sep2012	Addition of annexes to the French version
SOP-WP6-DOC-02-V3.0-22April2013	Deletion of Yassa Bonga and Koshi Zonal Hospital sites<br><br>Modification of Annex 2
SOP-WP6-DOC-02-V3.1-22Apr2013	Addition of specimen abbreviations used in the fever syndrome to the French version<br><br>Correction of spelling and grammar errors
SOP-WP6-DOC-02-V4.0-02JUN2014	Adapting SOP in particular to Digestive study
SOP-WP6-DOC-02-V5.0-13JUN2014	Correction of Study site (table 1) & syndrome letters (table 2)

**Name and function**	**Date**	**Signature**
Author		
Harry van Loen	13.06.2014	
Revised by		
Barbara Barbé		
Approved by		
Ninon Horie		
